# Molecular *cyclo*-P_3_ complexes of the rare-earth elements *via* a one-pot reaction and selective reduction†

**DOI:** 10.1039/d2sc06730g

**Published:** 2023-02-03

**Authors:** Adrian Hauser, Luca Münzfeld, Sören Schlittenhardt, Ralf Köppe, Cedric Uhlmann, Ulf-Christian Rauska, Mario Ruben, Peter W. Roesky

**Affiliations:** a Institute of Inorganic Chemistry, Karlsruhe Institute of Technology (KIT) Engesserstraße 15 D-76131 Karlsruhe Germany roesky@kit.edu; b Institute of Nanotechnology, Karlsruhe Institute of Technology (KIT) Hermann-von-Helmholtz-Platz 1 D-76344 Eggenstein-Leopoldshafen Germany; c Centre Européen de Science Quantique (CESQ), Institut de Science et d'Ingénierie Supramoléculaires (ISIS, UMR 7006), CNRS-Université de Strasbourg 8 allée Gaspard Monge BP 70028 67083 Strasbourg Cedex France; d Institute of Quantum Materials and Technologies (IQMT), Karlsruhe Institute of Technology Hermann-von-Helmholtz-Platz 1 76344 Eggenstein-Leopoldshafen Germany

## Abstract

Synthesis of new organo-lanthanide polyphosphides with an aromatic *cyclo*-[P_4_]^2−^ moiety and a *cyclo*-[P_3_]^3−^ moiety is presented. For this purpose, the divalent Ln^II^-complexes [(NON)Ln^II^(thf)_2_] (Ln = Sm, Yb) ((NON)^2−^ = 4,5-bis(2,6-diisopropylphenyl-amino)-2,7-di-*tert*-butyl-9,9-dimethylxanthene) and trivalent Ln^III^-complexes [(NON)Ln^III^BH_4_(thf)_2_] (Ln = Y, Sm, Dy) were used as precursors in the reduction process of white phosphorus. While using [(NON)Ln^II^(thf)_2_] as a one-electron reducing agent the formation of organo-lanthanide polyphosphides with a *cyclo*-[P_4_]^2−^ Zintl anion was observed. For comparison, we investigated a multi-electron reduction of P_4_ by a one-pot reaction of [(NON)Ln^III^BH_4_(thf)_2_] with elemental potassium. As products molecular polyphosphides with a *cyclo*-[P_3_]^3−^ moiety were isolated. The same compound could also be obtained by reducing the *cyclo*-[P_4_]^2−^ Zintl anion within the coordination sphere of Sm^III^ in [{(NON)Sm^III^(thf)_2_}_2_(μ-η^4^:η^4^-P_4_)]. Reduction of a polyphosphide within the coordination sphere of a lanthanide complex is unprecedented. Additionally, the magnetic properties of the dinuclear Dy^III^-compound bearing a bridging *cyclo*-[P_3_]^3−^ moiety were investigated.

## Abstract 2

Synthesis of new organo-lanthanide polyphosphides with an aromatic *cyclo*-[P_4_]^2−^ moiety and a *cyclo*-[P_3_]^3−^ moiety is presented.

## Graphical abstract

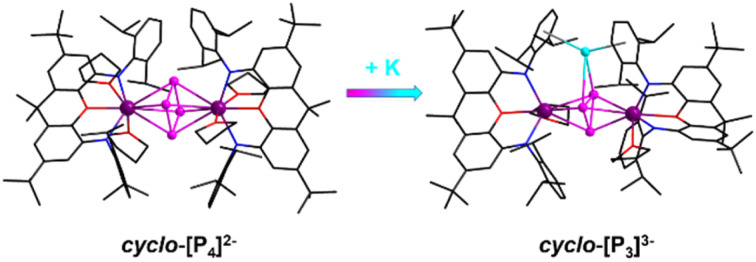

## Introduction

**Fig. 1:**
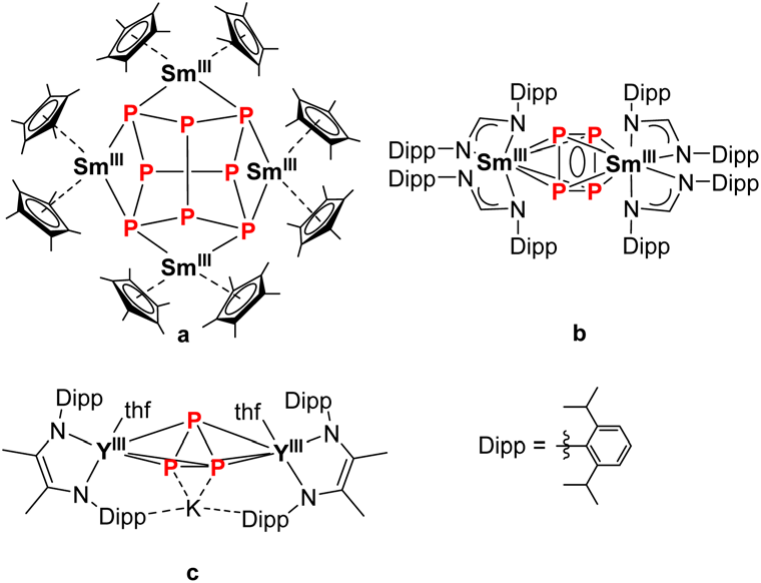
Selection of various rare earth polyphosphides with different [P_*x*_]^*n*−^ Zintl anions.^15,30,36^

In recent decades, the functionalization of white phosphorus (P_4_) using organometallic compounds has been intensively studied.^1^ These metal polyphosphides play an important role as intermediates in the production of organophosphorus compounds since they can circumvent the use of environmentally harmful chlorine gas.^2,3^ Starting with the activation of P_4_ using transition metals which led to the formation of various polyphosphide ring structures such as [P_4_]^2−^, [P_5_]^−^ or the neutral [P_6_]-ring,^1,4–7^ this reactivity could be transferred to a variety of main group compounds in the following years.^1,8–14^ With the synthesis of [(Cp*)_2_Sm^III^)_4_P_8_] (Cp* = C_5_Me_5_) by reducing P_4_ with the solvent-free [(Cp*)_2_Sm^II^], our group successfully isolated the first molecular polyphosphide of the rare earth elements in 2009 (Fig. 1a).^15^ This realgar-type structural motif of the [P_8_]^4−^ Zintl anion was also observed shortly after *via* a scandium arene complex and Fe^I^-mediated P_4_ activation.^16,17^ In the field of actinide chemistry, P_4_ is known to be activated by low-valent uranium and thorium species.^18–23^ Other molecular 4f-element compounds bearing a polyphosphide Zintl anion were synthesized either by reduction of [Cp*Fe(P_5_)] with different classical and non-classical divalent lanthanide precursors, or by reduction of [(Cp′′′Co)_2_(P_2_)_2_] (Cp′′′ = 1,2,4-^*t*^Bu_3_C_5_H_2_) with different derivatives of samarocene, which gave access to a variety of mixed d/f-element polyphosphides.^24–29^ It should be noted that the latter compounds discussed here are preceded by the activation of white phosphorus with a transition metal compound. Direct activation of P_4_ with 4f-elements is still rather rare. It is mainly based on SET (single electron transfer) processes of classical divalent Ln^II^-compounds, which either led to the formation of [P_8_]^4−^ (Fig. 1a)^15^ or anti-bimetallic structural motifs with a [P_4_]^2−^ Zintl anion (Fig. 1b),^30^ or oxidative substitution with Ln-arene complexes (Ln = La, Yb, Lu) to form 4f-element polyphosphides bearing a [P_7_]^3−^ Zintl anion.^16,31,32^ The structural motif of a *cyclo*-[P_3_]^3−^ polyphosphide, common for transition metal chemistry,^1,33–35^ is rarely encountered in rare earth compounds. The only reported example is the anti-bimetallic complex K[{(L)Y(thf)}_2_(μ_3_-η^3^:η^3^:η^2^-P_3_)] (L^2−^ = *N*,*N*′-2,6-diisopropylphenyl-1,4-diazabutadiene), synthesised by the alkyl migration of an organosubstituted *cyclo*-P_4_R_2_ precursor. Thereby, the central [P_3_]^3−^ Zintl anion binds at two Y^III^ cations in a bridging coordination mode (Fig. 1c).^36^ In addition, there are two examples, in which a *cyclo*-P_3_ unit, which is part of a larger ligand scaffold, coordinates to a lanthanide atom. The first example, a bicyclo[4.1.0]triphosphaheptanide generated by [3 + 1] fragmentation of white phosphorus with a Lu-metallacyclopentadiene binds to the metal atom.^37^ The second structural example is the 4d/4f hexaphosphide [(Cp*_2_Ln^III^)_2_P_6_(Cp*Mo(CO)_2_)_2_] (Ln = Sm, Yb). Here, the P_6_ unit, which consists of two P–P-bridged *cyclo*-P_3_ rings, coordinates to the lanthanide ions.^29^ Besides structural aspects, the magnetic properties of such compounds, in particular of multinuclear Ln compounds bridged by Zintl anions, may provide new insights in the field of SMMs (single molecule magnets).^38,39^

Recently, organo-lanthanide compounds bridged by a [Bi_6_]^6−^ Zintl anion, which promoted strong ferromagnetic interactions between lanthanides, were reported.^40^

Herein, we compare SET reduction of white phosphorus induced by classical divalent Ln^II^-compounds with a multi-electron reduction induced by the *in situ* reaction of trivalent lanthanide complexes with potassium. As a result, new anti-bimetallic complexes of organo-lanthanide polyphosphides, which were synthesized by direct conversion of white phosphorus using di- and trivalent lanthanide compounds, were obtained. For this purpose, we stabilized the complexes by using the bulky NON ligand (NON = 4,5-bis(2,6-diisopropylphenyl-amino)-2,7-di-*tert*-butyl-9,9-dimethylxanthene)), which was recently employed for stabilizing metal centres in low oxidation states.^41–43^ Within our studies, we showcase the reduction of a polyphosphide within the coordination sphere of a lanthanide complex, which so far is unprecedented.

## Results and discussion

### Synthesis and structural characterization

**Scheme 1:**
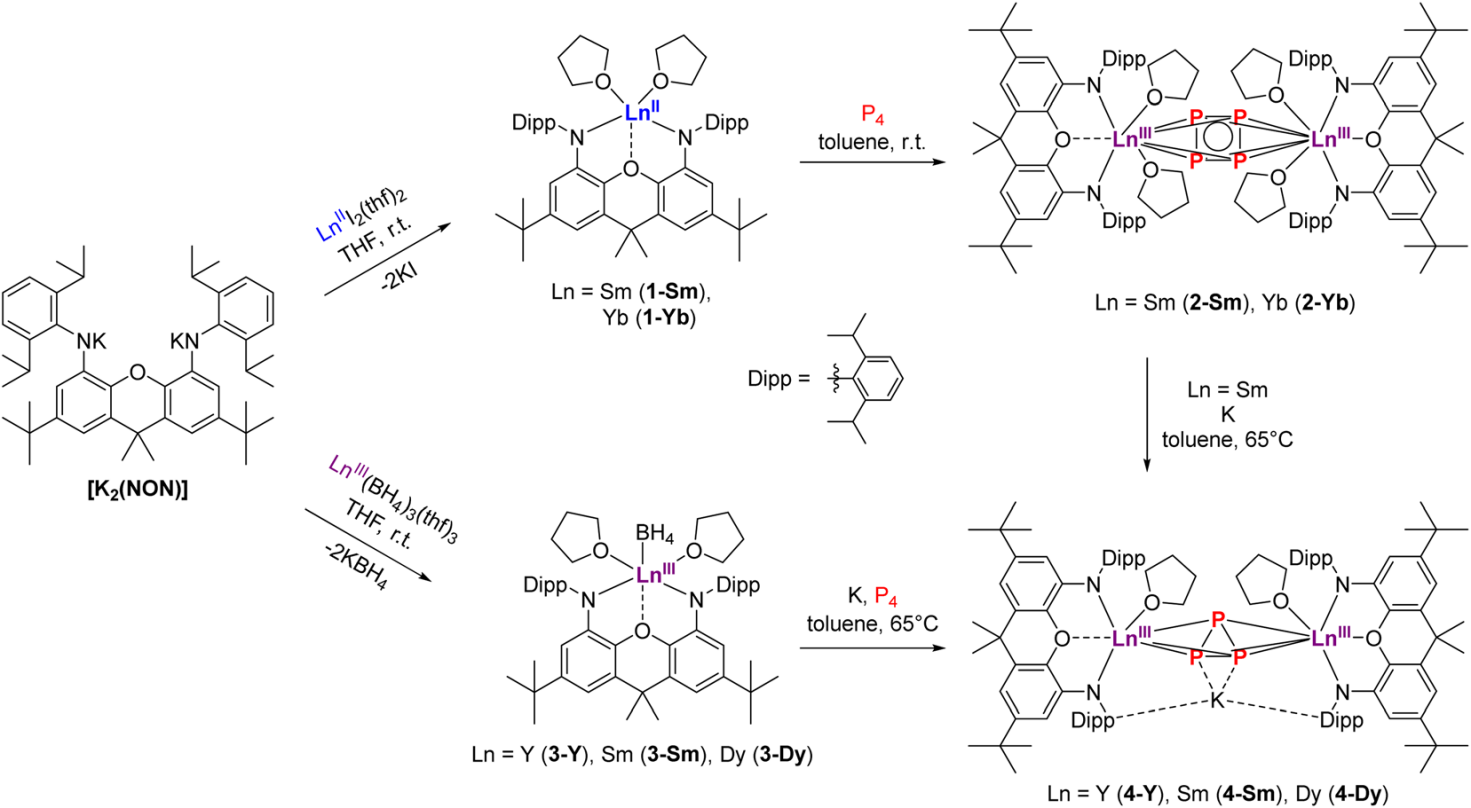
Synthesis of [(NON)Ln^II^(thf)_2_] (1-Ln) and [(NON)Ln^III^(BH_4_)(thf)_2_] (3-Ln) as precursors for P_4_ activation to synthesize the anti-bimetallic *cyclo*-P_4_ (2-Ln) and *cyclo*-P_3_ complexes (4-Ln).

**Fig. 2:**
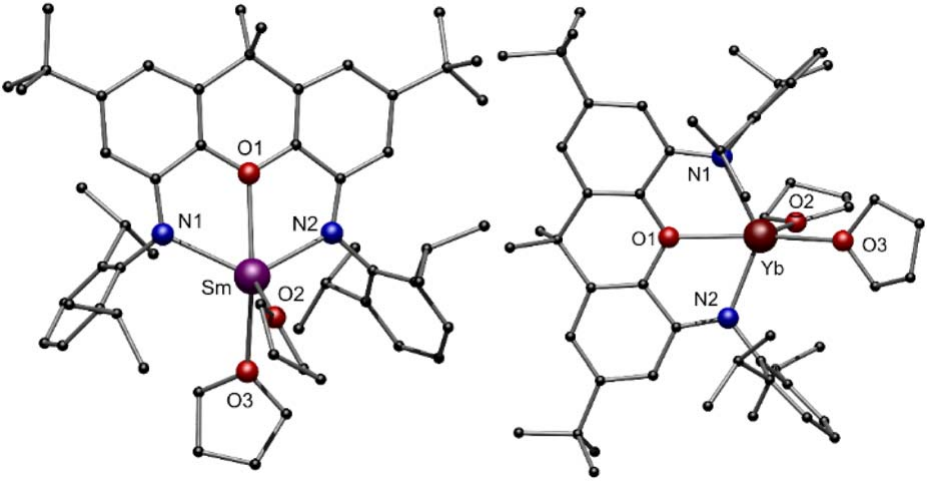
Molecular structure of compounds 1-Sm (left) and 1-Yb (right) in the solid state. Hydrogen atoms are omitted for better clarity. Colour code: Sm (purple), Yb (dark red), N (blue), O (red), C (black).

To realize the synthesis of new lanthanide polyphosphides, we initially adopted an established synthetic route in which classical divalent lanthanide compounds act as SET reagents in the activation process of P_4_. For this purpose, the divalent lanthanide complexes [(NON)Ln^II^(thf)_2_] (Ln = Sm, Yb) (1-Ln) were first synthesised *via* the salt elimination reaction of [K_2_(NON)] and the corresponding diiodides [Ln^II^I_2_(thf)_2_] (Ln = Sm, Yb) (Scheme 1, top). Crystals suitable for X-ray diffraction analysis could be obtained in both cases by recrystallization of the crude products from a hot *n*-heptane solution. Thereby, compound 1-Sm was obtained as large black crystals, while compound 1-Yb appeared as red crystals, both in yields above 60%. Complex 1-Sm crystallizes in the monoclinic space group *P*2_1_, while compound 1-Yb crystallizes in the orthorhombic space group *Pbca* with one molecule each in the asymmetric unit. Both compounds exhibit the same structural motif in the solid state (Fig. 2). The central Ln^II^ cations are coordinated by the two amido functions of the NON ligand and by the oxygen of the xanthene backbone. Two molecules of THF saturate the coordination sphere of the lanthanide. The Sm–N bond lengths in 1-Sm (Sm–N1 2.418(7) Å, Sm–N2 2.433(7) Å) are slightly shorter compared to previously reported divalent (bis)amido Sm^II^-compounds.^44–46^ Similarly, the Sm–O bond lengths are comparable to organometallic Sm^II^-compounds known from the literature (Sm–O1 2.554(5) Å, Sm–O2 2.564(6) Å, Sm–O3 2.526(6) Å).^47^

Due to the smaller ionic radius of Yb^II^ in compound 1-Yb, the Yb–N and Yb–O distances are shortened as expected (Yb–N1 2.344(5) Å, Yb–N2 2.315(5) Å, Yb–O1 2.402(4) Å). This effect is also reflected in the N1–Ln–N2 angles, being 122.8° for 1-Sm and widened for 1-Yb to 132.0°. The NMR spectra of the compound 1-Yb (Fig S1 and S2, ESI†) in C_6_D_6_ at room temperature indicate that no coordinated THF remains on the molecule after drying the material *in vacuo* for several hours. All resonances can be assigned to the xanthene backbone and Dipp-groups (Dipp = diisopropylphenyl) of the ligand, respectively. Due to the paramagnetic behaviour of 1-Sm, no meaningful NMR spectra could be obtained. Elemental analysis of 1-Sm indicates that one THF molecule remains coordinated to the Sm^II^-cation after drying the compound *in vacuo*.

**Fig. 3:**
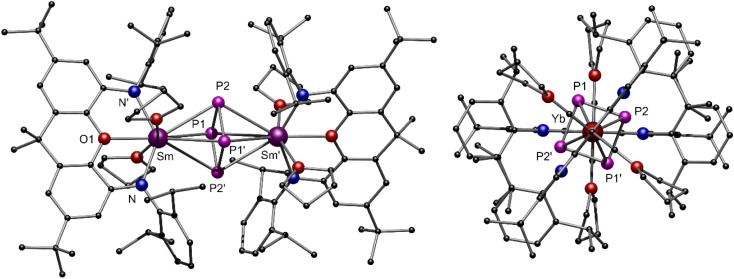
(Left) Molecular structure of compound 2-Sm in the solid state. (Right) Top view of the molecular structure in the solid state of compound 2-Yb to illustrate the staggered arrangement of the xanthene backbones and THF ligands. Hydrogen atoms are omitted for better clarity. Colour code: Sm (purple), Yb (dark red), P (pink), N (blue), O (red), C (black).

After the successful isolation of compounds 1-Ln we investigated the reductive conversion of white phosphorus *via* SET with these compounds (Scheme 1, top). For this purpose, the divalent complexes 1-Ln were stirred together with P_4_ in toluene at room temperature. Thereby deep red solutions were obtained. After filtration of insoluble residues the anti-bimetallic complexes [{(NON)Ln^III^(thf)_2_}_2_(μ-η^4^:η^4^-P_4_)] 2-Ln could be isolated as dark red (2-Sm) and dark orange (2-Yb) crystalline compounds. It should be mentioned here that the crystallization of 2-Sm was carried out from a saturated toluene solution at −10 °C, whereas the solvent had to be changed to THF for crystallization of 2-Yb. This supports the assumption that the coordinating THF remains in 1-Sm after drying *in vacuo*, which is required for the crystallization of 2-Sm, while no coordinating THF molecules persist in 1-Yb. The isostructural compounds crystallize in the orthorhombic space group *Pban*. Similar to the previously reported [{(DippForm)_2_Sm^III^}_2_(μ-η^4^:η^4^-P_4_)] (DippForm = {(2,6-iPr_2_C_6_H_3_)NC(H) = *N*(2,6-iPr_2_C_6_H_3_)}^−^) the *cyclo*-[P_4_]^2−^ Zintl anion coordinates in a bridging η^4^:η^4^ mode between two Ln^III^ centres and exhibits an almost perfect square shape with internal angles of 88.62(9)° and 91.38(9)° for 2-Sm and 88.73(8)° and 91.27(8)° for 2-Yb, respectively (Fig. 3). Thereby the P–P distance of 2.15 Å in 2-Ln as well as the Sm–P distances of 3.0727(8) Å and 3.0908(10) Å are in the range of reported aromatic [P_4_]^2−^ Zintl anions in the coordination sphere of two Sm^III^-moieties.^30^ Corresponding Yb–P distances are slightly shorter (Yb–P1 3.0192(8) Å, Yb–P2 3.0021(8) Å) and in the range of the reported values.^32^ Compared to the divalent precursors 1-Ln, the Ln–N distances (Sm–N 2.360(4) Å, Yb–N 2.290(3) Å) are moderately shorter and decrease with the ionic radius of the Ln^III^ cation as well. To illustrate the staggered arrangement of the xanthene backbones and THF ligands in 2-Ln, the top view of 2-Yb is displayed on the right side of Fig. 3. For both compounds the torsion angle of the xanthene backbones is around 53° (see Fig. S27 and S29, ESI†). Compound 2-Sm is only the second example for P_4_ activation which led to the formation of a 6-π-aromatic [P_4_]^2−^ ring in the coordination sphere of two Sm^III^ cations and was entirely unknown for Yb^III^ compounds so far. NMR spectra of 2-Ln were recorded in C_6_D_6_ at room temperature. Due to the paramagnetic behaviour of complexes 2-Ln no meaningful ^1^H and ^13^C NMR spectra were obtained. In the ^31^P{^1^H} NMR spectra, however, one singlet was found in each case. The singlet at *δ* = 479.5 ppm for 2-Sm could be assigned to the four chemically equivalent phosphorus atoms of the *cyclo*-P_4_ moiety. Thus, the chemical shift is in a similar range to that of the previously reported [{(DippForm)_2_Sm}_2_(μ-η^4^:η^4^-P_4_)].^30^

For compound 2-Yb, the ^31^P{^1^H} NMR signal of the four phosphorus atoms is slightly shifted to higher resonances and can be detected as a singlet at *δ* = 382.4 ppm.

Motivated by these results, we aimed to synthesize further Ln^III^ polyphosphides beyond Sm and Yb in order to investigate different reducing processes and to have access to a broader range of rare-earth elements for studying their physical properties. In particular, we focused on the synthesis of a dinuclear polyphosphide-bridged Dy^III^ compound, which could potentially exhibit SMM behaviour. Although [DyI_2_(dme)_3_] is known,^48^ it has proven too reactive for being a suitable precursor in organometallic chemistry. Therefore, a synthetic route starting from a robust and isolable Ln^III^ species was chosen, which subsequently should be reduced in the presence of P_4_ to form new molecular Ln^III^ polyphosphides. The first step was achieved by a salt elimination reaction between [K_2_(NON)] and the respective [Ln^III^(BH_4_)_3_(thf)_3_] (Ln = Y, Sm, Dy) (Scheme 1, bottom). The corresponding compounds [(NON)Ln^III^BH_4_(thf)_2_] 3-Ln could be isolated by recrystallization of crude materials from a hot *n*-heptane solution as colourless (3-Y), red (3-Sm) and light green (3-Dy) crystals in high yields. The isostructural compounds crystallize in the monoclinic space group *P*2_1_/*c* with one molecule in the asymmetric unit.

**Fig. 4:**
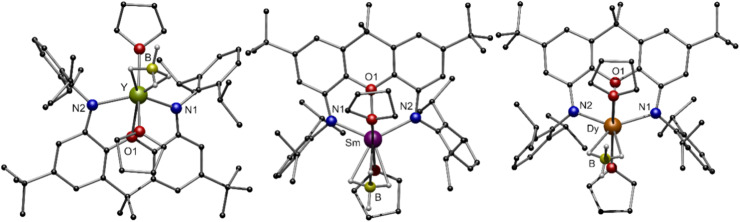
Molecular structure of compounds 3-Ln (Ln = Y, Sm, Dy) in the solid state. Hydrogen atoms, except the freely refined hydridic ones, are omitted for clarity. Colour code: Y (green), Sm (purple), Dy (orange), B (yellow), N (blue), O (red), C (black), H (grey).

The molecular structures in the solid state of [(NON)Ln^III^BH_4_(thf)_2_] 3-Ln are shown in Fig. 4. As expected, the central Ln^III^ cation is coordinated by two amido functions and the oxygen atom of the NON ligand. Additionally, two molecules THF saturate the coordination sphere of the metal centre. Solely the coordination mode of the [BH_4_]^−^ moiety, which was determined by free refinement of the hydrogen atoms, differs in 3-Ln. While the [BH_4_]^−^ ligand in 3-Y and 3-Dy coordinates in an η^2^-H_2_BH_2_ mode, an η^3^-H_3_BH coordination mode is observed for 3-Sm. The dependence of the coordination mode of borohydrides on the ionic radii and steric demand of the applied ligands is well known in lanthanide chemistry.^49,50^ In the case of 3-Ln, the hapticity of [BH_4_]^−^ decreases with the ionic radius of the Ln^III^ cation with a constant steric demand of the ligand. The respective Ln–B distances are between 2.671(3) Å in 3-Sm and 2.717(3) Å in 3-Y. In the FT-IR spectra of compounds 3-Ln, different absorption patterns between 2400 cm^−1^ and 2100 cm^−1^ are observed for the different coordination modes of the [BH_4_]^−^ moiety.

In the ^1^H{^11^B} NMR spectrum of 3-Y the singlet at *δ* = 1.13 ppm can be assigned to the four hydridic hydrogens of the [BH_4_]^−^ moiety whereas no resonances for the hydrogen atoms of the borohydride can be found in the ^1^H NMR spectrum. All other resonances can be assigned to the NON ligand and one remaining coordinated THF molecule. The ^11^B NMR spectra show one broad signal at *δ* = −24.2 ppm for 3-Y and at *δ* = −38.5 ppm for 3-Sm, while remaining silent for 3-Dy due to the strong paramagnetic behaviour.

**Fig. 5:**
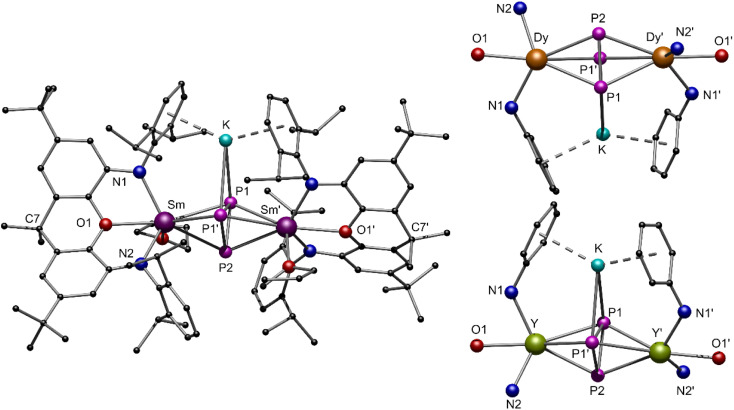
(Left) Molecular structure of compound 4-Sm in the solid state. (Top/bottom right) Stylized illustration of the central structural motifs of isomorphic compounds 4-Y and 4-Dy. Hydrogen atoms and one molecule of toluene are omitted for better clarity. Colour code: Sm (purple), Dy (orange), Y (green) P (pink), N (blue), O (red), K (turquoise), C (black).

To reach the aim for new Ln polyphosphides beyond Sm and Yb, we applied the trivalent compounds 3-Ln in one-pot reactions with an excess of potassium and P_4_ at elevated temperatures in toluene (Scheme 1, bottom). After filtration, [K{(NON)Ln(thf)}_2_(μ_3_-η^3^:η^3^:η^2^-P_3_)] 4-Ln could be isolated as colourless (4-Y), red (4-Sm) and yellow (4-Dy) crystals at −10 °C from a saturated toluene solution. X-ray diffraction analysis revealed a bridging *cyclo*-[P_3_]^3−^ unit in a μ-η^3^:η^3^ coordination mode as the central building block, which displays an almost perfect triangular shape with internal angles between 58.97(4)° and 62.04(5)° for P1–P2–P1′ and P1–P1′–P2, respectively (Fig. 5). The formation of 4-Ln shows that the multi-electron reduction applied here results in a different product. The P–P bond lengths within 4-Ln range from 2.160(2) Å to 2.238(2) Å, consistent with literature values of bridging *cyclo*-[P_3_]^3−^ polyphosphides.^34,36^ Here, the P1–P1′ bond is always slightly elongated compared to the P1–P2 bond due to the coordination of P1 to the potassium ion (K–P1 ∼ 3.18 Å). The Ln–Ct_P3_ bond distances (Ct_P3_ = centroid of the *cyclo*-[P_3_] moiety) in 4-Ln decrease with the radius of the respective rare earth ion. This in turn affects the K–Ct_phenyl_ distances, which thus also follow this trend (4-Sm: K–Ct_phenyl_ 2.8112(6) Å to 4-Dy: K–Ct_phenyl_ 2.7727(4) Å). The coordination of the potassium ion by the two phenyl rings of the Dipp-moieties further leads to the folding of the originally planar xanthene backbone along the Ln–O1–C7 axis by about 35° (see Fig. S34, S36 and S38, ESI†). Using the specific example of 4-Sm, the Sm–Ct_P3_ distance of 2.6030(7) Å can be directly compared with the Sm–Ct_P4_ distance 2-Sm. The latter is slightly longer with 2.6787(7) Å, which is due to the higher charge density in the *cyclo*-[P_3_]^3−^ compared to the *cyclo*-[P_4_]^2−^ polyanion in an otherwise similar chemical environment. The resulting stronger electrostatic interactions lead to a shorter Sm–Ct_P3_ distance.

**Fig. 6:**
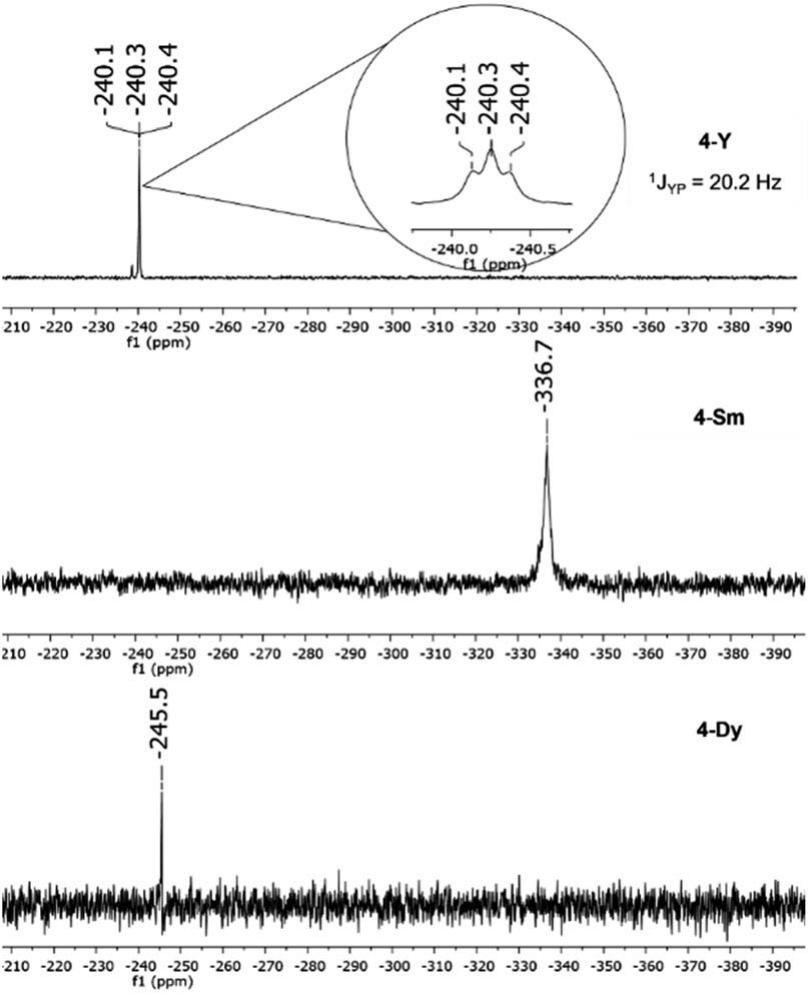
^31^P{^1^H} NMR spectra of [K{(NON)Ln^III^(thf)}_2_(μ_3_-η^3^:η^3^:η^2^-P_3_)] (4-Ln) in thf-d_8_ at 25 °C.

The ^31^P{^1^H} NMR spectra of compounds 4-Dy and 4-Sm show one singlet each for the *cyclo*-[P_3_]^3−^ moiety at *δ* = −245.5 ppm (4-Dy) and *δ* = −336.7 ppm (4-Sm) (Fig. 6). The resonance of the *cyclo*-[P_3_]^3−^ compound 4-Sm is thus considerably shifted to higher field frequencies compared to the corresponding *cyclo*-[P_4_]^2−^ compound 2-Sm. For compound 4-Y a triplet is seen in the ^31^P{^1^H} NMR spectrum at *δ* = −240.3 ppm with a coupling constant of ^1^*J*_YP_ = 20.2 Hz (Fig. 6), which is slightly higher than that of the reported complex K[{(L)Y(thf)}_2_(μ_3_-η^3^:η^3^:η^2^-P_3_)] (^1^*J*_YP_ = 16.3 Hz).^36^

To get further insights in the formation of the [P_3_]^3−^ Zintl anion in 4-Ln, we first investigated the reduction of 2-Sm with potassium in the NMR scale (Scheme 1, right). After a few hours at 65 °C, the ^31^P NMR resonance at 479.5 ppm in C_6_D_6_, which is assigned to the [P_4_]^2−^ unit of 2-Sm (ESI, Fig. S3†), vanished and a new resonance at −320.8 ppm appeared. After removing C_6_D_6_*in vacuo* and resolving the residue in thf-d_8_, the resonance was detected at −336.7 ppm, which is consistent with the chemical shift of the isolated compound 4-Sm from the one pot reaction of 3-Sm with potassium and P_4_ (Fig. 6). This was further confirmed by the reaction of 2-Sm with potassium in a preparative scale with subsequent crystallisation of 4-Sm. In the ^31^P{^1^H} NMR spectrum no further resonances, which could be assigned to possible by-products, were detected. Hence, the reactivity of 2-Sm with potassium clearly shows that the [P_4_]^2−^ can be reduced to a [P_3_]^3−^ Zintl anion in the coordination sphere of two Sm^III^ cations. Therefore, we anticipate that during the formation of 4-Ln, P_4_ is reduced to a [P_4_]^2−^ moiety first and subsequently reduced to a [P_3_]^3−^ Zintl anion. However, isolation of the [P_4_]^2−^ species from the one-pot reaction of compounds 3-Ln was not possible even with variation of stoichiometry and temperature.

### DFT calculations

To obtain a better insight into the bonding situation of the compounds under discussion, we performed quantum chemical DFT calculations on the molecules 2-Sm and 4-Sm (symmetries *S*_4_ (2-Sm) and *C*_2_ (4-Sm), def-SV(P) for all atoms, ecp for Sm^3+^,^51^ RI-BP86,^52–54^ program system TURBOMOLE).^55^ The existence of real local minima was confirmed by frequency calculations using the aoforce module. The calculated structural data are in good agreement with the experimentally determined data. As in other lanthanide complexes,^15^ we find that the bonding from the rare earth cations to polyphosphide ligands can be described primarily as ionic bonds. For this reason, the anionic *cyclo*-[P_3_]^3−^ and *cyclo*-[P_4_]^2−^ ligands were also calculated as purely ionic model compounds K_3_P_3_ and K_2_P_4_ (point symmetries *C*_1_ and *D*_4h_) and compared with the situation in these complexes. The investigation of the structural data is supported by shared electron numbers (SEN) from population analyses based on occupation numbers according to the method of Ahlrichs and Heizmann that serve as measures for covalent bonding.^56,57^ For 2-Sm, the P–P bond distance and the SEN are calculated to be 2.202 Å and 1.31, respectively. The values obtained for P_4_^2−^ in K_2_P_4_ are in good agreement with this finding (bond distance 2.200 Å, SEN 1.27). To evaluate the aromaticity, multi-center SEN(P_4_) of both particles were determined; the results support slight aromatic bonding (2-Sm: SEN(P_4_) 0.1; K_2_P_4_: SEN(P_4_) 0.08). Moreover, this behavior is supported by the NICS values of 2-Sm (NICS(0) +3.2, NICS(1) −4.5 ppm). For 4-Sm the P–P bond distances were calculated to be 2.244 and 2.292 Å, the SEN (P–P) were determined to be 1.20 and 1.12. The principal setup of the model compound K_3_P_3_ is comparable to that of 4-Sm: the P_3_ unit is surrounded by the strongly positive charged potassium cations in a complicated three dimensional manner due to their electrostatic repulsions. Still, in K_3_P_3_ the structural parameters nicely reflect those in 4-Sm: P–P bond distances are calculated to be 2.213 and 2.306 Å, the SEN (P–P) are determined to be 1.23 and 1.09. Comparison of the P–P bond distances and SEN in 2-Sm with those of 4-Sm furthermore supports the idea of a slight aromatic character in the former system. To sum up, in both compounds 2-Sm and 4-Sm the *cyclo*-[P_3_]^3−^ and *cyclo*-[P_4_]^2−^ units are best described as polyphosphide anions stabilized by counterions through mainly polar bonding contributions.

### Magnetic measurements of 4-Dy

The highly anisotropic nature of trivalent lanthanide ions caused by strong spin–orbit coupling has led to a plethora of Ln^III^ coordination compounds showing SMM behaviour.^58,59^ The rise of single molecular magnetism over the past decades is based on the promising applicability of SMMs in different quantum technological fields.^60–62^ Especially Dy^III^ due to its strong magnetic moment (*J* = 15/2) and it being a Kramers ion is often reported to exhibit slow magnetic relaxation. We, therefore, investigated the magnetic properties of 4-Dy by means of SQUID magnetometry alongside *ab initio* CASSCF calculations. Full details of the methodology can be found in the ESI.†

**Fig. 7:**
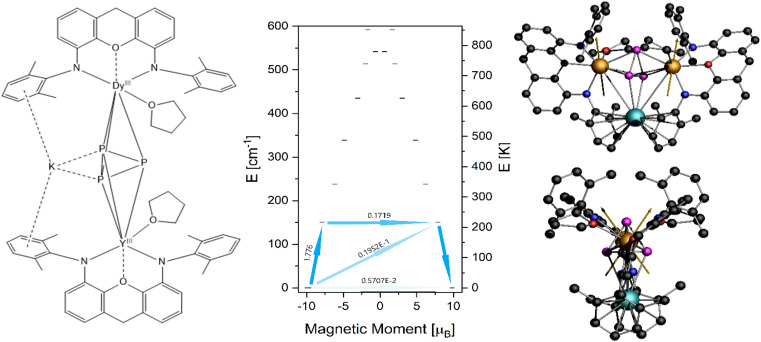
(Left) Structural scheme of 4-Dy*, (middle) energy spectrum obtained for 4-Dy* and transition probabilities within the low lying Kramers doublets, (right) orientation of the main magnetic axis found for 4-Dy*, the orientation on the *Y*-position is deduced by the molecular symmetry.

Our first attempt at calculating the molecular properties was performed following a common approach, replacing one Dy^III^ with a diamagnetic Y^III^ and keeping the molecular structure as it is obtained from XRD methods. For 4-Dy, however, the CASSCF procedure did not converge and we therefore opted to carry out the calculations based on a fictional Dy–Y compound (4-Dy*) in which we replaced the peripheral methyl and *tert*-butyl groups on the NON ligands with hydrogen atoms, as well as the iso-propyl groups on the DIPP groups with methyl (Fig. 7, left). The results of the single ion calculation showed a strongly axial ground state characterised by *g*_*x*_ = 0.01, *g*_*y*_ = 0.02 and *g*_*z*_ = 19.64 with the wave function being mainly composed of *m*_*J*_ = 15/2, but showing some admixing of *m*_*J*_ = 11/2 (Table S12, ESI†). This mixing suggests that quantum tunnelling within the ground state is possible, which is also confirmed by the relatively high transition probability of the ground state (Fig. 7, middle). The first excited Kramers doublet is found at 151.0 cm^−1^ (217.25 K) with *g*-values *g*_*x*_ = 0.36, *g*_*y*_ = 0.67 and *g*_*z*_ = 15.76. The main magnetic axis is only tilted 1.79° in respect to that of the ground state (Fig. 7, right), which can be an indicator that magnetic relaxation might occur *via* higher excited states. However, the state's wave function shows strong admixing (19%) of *m*_*J*_ = 9/2, which lets us assume that relaxation will mainly occur through this state (Table S13, ESI†).

**Fig. 8:**
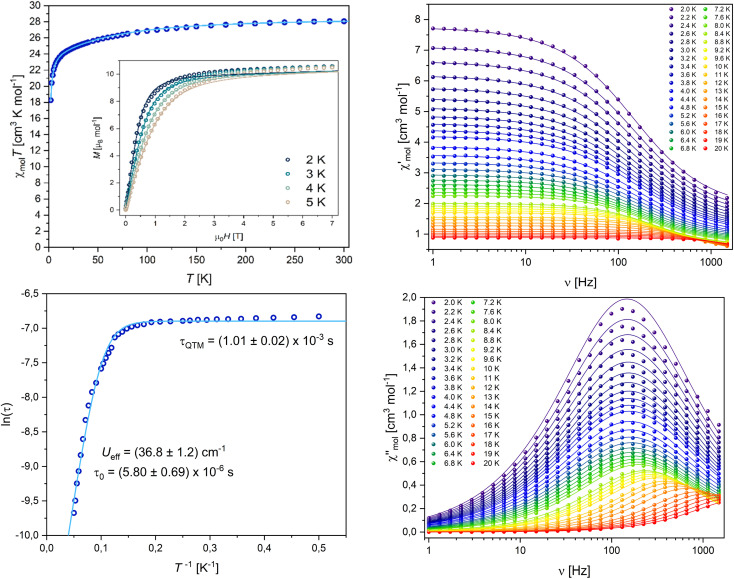
(Top left) Temperature product of the molar susceptibility *vs.* temperature and molar magnetisation *vs.* field of 4-Dy, solid lines are best fits obtained with PHI, experimental data points are scaled to allow for simulation.^63^ (Bottom left) Arrhenius plot; (top and bottom right) in-phase and out-of-phase susceptibilities *vs.* frequency of 4-Dy, solid lines are the results of a simultaneous fit to a generalised Debye model.

The static magnetic behaviour of 4-Dy was tested on a sample in a flame sealed NMR tube upon cooling from 300 K to 2 K under an applied field of 0.1 T and through measurements of the molar magnetisation up to 7 T at temperatures 2, 3, 4 and 5 K. The behaviour of the susceptibility product *χ*_mol_*T* against temperature shows a slow decrease followed by a strong drop around 10 K (Fig. 8), suggesting antiferromagnetic coupling of the two Dy^III^ ions. Note that the absolute values of *χ*_mol_*T* and the magnetisation given in Fig. 8 have been scaled up in order to allow simulations, *vide infra*. The observed room temperature value of *χ*_mol_*T* was 20.16 cm^3^ K mol^−1^, which is only about 71% of the expected value for two uncoupled Dy^III^ ions of 28.34 cm^3^ K mol^−1^. We believe that this error is introduced through sample preparation, as we have experienced it before and the handling of very small amounts of sample inside a glove box can lead to big percentage errors very quickly.^64^ However, in further discussion of dynamic behaviour and simulation of the data, introducing a correction factor as we did does not influence the results. Employing the crystal field parameters obtained from the single ion CASSCF calculation (Table S13†) rotated onto a common reference frame, we performed a simultaneous fit of *χ*_mol_*T*(*T*) and *M*(*H*) using a single isotropic exchange. An almost perfect fit for *χ*_mol_*T*(*T*) and good agreement around lower fields for *M*(*H*) was obtained with *J*_iso_ = −2.96 × 10^−2^ cm^−1^ (employing a -2*J* formalism), confirming our initial assumption of antiferromagnetic interactions.

The dynamic behaviour of 4-Dy was tested at low temperatures under application of an oscillating magnetic field (3.5 Oe). A peak in the frequency-dependent out-of-phase susceptibility was observed around 200 Hz at 2 K (Fig. 8, right). Upon the application of additional DC fields, the signal vanished quickly. Simultaneous fits of the in-phase and out-of-phase susceptibilities to a generalised Debye model (all fit parameters can be found in Table S14, ESI†) and subsequent Arrhenius analysis of the relaxation times gave an effective energy barrier *U*_eff_ = 36.8 cm^−1^ and relaxation times *τ*_0_ = 5.80 × 10^−6^ s, *τ*_QTM_ = 1.01 × 10^−3^ s. Note that we performed the Arrhenius fit employing eqn (1), leaving out the commonly employed CT^*n*^ Raman-term, as we already obtained a very good fit employing only Orbach and QTM processes.

1 *τ*^−1^ = *τ*_0_−1 exp(−*U*_eff_/*k*_B_*T*) + *τ*_QTM_−1

We estimated the blocking temperature *T*_B_ as the temperature at which *τ* = 100 s and found *T*_B_ = 3.17 K. This is in good agreement with hysteresis loops that we recorded, in which the hysteresis is slightly open at 2 K but essentially closed already at 3 K (Fig. S42, ESI†). The experimental energy barrier of 36.8 cm^−1^ seems extremely low, given the expected relaxation pathway through the first excited state at 151 cm^−1^. However, it has been shown before that single ion CASSCF calculations often fall short when predicting the relaxation barrier of polynuclear compounds. Dey and Rajaraman have proposed an empirical model to estimate the barrier height of Dy_2_ SMMs (2):^65^2
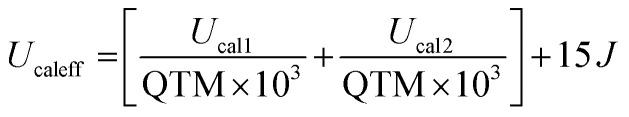
where *U*_cal1_ and *U*_cal2_ are the energies of the excited Kramers doublet through which relaxation occurs for Dy1 and Dy2, QTM is the transition probability within the underlying doublet and *J* is the exchange coupling. Employing *U*_cal1_ = *U*_cal2_ = 151 cm^−1^, QTM = 0.5707 × 10^−2^ and *J* = 2 × *J*_iso_ = 5.92 × 10^−2^ cm^−1^, we found *U*_caleff_ = 52.0 cm^−1^. Acknowledging the fact that we have performed the *ab initio* calculations on a model molecule 4-Dy*, we believe that the estimated barrier is in rather good agreement with what we have found experimentally.

The most impressive relaxation barriers to this day have been achieved in Dy^III^ complexes bearing aromatic Cp derived ligands due to their axial ligand fields being beneficial in stabilising the magnetic moment.^66–68^ The [P_4_]^2−^ and [P_3_]^3−^ Zintl ions might give similar results in the future when being paired with other ligands. We believe the rather poor SMM behavior is a consequence of the NON-ligand producing an unfavorable ligand field for Dy^III^ as well as causing misalignment of the easy axes. As this is the first report of a polyphosphide containing SMM we are excited to see what other possibilities for SMMs these structural motifs will bring in the future.

## Conclusions

In summary, we have demonstrated that the activation of white phosphorus, on the one hand, with classical divalent lanthanide compounds 1-Ln (Ln = Sm, Yb) *via* SET, and, on the other hand, with the trivalent lanthanide borohydride compounds 3-Ln (Ln = Y, Sm, Dy) in the presence of potassium *via* a multi-electron reduction leads selectively to different products. As products, organo-lanthanide polyphosphides with an aromatic *cyclo*-[P_4_]^2−^ (2-Ln, Ln = Sm, Yb) as well as a *cyclo*-[P_3_]^3−^ (4-Ln, Ln = Y, Sm, Dy) Zintl anion were isolated and characterized. The synthetic route of a one-pot reaction allowed the isolation of f-element polyphosphides beyond utilization of relatively stable, classical divalent Sm and Yb compounds. This led to the characterization of the first polyphosphide bridged dinuclear Dy^III^ SMM (4-Dy). Furthermore, we have shown the reduction of the *cyclo*-[P_4_]^2−^ moiety to form a *cyclo*-[P_3_]^3−^ Zintl anion in the coordination sphere of two lanthanides by reacting 2-Sm with potassium.

## Data availability

All synthetic protocols, spectroscopic data, supplementary figures and tables, magnetic data, data from quantum chemical calculations and detailed crystallographic information can be found in the ESI.† Crystallographic data are available *via* the Cambridge Crystallographic Data Centre (CCDC): 2224069–2224077, and 2235392.

## Author contributions

AH, CU, and UCR synthesized and analyzed all compounds with support from LM. LM conducted X-ray experiments. SS and MR conducted and interpreted magnetic measurements and carried out the *ab initio* CASSCF calculations and interpreted the results. RK performed and analyzed quantum chemical calculations. PWR proposed the idea, supervised the work, and interpreted the results. All authors contributed to the preparation of the manuscript.

## Conflicts of interest

The authors declare no conflict of interest.

## Supplementary Material

SC-014-D2SC06730G-s001

SC-014-D2SC06730G-s002

SC-014-D2SC06730G-s003
