# Supplementary material for: Molecular cyclo-P3 complexes of the rare-earth elements via a one-pot reaction and selective reduction
Source: Chem Sci. 2023 Feb 3;14(8):2149–58. doi: 10.1039/d2sc06730g (PMC9945584; doi:10.1039/d2sc06730g)
Supplement: SC-014-D2SC06730G-s002 [file SC-014-D2SC06730G-s002.pdf]

Electronic Supplementary Material (ESI) for Chemical Science.
This journal is © The Royal Society of Chemistry 2023

Supporting Information

Molecular cyclo-P_3 complexes of the Rare-Earth Elements via one pot reaction and selective reduction

Adrian Hauser[a], Luca Münzfeld[a], Sören Schlittenhardt[b], Ralf Köppe[a], Cedric Uhlmann[a], Ulf-Christian Rauska[a] Mario Ruben[b,c,d] and Peter W. Roesky*[a]

[a] Institute of Inorganic Chemistry, Karlsruhe Institute of Technology (KIT), Engesserstraße 15, D-76131 Karlsruhe, Germany. E-mail: roesky@kit.edu

[b] Institute of Nanotechnology, Karlsruhe Institute of Technology (KIT), Hermann-von-Helmholtz-Platz 1, D-76344 Eggenstein-Leopoldshafen, Germany.

[c] Centre Européen de Science Quantique (CESQ); Institut de Science et d'Ingénierie Supramoléculaires (ISIS, UMR 7006), CNRS-Université de Strasbourg, 8 allée Gaspard Monge BP 70028 67083 Strasbourg Cedex, France

[d] Institute of Quantum Materials and Technologies (IQMT), Karlsruhe Institute of Technology, Hermann-von-Helmholtz-Platz 1, 76344 Eggenstein-Leopoldshafen, Germany.

1. General Methods: S3
2. Synthesis S4
3. NMR Spectra S9
4. IR Spectra S16
5. X-ray Crystallographic Studies S21
6. Magnetism S41
7. Quantum Chemical Calculations S45
8. References S46

1. General Methods:

All manipulations of air-sensitive materials were performed under rigorous exclusion of oxygen and moisture in flame-dried Schlenk-type glassware either on a dual manifold Schlenk line, interfaced to a high vacuum pump (10^{-3} mbar), or in an argon-filled MBraun glove box. Hydrocarbon solvents were pre-dried using an MBraun solvent purification system (SPS-800), degassed and stored *in vacuo* over $LiAlH_4$. Tetrahydrofuran was additionally distilled under nitrogen over potassium before storage *in vacuo* over $LiAlH_4$. THF was dried over K and degassed by freeze-pump-thaw cycles. Elemental analyses were carried out with an Elementar Vario MICRO Cube. NMR spectra were recorded on Bruker spectrometers (Avance III 300 MHz, Avance Neo 400 MHz, or Avance III 400 MHz). Chemical shifts are referenced internally using signals of the residual protio solvent (^{1}H, $^{1}H\{^{11}B\}$) or the solvent ($^{13}C\{^{1}H\}$) and are reported relative to tetramethylsilane (^{1}H, $^{13}C\{^{1}H\}$), phosphoric acid (85%) ($^{31}P\{^{1}H\}$) or BF_3-Et_2O (15%) in $CDCl_3$ (^{11}B). All NMR spectra were measured at 298 K. The multiplicity of the signals is indicated as s = singlet, d = doublet, t = triplet, sept. = septet, m = multiplet, and br = broad. IR spectra were obtained on a Bruker Tensor 37 spectrometer equipped with a room temperature DLaTGS detector and a diamond ATR (attenuated total reflection) unit. In terms of their intensity, the signals were classified into different categories (vs = very strong, s = strong, m = medium, w = weak).

$[Ln(BH_4)_3(thf)_3]$[1] (Ln = Y, Sm, Dy), $[LnI_2(thf)_2]$[2] (Ln = Sm, Yb), K_2NON[3, 4] (NON = 4,5-bis(2,6-diisopropylphenyl-amino)-2,7-di-tert-butyl-9,9-dimethylxanthene) were prepared according to literature procedures. All other chemicals were obtained from commercial sources and used without further purification.

Note: To ensure the best possible purity and reliability of all compounds, only crystalline material was isolated. Hence all yields and analytics refer to isolated crystalline samples, whereas yields are generally lower compared to bulk samples.

Potential Hazards: Potassium may violently react with moisture, water and air, particular attention need to be paid. P_4 is pyrophoric.

2. Synthesis

Synthesis of [(NON)Ln^{II}(thf)$_2$] (1-Ln)

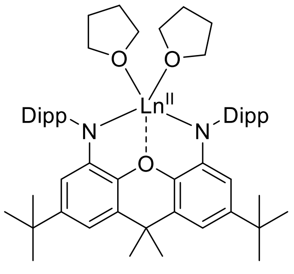

In a general procedure 20 mL of THF was added to a mixture of 500 mg [K_2(NON)] (0.667 mmol, 1.00 eq.) and the corresponding [$Ln^{II}I_2$(thf)$_2$] (0.667 mmol, 1.00 mmol, Ln = Sm: 366 mg, Yb: 381 mg). After stirring the reaction mixture at room temperature for 16 h, all volatiles were removed *in vacuo*. Afterwards, the remaining solid was extracted with 30 mL of hot *n*-heptane and filtered while still hot. The residue was washed with another 20 mL of hot *n*-heptane to increase the yields. Upon cooling the filtrate to room temperature, single crystals suitable for X-ray diffraction could be obtained. The crystalline yield could be increased by reducing the amount of solvent *in vacuo* and storing the filtrate at -20°C. The products were isolated by separating the crystals from the mother liquor as highly crystalline black (Ln = Sm) and orange (Ln = Yb) solids. Elemental analysis indicates removal of one molecule of THF from **1-Sm** upon drying in vacuo. Thus, above depiction of compound **1-Sm** reflects their molecular structure in the solid state as determined by single crystal X-ray diffraction, since the exact structure of the isolated solvate-free compounds was not investigated. For **1-Yb** the ^{1}H NMR spectrum shows no THF resonance after drying the substance *in vacuuo*.

[(NON)Sm^{II}(thf)] (1-Sm): Yield: 400 mg (0.448 mmol), 67 %. **Anal. calcd**. (%) for [$C_{51}H_{70}N_2O_2Sm$] (893.49 g/mol): C: 68.56, H: 7.90, N: 3.14; **found** (%): C: 68.57, H: 7.59, N: 3.04. **IR (ATR):** $\tilde{\nu}$ [cm^{-1}] = 3064 (w), 2960(vs), 2866 (s), 1792 (w), 1698 (w), 1622 (m), 1575 (w), 1517 (m), 1461 (m), 1422 (m), 1429 (m), 1412 (m), 1392 (m), 1359 (w), 1337 (m), 1305 (m), 1249 (w), 1219 (m), 1199 (m), 1140 (w), 1115 (w), 1056 (w), 1030 (w), 1016 (w), 934 (w), 869 (w), 851 (w), 800 (w), 771 (w), 750 (w), 730 (w), 695 (w), 659 (w), 635 (w), 542 (w), 509 (w), 469 (w), 415 (w).

Note: Due to the paramagnetic behavior of the Sm-compound (**1-Sm**) no meaningful NMR spectra were obtained.

[(NON)Yb] (1-Yb): Yield: 370 mg (0.438 mmol), 66 %. **Anal. calcd**. (%) for [$C_{47}H_{62}N_2OYb$] (844.08 g/mol): C: 66.88, H: 7.40, N: 3.32; **found** (%): C: 67.13, H: 7.29, N: 3.11. **^{1}H NMR** (400.30 MHz, C_6D_6): δ [ppm]: 7.32-7.19 (m, 6 H, Dipp-C*H*$_{ar}$), 6.63 (d, $^4J_{HH}$ = 2.2 Hz, 2 H, Xanth-*p*-C*H*), 6.20 (br, 2 H, Xanth-*o*-C*H*), 3.65-3.60 (m, 4 H, Dipp-C*H*(CH$_3$)$_2$), 1.78 (s, 6 H, Xanth-C(C*H*$_3$)$_2$), 1.38 (s, 18 H, Xanth-C(C*H*$_3$)$_3$), 1.35 (d, $^3J_{HH}$ = 6.8 Hz, 12 H, Dipp-CH(C*H*$_3$)$_2$), 1.17 (d, $^3J_{HH}$ = 7.0 Hz, 12 H, Dipp-CH(C*H*$_3$)$_2$). **^{13}C{^{1}H} NMR** (100.67 MHz, C_6D_6): δ [ppm]: 149.0, 147.8, 146.4, 145.4, 129.3, 124.3, 123.8, 122.7 (Ar-*C*), 107.9 (Xanth-*o*-*C*H), 103.5 (Xanth-*p*-*C*H), 35.2 (Xanth-*C*(CH$_3$)$_2$), 35.0 (*C*(CH$_3$)$_3$), 32.2 (C(*C*H$_3$)$_3$), 31.7 (Xanth-C(*C*H$_3$)$_2$), 28.0 (Dipp-*C*H(CH$_3$)$_2$), 25.6 (Dipp-CH(*C*H$_3$)$_2$), 25.2 (Dipp-CH(*C*H$_3$)$_2$). **IR (ATR):** $\tilde{\nu}$ [cm^{-1}] = 3065 (w), 2959 (vs), 2928 (m), 2866 (m), 1623 (m), 1573 (w), 1517 (m), 1461 (m), 1437 (m), 1411 (w), 1391 (w), 1360 (w), 1338 (w), 1305 (w), 1253 (w), 1218 (m), 1179 (w), 1140 (w), 1112 (w), 1056 (w), 1016 (w), 934 (w), 869 (w), 848 (w), 801 (w), 771 (w), 759 (w), 738 (w), 697 (w), 658 (w), 540 (w), 512 (w), 454 (w), 415 (w).

Synthesis of [{(NON)Ln(thf)$_2$}$_2$(μ-η^4:η^4-P_4)] (2)

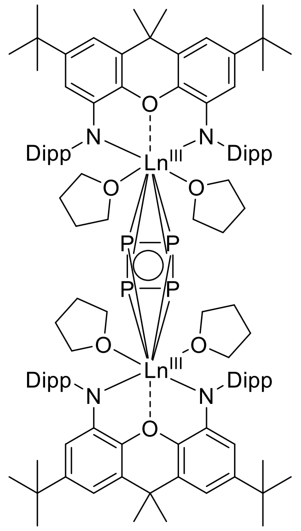

Ln = Sm (**2-Sm**), Yb (**2-Yb**)

In a general procedure, toluene (20 mL) was added to a mixture of 0.220 mmol [(NON)Ln(thf)$_n$] (**1**) (Ln = Sm, n = 1, 197 mg, Yb n = 0, 186 mg, 2.00 eq) and 13.6 mg P_4 (0.110 mmol, 1.00 eq). The reaction mixture was stirred at room temperature for 16 h and turned deep red in both cases. After filtration of insoluble by-products and reducing the solvent *in vacuo* red single crystals suitable for X-ray diffraction of [{(NON)Sm(thf)$_2$}$_2$(μ-η^4:η^4-P_4)] (**2-Sm**) were obtained by storing the toluene solution at -30 °C for several days. In the case of the analogues Yb-compound (**2-Yb**) toluene was removed *in vacuo* and 10 mL of THF were added. Crystals for X-Ray diffraction of [{(NON)Yb(thf)$_2$}$_2$(μ-η^4:η^4-P_4)] (**2-Yb**) were obtained by recrystallisation from hot THF solution. In both cases crystals were separated from the mother liquor and dried *in vacuo*. Elemental analysis indicates removal of one molecule of THF from **2-Sm** and **2-Yb** upon drying *in vacuo*. Thus, above depiction of compounds reflects their molecular structure in the solid state as determined by single crystal X-ray diffraction, since the exact structure of the isolated solvate-free compounds was not investigated.

[{(NON)Sm(thf)}$_2$(μ-η^4:η^4-P_4)] (2-Sm): Yield: 127 mg (0.066 mmol), 60 %. **Anal. calcd**. (%) for [$C_{102}H_{140}N_4O_4P_4Sm_2$] (1910.88 g/mol): C: 64.11, H: 7.39, N: 2.93; **found** (%): C: 64.30, H: 7.09, N: 2.81. **^{31}P{^{1}H} NMR** (121.49 MHz, C_6D_6): δ [ppm]: 479.5 (s, P_4). **IR (ATR):** $\tilde{\nu}$ [cm^{-1}] = 3057 (w), 2960 (vs), 2928 (m), 2864 (m), 1772 (w), 1734 (w), 1717 (w), 1698 (w), 1685 (w), 1653 (w), 1617 (m), 1578 (w), 1559 (w), 1541 (w), 1519 (w), 1460 (w), 1431 (m), 1410 (m), 1392 (w), 1360 (w), 1329 (w), 1295 (m), 1271 (w), 1252 (w), 1220 (m), 1180 (m), 1141 (w), 1119 (w), 1094 (w), 1040 (w), 1015 (m), 932 (w), 882 (w), 861 (w), 838 (w), 793 (w), 769 (w), 757 (w), 739 (w), 703 (w), 659 (w), 575 (w), 547 (w), 521 (w), 456 (w), 435 (w), 361 (w).

[{(NON)Yb(thf)}$_2$(μ-η^4:η^4-P_4)] (2-Yb): Yield: 132 mg (0.067 mmol), 62 %. **Anal. calcd**. (%) for [$C_{102}H_{140}N_4O_4P_4Yb_2$] (1956.27 g/mol): C: 62.63, H: 7.21, N: 2.86; **found** (%): C: 62.87, H: 7.23, N: 2.29. **^{31}P{^{1}H} NMR** (121.49 MHz MHz, C_6D_6): δ [ppm]: 382.4 (s, P_4). **IR (ATR):** $\tilde{\nu}$ [cm^{-1}] = 3057 (w), 2961 (vs), 2902 (m), 2865 (m), 1621 (m), 1578 (w), 1517 (w), 1459 (m), 1434 (m), 1411 (w), 1392 (w), 1360 (w), 1332 (w), 1296 (m), 1271 (w), 1219 (w), 1181 (m), 1140 (w), 1119 (w), 1096 (w), 1069 (w), 1014 (m), 933 (w), 885 (w), 859 (w), 836 (w), 816 (w), 796 (w), 772 (w), 758 (w), 739 (w), 659 (w), 633 (w), 576 (w), 523 (w), 436 (w), 389 (m).

Note: Due to the paramagnetic behaviour of compounds **2-Sm** and **2-Yb** no meaningful ^{1}H- and ^{13}C-NMR were obtained. Nevertheless, ^{31}P{^{1}H} NMR-spectra show one singlet for the four equivalent phosphorus atoms in both cases (see Fig. S3 and S4)

Synthesis of [(NON)Ln(BH_4)($thf)_2$] (3)

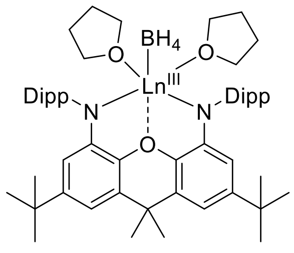

Ln = Y (**3-Y**), Sm (**3-Sm**), Dy (**3-Dy**)

In a general procedure to synthesize [(NON)Ln(BH_4)($thf)_2$] (Ln = Y, Sm, Dy) 20 mL THF was added to a mixture of 500 mg [K_2(NON)] (0.667 mmol, 1.00 eq.) and the corresponding [Ln($BH_4)_3$($thf)_3$] (0.667 mmol, 1.00 mmol, Ln = Y: 233 mg, Sm: 274 mg, Dy: 283 mg). The reaction mixture was stirred for 16 h at room temperature and all volatiles were removed *in vacuo*. The oily residue was extracted with 30 mL of hot *n*-heptane and filtered while still hot. The residue was washed with another 20 mL hot *n*-heptane. Upon cooling to room temperature colourless (**3-Y**) and light green (**3-Dy**) crystals suitable for single crystal X-ray formed. The analogues Sm-compound **3-Sm** was crystallized as red crystals in the same way. To improve the crystalline yield, the *n*-heptane solutions were stored at -30 °C before crystals were separated from the mother liquor and dried *in vacuo*. Elemental analysis indicates removal of one molecule of THF in compounds **3-Ln** upon drying *in vacuo*. The ^{1}H NMR spectrum of compound **3-Y** confirms this assumption.

[(NON)Y(BH_4)(thf)] (3-Y): Yield: 390 mg (0.461 mmol), 69 %. **Anal. calcd**. (%) for [$C_{51}H_{74}BN_2O_2Y$] (846.88 g/mol): C: 72.33, H: 8.81, N: 3.31; **found** (%): C: 72.52, H: 8.34, N: 3.01. **^{1}H{^{11}B} NMR** (400.30 MHz, C_6D_6): δ [ppm]: 7.29-7.19 (m, 6 H, Dipp-C*H*$_{ar}$), 6.76 (d, $^4J_{HH}$ = 2.1 Hz, 2 H, Xanth-*p*-C*H*), 6.16 (d, $^4J_{HH}$ = 2.1 Hz, 2 H, Xanth-*o*-C*H*), 3.54 (sept., $^3J_{HH}$ = 6.6 Hz, 4 H, Dipp-C*H*($CH_3)_2$), 3.43-3.39 (m, 4 H, thf-OC*H*$_2$), 1.58 (s, 6 H, Xanth-C(C*H*$_3)_2$), 1.31 (d, $^3J_{HH}$ = 6.9 Hz, 12 H, Dipp-CH(C*H*$_3)_2$), 1.28 (s, 18 H, Xanth-C(C*H*$_3)_3$), 1.21 (d, $^3J_{HH}$ = 6.8 Hz, 12 H, Dipp-CH(C*H*$_3)_2$), 1.13 (s, 4 H, B*H*$_4$), 1.00-0.96 (m, 4 H, thf-C*H*$_2$); **^{11}B NMR** (128.43 MHz, C_6D_6) δ [ppm]: -24.2 (br, *B*H_4); **^{13}C{^{1}H} NMR** (100.67 MHz, C_6D_6): δ [ppm]: 148.1, 147.4, 146.0, 143.8, 140.4, 130.1, 125.8, 124.4 (Ar-*C*), 109.2 (Xanth-*o*-*C*H), 107.3 (Xanth-*p*-*C*H), 72.3 (thf-O*C*H_2), 35.3 (Xanth-*C*($CH_3)_2$), 35.1 (*C*($CH_3)_3$), 31.9 (C(*C*$H_3)_3$), 30.9 (Xanth-C(*C*$H_3)_2$), 28.6 (Dipp-*C*H($CH_3)_2$), 26.0 (Dipp-CH(*C*$H_3)_2$), 25.1 (thf-*C*H_2), 25.1 (Dipp-CH(*C*$H_3)_2$). **IR (ATR):** $\tilde{\nu}$ [cm^{-1}] = 3067 (w), 2961 (vs), 2904 (m), 2867 (m), 2497 (w), 2442 (w), 2382 (w), 2349 (w), 2219 (w), 2162 (w), 1623 (m), 1582 (w), 1517 (w), 1500 (w), 1461 (m), 1439 (m), 1411 (w), 1392 (w), 1361 (w); 1333 (w), 1298 (w), 1248 (w). 1217 (m), 1140 (w), 1114 (w), 1099 (w), 1056 (w), 1016 (w), 935 (w), 890 (w), 875 (w), 850 (w), 801 (w), 772 (w), 759 (w), 706 (w), 657 (w), 523 (w), 422 (w).

Note: The signal at 1.13 ppm (B*H*$_4$) could only be detected in the ^{1}H{^{11}B} NMR. No resonance for the borohydride is observed in the ^{1}H NMR.

[(NON)Sm(BH_4)(thf)] (3-Sm): Yield: 485 mg (0.534 mmol), 80 %. **Anal. calcd**. (%) for [$C_{51}H_{74}BN_2O_2Sm$] (908.34 g/mol): C: 67.44, H: 8.21, N: 3.08; **found** (%): C: 67.54, H: 8.13, N: 2.75. **^{11}B NMR** (128.43 MHz, C_6D_6) δ [ppm]: -38.5 (br, *B*H_4). **IR (ATR):** $\tilde{\nu}$ [cm^{-1}] = 3064 (w), 3026 (w), 2961 (vs), 2928 (m), 2867 (m), 2427 (w), 2376 (w), 2323(w), 2277(w), 2217 (w), 2164 (w), 1624 (m), 1585 (w), 1517 (m), 1498 (m), 1461 (m), 1442 (m), 1411 (m), 1391 (m), 1361 (m), 1340 (m), 1297 (m), 1255 (w), 1218 (s), 1178 (w), 1141 (w), 1112(w), 1057 (w), 1016 (w), 935 (w), 875 (w), 849 (w), 800 (w), 772 (w), 759 (w), 730 (w), 695 (w), 656 (w), 624 (w), 538 (w), 511 (w), 464 (w), 418 (w).

[(NON)Dy(BH_4)(thf)] (3-Dy): Yield: 393 mg (0.427 mmol), 64%. **Anal. calcd**. (%) for [$C_{51}H_{74}BN_2O_2Dy$] (920.48 g/mol): C: 66.55, H: 8.10, N: 3.04; **found** (%): C: 65.95, H: 7.90, N: 2.88. **IR (ATR):** $\tilde{\nu}$ [cm^{-1}] = 3067 (w), 2960 (vs), 2905 (m), 2867 (m), 2491 (w), 2445 (w), 2382 (w), 2349 (w), 2217 (w), 2145 (w), 1623 (m), 1582 (w), 1517 (m), 1500 (w), 1461 (m), 1439 (m), 1411 (w), 1392 (m), 1361 (w), 1333 (w),

1298 (w), 1248 (w), 1217 (m), 1141 (w), 1115 (w), 1098 (w), 1056 (w), 1016 (w), 935 (w), 891 (w), 875 (w), 851 (w), 802 (w), 771 (w), 758 (w), 707 (w), 658 (w), 523 (w), 461 (w), 409 (w).
Note: Due to the paramagnetic behaviour of compounds **3-Sm** and **3-Dy** no meaningful ^{1}H and ^{13}C NMR spectra could be obtained. Nevertheless, for compound **3-Sm** one resonance in the ^{11}B NMR was observed (See Fig.S8), while the ^{11}B NMR for compound **3-Dy** remained silent.

Synthesis of [K{(NON)Ln(thf)}$_2$(μ_3-η^3:η^3:η^2-P$_3$)] (4)

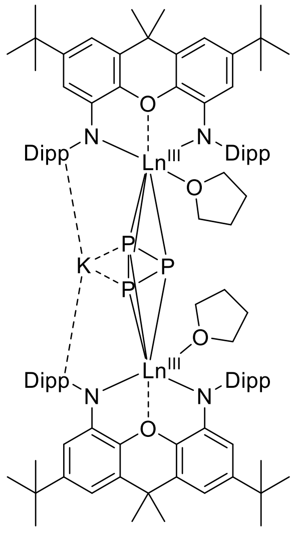

Ln = Y (**4-Y**), Sm (**4-Sm**), Dy (**4-Dy**)

Method A (Ln = Y, Sm, Dy): In an one pot reaction 15 mL of toluene was condensed to 0.220 mmol [(NON)Ln(BH$_4$)(thf)] (Ln = Y: 186 mg, Sm: 200 mg, Dy: 203 mg; 2.00 eq.), 13.6 mg P$_4$ (0.110 mmol, 1.00 eq.) and 17.2 mg potassium (0.440 mmol, 4.00 eq.) at -78 °C. The resulting suspension was stirred at 65 °C for three days. After filtering off the excess of potassium and insoluble residues, the clear solution was transferred to a double section ampule, degassed and flame sealed. Crystals suitable for single crystal X-ray diffraction of compounds **4** were grown of the toluene solution by slow evaporation. Storing of the toluene solution after filtration at -10 °C also leads to analytically pure crystals, which were used for further characterisation.
Method B (Ln = Sm): Toluene (10 mL) was added to 85.0 mg [{(NON)Sm(thf)}$_2$(μ-η^4:η^4-P$_4$)] (**2-Sm**) (0.044 mmol, 1.00 eq.) and 10.0 mg potassium (0.256 mmol, ex.). The subsequent suspension was stirred at 65 °C for 16 h. Thereby a color change from deep red to pink was observed. After filtration, all volatile residues were removed *in vacuo*. The remaining solid was extracted with Et$_2$O and filtered in a double section ampule. Crystals of compound **4-Sm** were formed by slow evaporation of Et$_2$O. The formation of compound **4-Sm** by reduction of compound **2-Sm** was also observed in a NMR scale reaction.
[K{(NON)Y(thf)}$_2$(μ_3-η^3:η^3:η^2-P$_3$)] (4-Y): Yield: 52.0 mg (0.029 mmol), 26%. **Anal. calcd**. (%) for [C$_{102}$H$_{140}$KN$_4$O$_4$P$_3$Y$_2$] (1796.10 g/mol): C: 68.21, H: 7.86, N: 3.12; **found** (%): C: 68.74, H: 7.69, N: 2.79. **^{1}H NMR** (400.30 MHz, thf-d$_8$): δ [ppm]: 6.96-6.84 (m, 12 H, Dipp-C*H*$_{ar}$), 6.17 (d, $^{4}J_{HH}$ = 2.3 Hz, 4 H, Xanth-*p*-C*H*), 5.43 (d, $^{4}J_{HH}$ = 2.2 Hz, 4 H, Xanth-*o*-C*H*), 3.63-3.60 (m, 8 H, thf-OC*H*$_2$), 3.27 (sept., $^{3}J_{HH}$ = 6.8 Hz, 8 H, Dipp-C*H*(CH$_3$)$_2$), 1.79-1.76 (m, 8 H, thf-C*H*$_2$), 1.49 (s, 12 H, Xanth-C(C*H*$_3$)$_2$), 1.29 (d, $^{3}J_{HH}$ = 7.0 Hz, 24 H, Dipp-CH(C*H*$_3$)$_2$), 1.01 (s, 36 H, Xanth-C(C*H*$_3$)$_3$), 0.87 (d, $^{3}J_{HH}$ = 6.8 Hz, 24 H, Dipp-CH(C*H*$_3$)$_2$). **^{13}C{^{1}H} NMR** (100.67 MHz, thf-d$_8$): δ [ppm]: 152.3, 149.7, 145.4, 144.8, 139.8, 128.6 124.0, 122.9 (Ar-*C*), 109.5 (Xanth-*o*-*C*H), 102.7 (Xanth-*p*-*C*H), 68.0 (thf-O*C*H$_2$), 35.1 (Xanth-*C*(CH$_3$)$_2$), 34.9 (*C*(CH$_3$)$_3$), 32.2 (C(*C*H$_3$)$_3$), 31.9 (Xanth-C(*C*H$_3$)$_2$), 28.7 (Dipp-*C*H(CH$_3$)$_2$), 26.9 (Dipp-CH(*C*H$_3$)$_2$),, 25.9 (thf-*C*H$_2$). **^{31}P{^{1}H} NMR** (121.49 MHz MHz, thf-d$_8$): δ [ppm]: -240.3 (t, $^{1}J_{YP}$ = 20.2 Hz, *P*$_3$). **IR (ATR):** $\tilde{\nu}$ [cm^{-1}] = 3062 (w), 2958 (vs), 2904 (m), 2865 (m), 1616 (m), 1577 (m), 1517 (w), 1479 (m), 1459 (m), 1429 (m), 1410 (m), 1392 (w), 1359 (w), 1304 (m), 1243 (m), 1218 (m), 1195 (m), 1139 (w), 1118 (w), 1054 (w), 1014 (m), 939 (w), 860 (w), 800 (w), 776 (w), 761 (w), 728 (w), 694 (w), 660 (w), 634 (w), 605 (w), 544 (w), 522 (w), 464 (w), 429 (w).

[K{(NON)Sm(thf)}$_2$(μ_3-η^3:η^3:η^2-P$_3$)] (4-Sm): Yield: 73.0 mg (0.038 mmol), 39 %. **Anal. calcd**. (%) for [$C_{102}H_{140}KN_4O_4P_3Sm_2$] (1919.01 g/mol): C: 63.84, H: 7.35, N: 2.92; **found** (%): C: 64.08, H: 7.12, N: 2.57. **^{31}P{^{1}H} NMR** (121.49 MHz MHz, thf-d$_8$): δ [ppm]: -336.7 (s, P_3). **IR (ATR):** $\tilde{\nu}$ [cm^{-1}] = 3064 (w), 2961 (vs), 2905 (m), 2867 (m), 1779 (w), 1707 (w), 1623 (m), 1517 (m), 1498 (w), 1462 (m), 1442 (m), 1410 (w), 1392 (m), 1361 (w) 1334 (w), 1298 (w), 1255 (w), 1218 (m), 1180 (w), 1141 (w), 1117 (w), 1056 (w), 1014 (w), 934 (w), 849 (w), 801 (w), 772 (w), 759 (w), 728 (w), 694 (w), 657 (w), 537 (w), 464 (w).

[K{(NON)Dy(thf)}$_2$(μ_3-η^3:η^3:η^2-P$_3$)] (4-Dy): Yield: 94.0 mg, (0.048 mmol), 44 %. **Anal. calcd**. (%) for [$C_{102}H_{140}KN_4O_4P_3Dy_2$] (1943.29 g/mol): C: 63.04, H: 7.26, N: 2.88; **found** (%): C: 63.05, H: 7.23, N: 2.60. **^{31}P{^{1}H} NMR** (121.49 MHz MHz, thf-d$_8$): δ [ppm]: -245.5 (s, P_3). **IR (ATR):** $\tilde{\nu}$ [cm^{-1}] = 3064 (w), 2960 (vs), 2905 (m), 2866 (m), 1779 (w), 1708 (w), 1622 (m), 1579 (w), 1517 (m), 1496 (m), 1462 (m), 1442 (m), 1411 (w), 1392 (m), 1360 (w), 1333 (w), 1300 (w), 1244 (w), 1218 (m), 1140 (w), 1118 (w), 1056 (w), 1015 (w), 986 (w), 935 (w), 859 (w), 802 (w), 775 (w), 760 (w), 729 (w), 695 (w), 658 (w), 538 (w), 464 (w), 371 (w).

Note: Due to the paramagnetic behaviour of compounds **4-Sm** and **4-Dy** no meaningful ^{1}H and ^{13}C NMR spectra could be obtained. Nevertheless, one resonance in the ^{31}P{^{1}H} NMR was observed for both compounds (see Fig. S12 and S13).

3. NMR Spectra

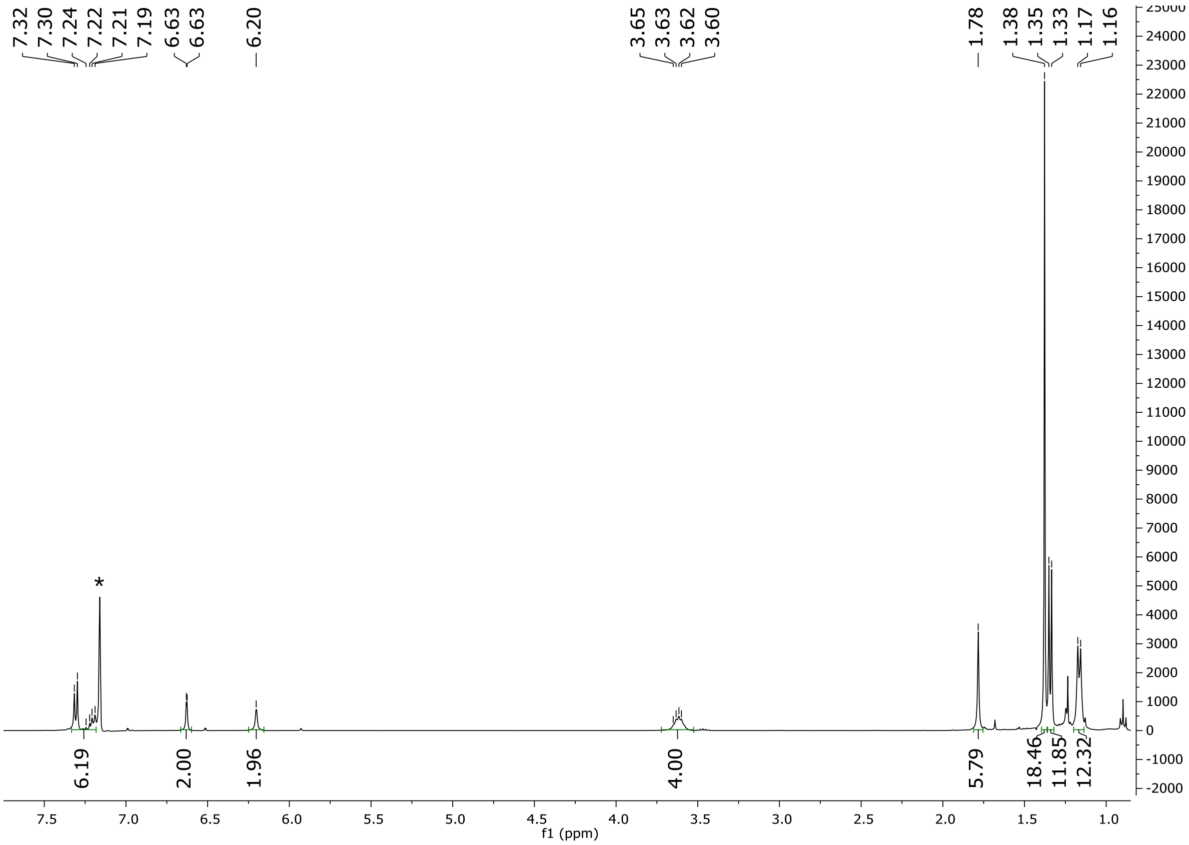

Fig. S 1: ^{1}H NMR spectrum of [(NON)Yb] (**1-Yb**) in C_6D_6 at 25 °C. *, residual protio solvent signal.

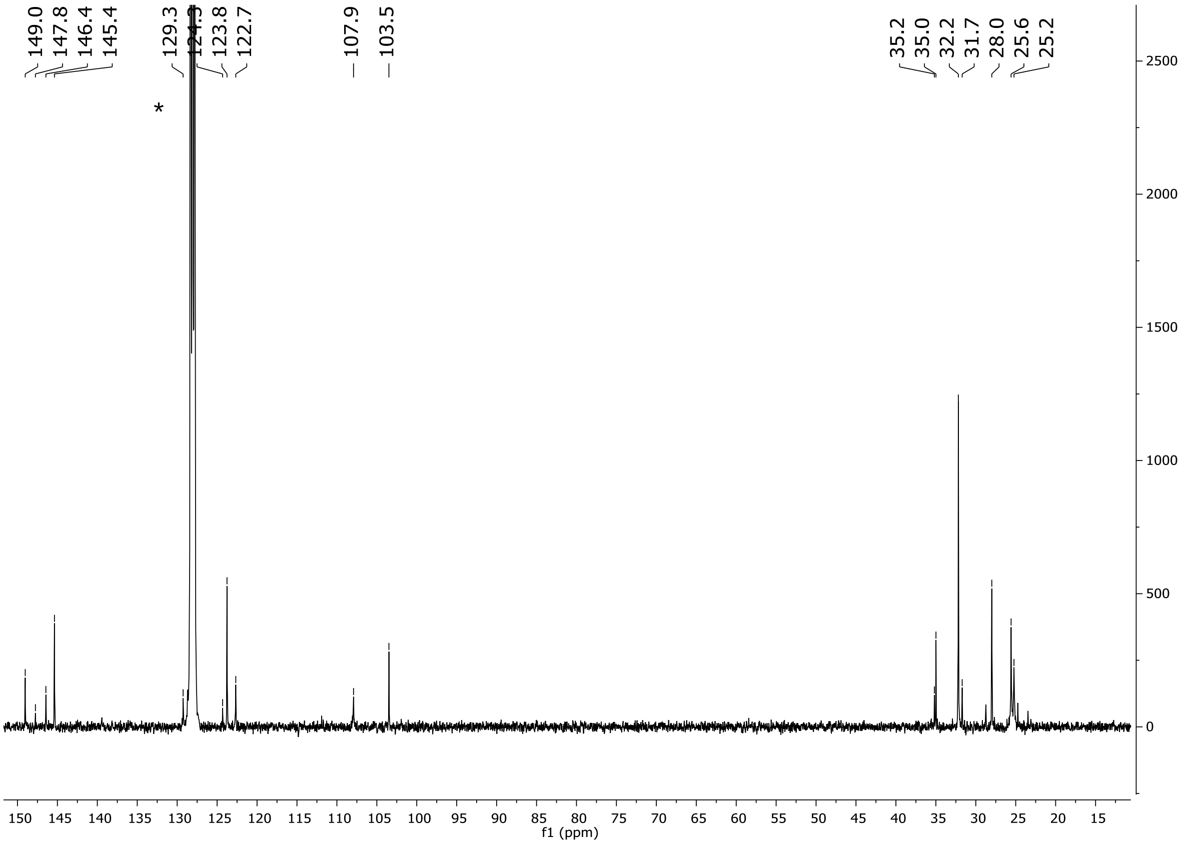

Fig. S 2: $^{13}C\{^{1}H\}$ NMR spectrum of [(NON)Yb] (**1-Yb**) in C_6D_6 at 25 °C. *, C_6D_6 signal.

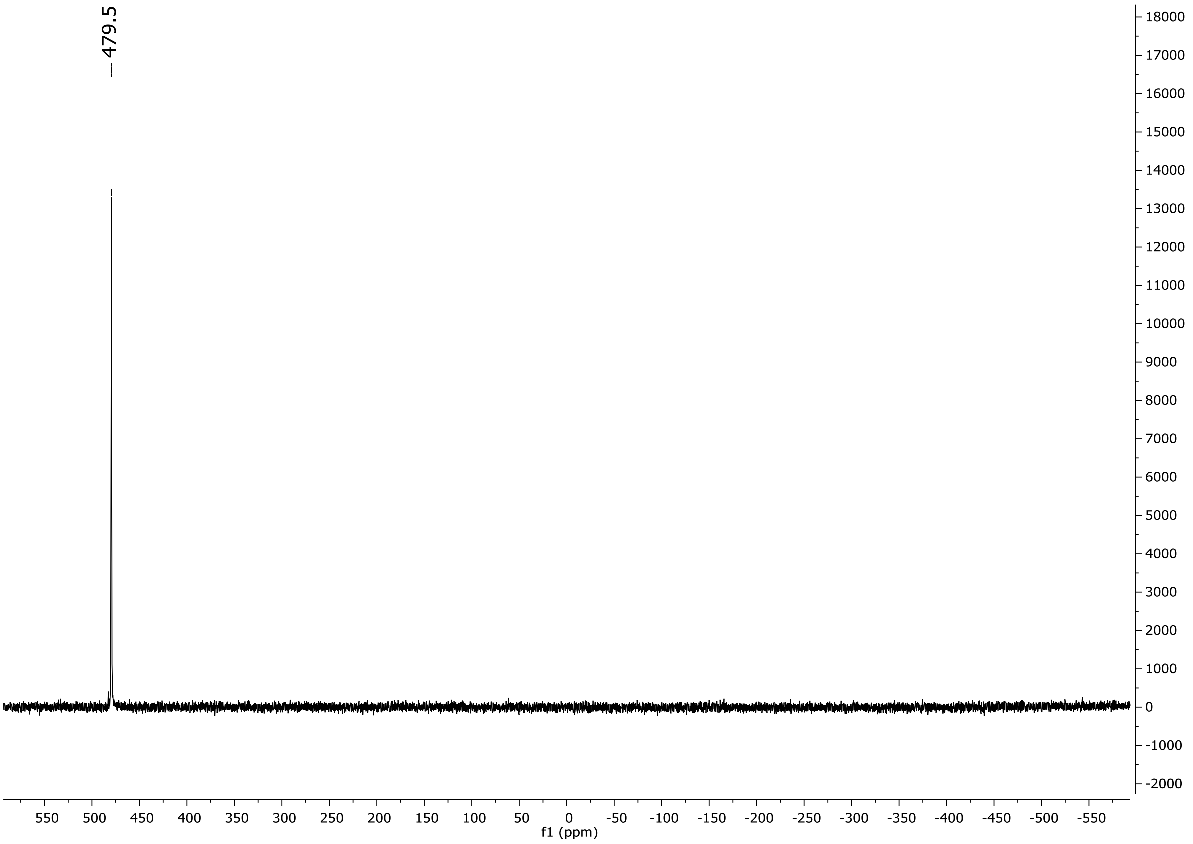

Fig. S 3: $^{31}P\{^{1}H\}$ NMR spectrum of [{(NON)Sm(thf)}$_2$(μ-η^4:η^4-P_4)] (**2-Sm**) in C_6D_6 at 25 °C.

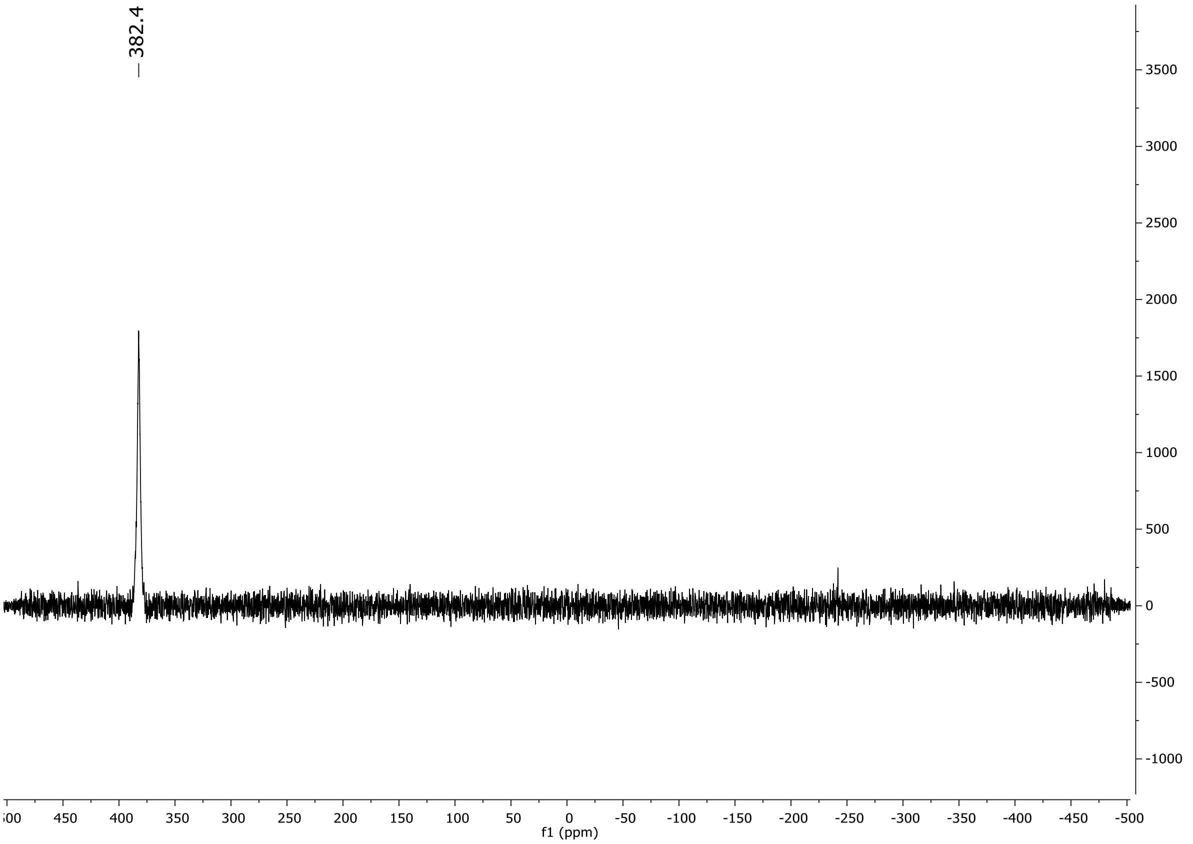

Fig. S 4: $^{31}P\{^{1}H\}$ NMR spectrum of [{(NON)Yb(thf)}$_2$(μ-η^4:η^4-P_4)] (**2-Yb**) in C_6D_6 at 25 °C.

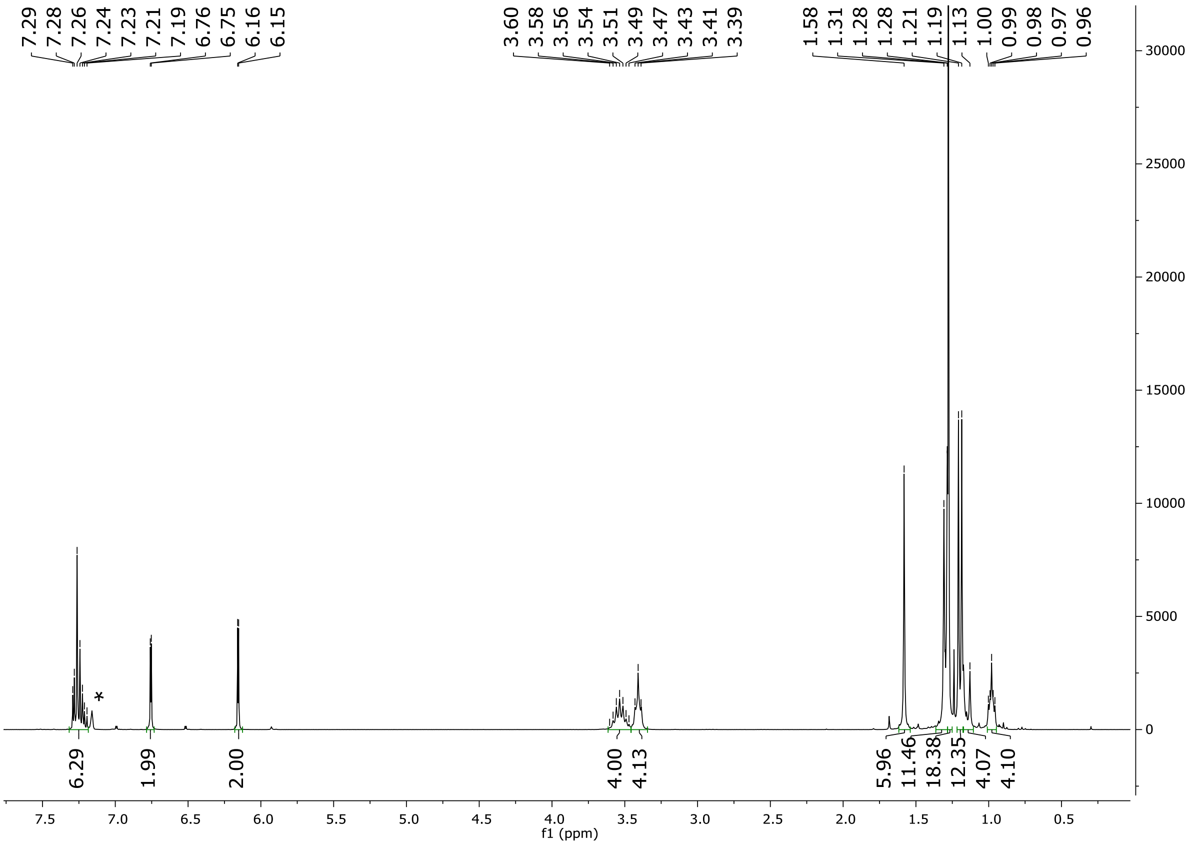

Fig. S 5: $^{1}H\{^{11}B\}$ NMR spectrum of [(NON)Y(BH_4)(thf)] (**3-Y**) in C_6D_6 at 25 °C. *, residual protio solvent signal.

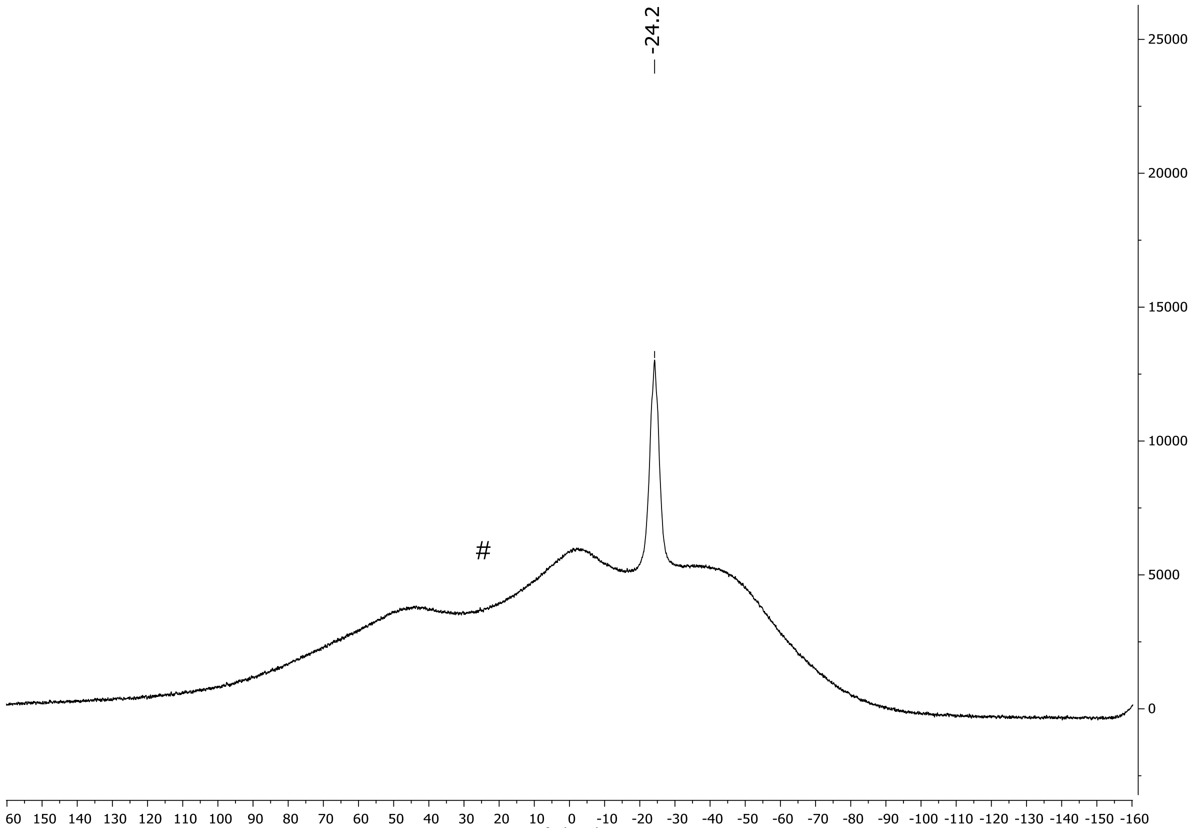

Fig. S 6: ^{11}B NMR spectrum of [(NON)Y(BH_4)(thf)] (**3-Y**) in C_6D_6 at 25 °C. #, background signal from NMR tube (made of borosilicate glass).

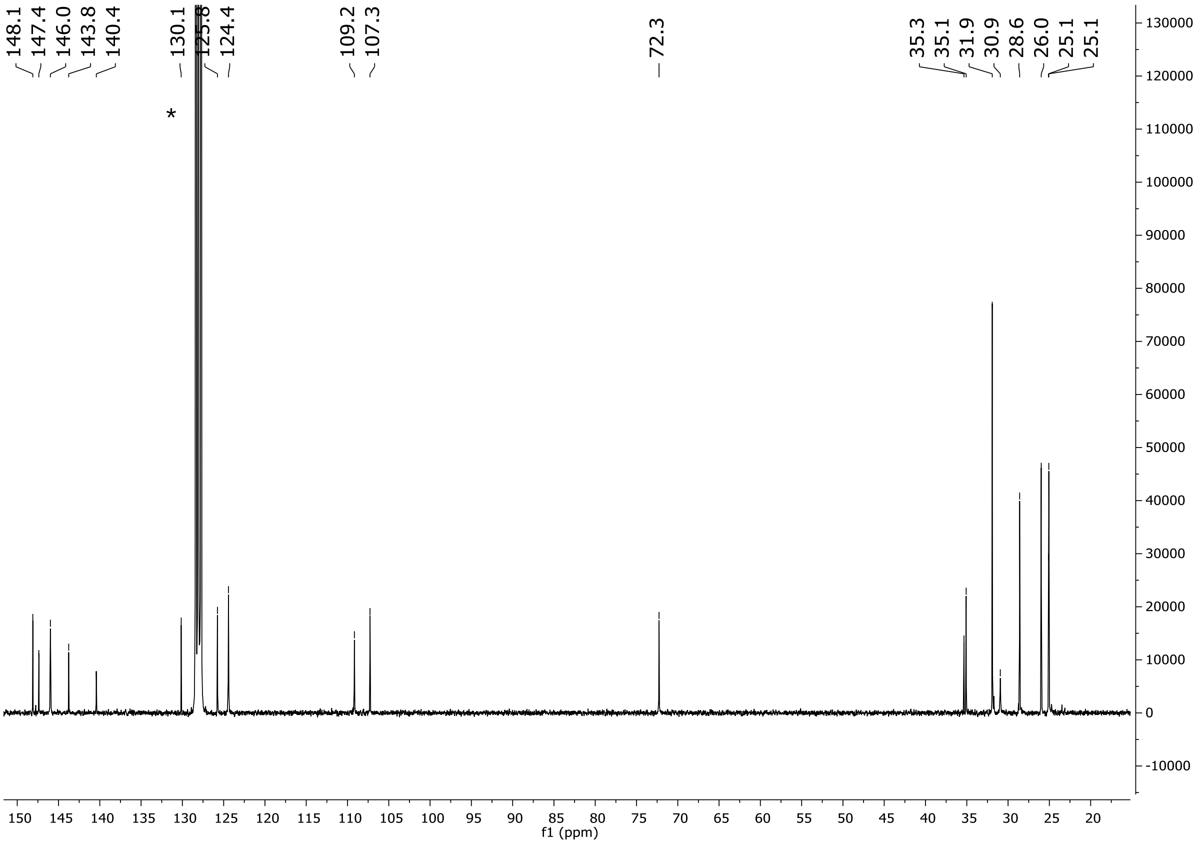

Fig. S 7: $^{13}C\{^{1}H\}$ NMR spectrum of [(NON)Y(BH$_4$)(thf)] (**3-Y**) in C_6D_6 at 25 °C. *, C_6D_6 signal.

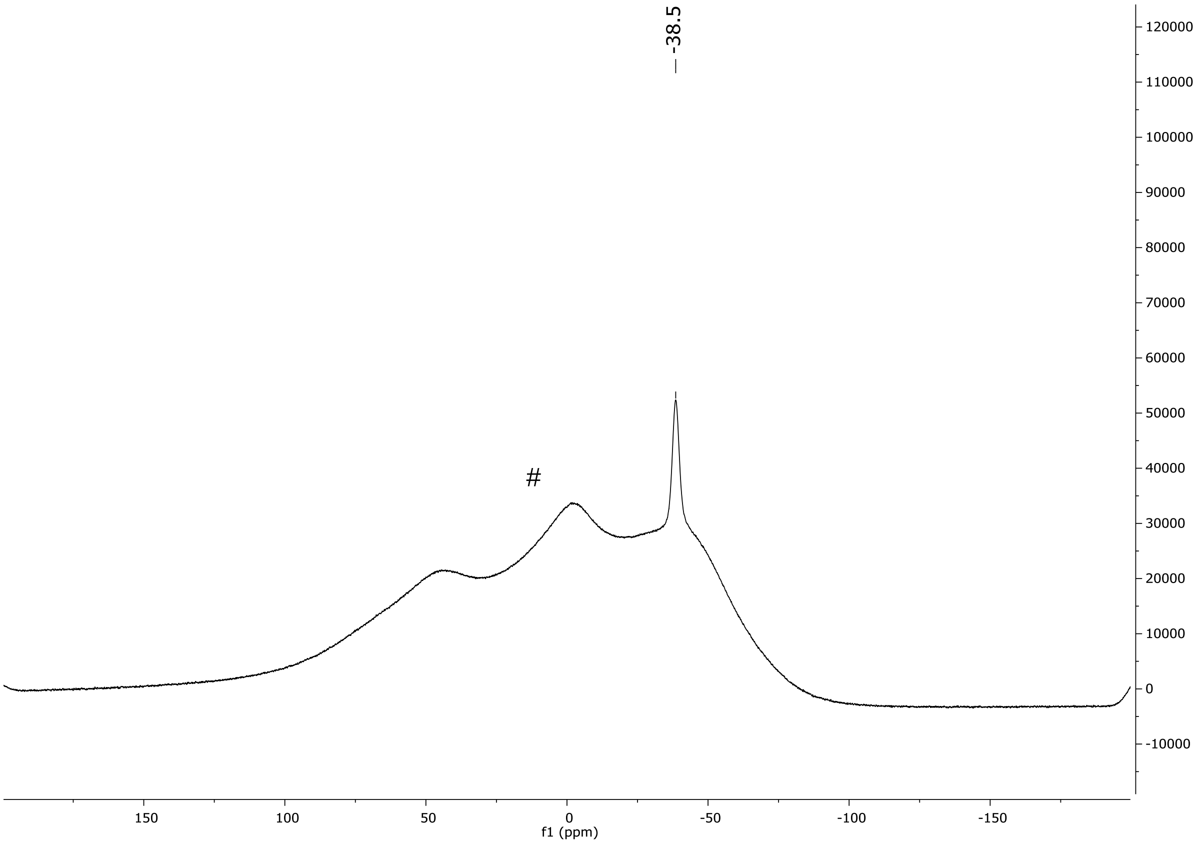

Fig. S 8: ^{11}B NMR spectrum of [(NON)Sm(BH$_4$)(thf)] (**3-Sm**) in C_6D_6 at 25 °C. #, background signal from NMR tube (made of borosilicate glass).

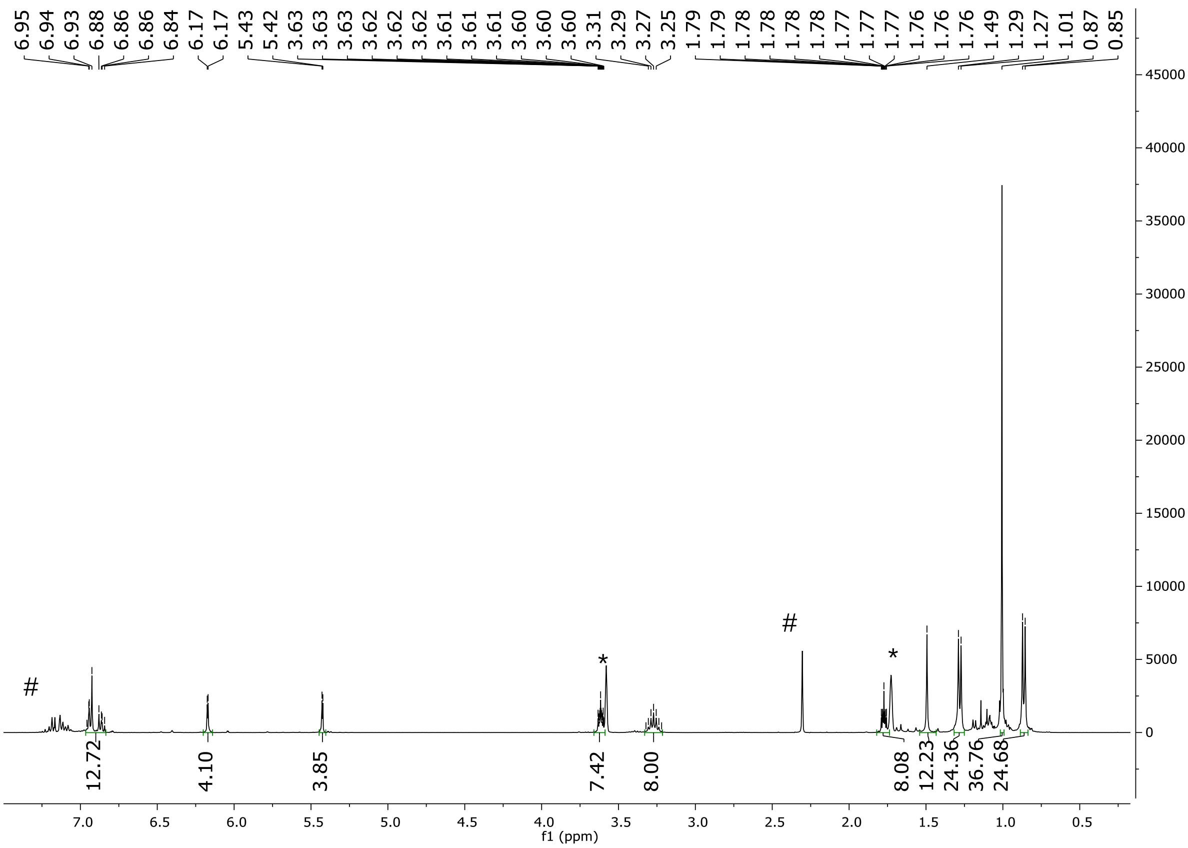

Fig. S 9: ^{1}H NMR spectrum [K{(NON)Y(thf)}$_2$(μ_3-η^3:η^3:η^2-P_3)] (**4-Y**) in thf-d$_8$ at 25 °C. *, residual protio solvent signals. #, toluene signals (crystallized from toluene solution).

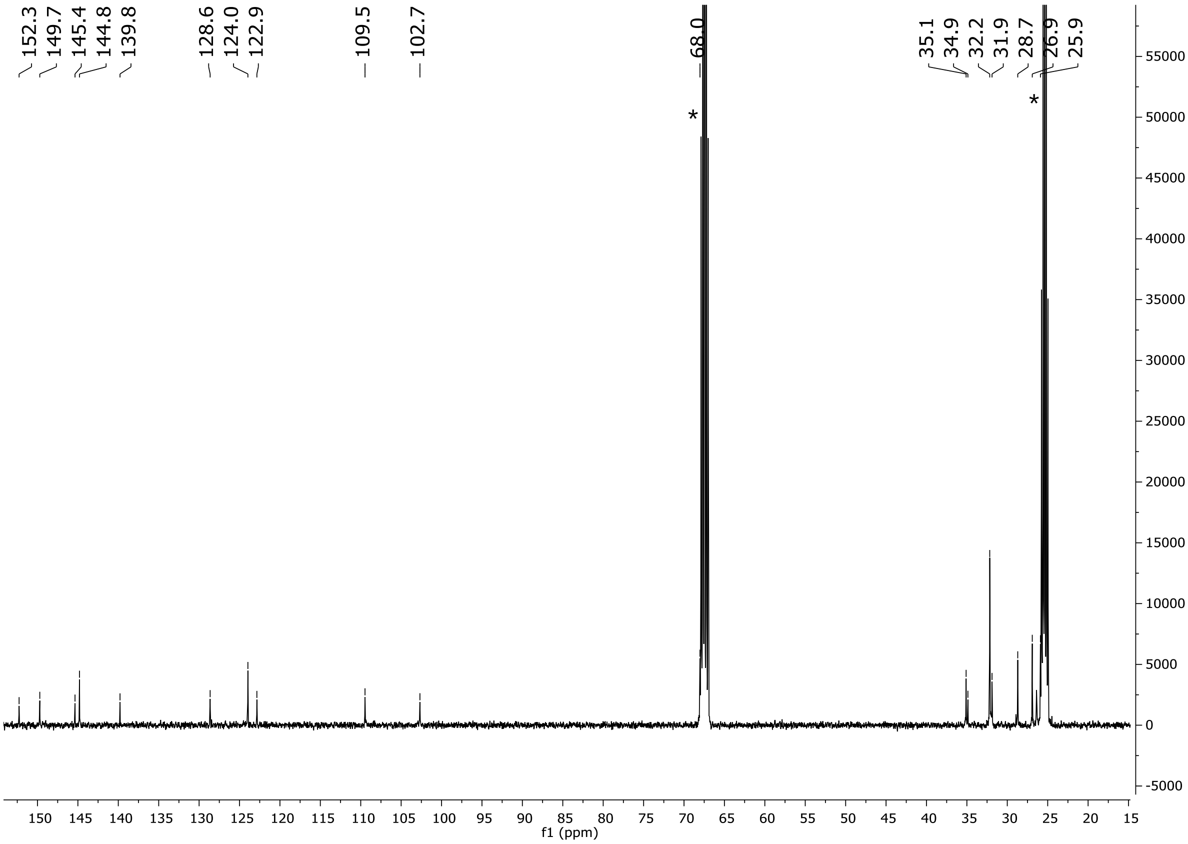

Fig. S 10: ^{13}C{^{1}H} NMR spectrum of [K{(NON)Y(thf)}$_2$(μ_3-η^3:η^3:η^2-P_3)] (**4-Y**) in thf-d$_8$ at 25 °C. *, thf-d$_8$ signals.

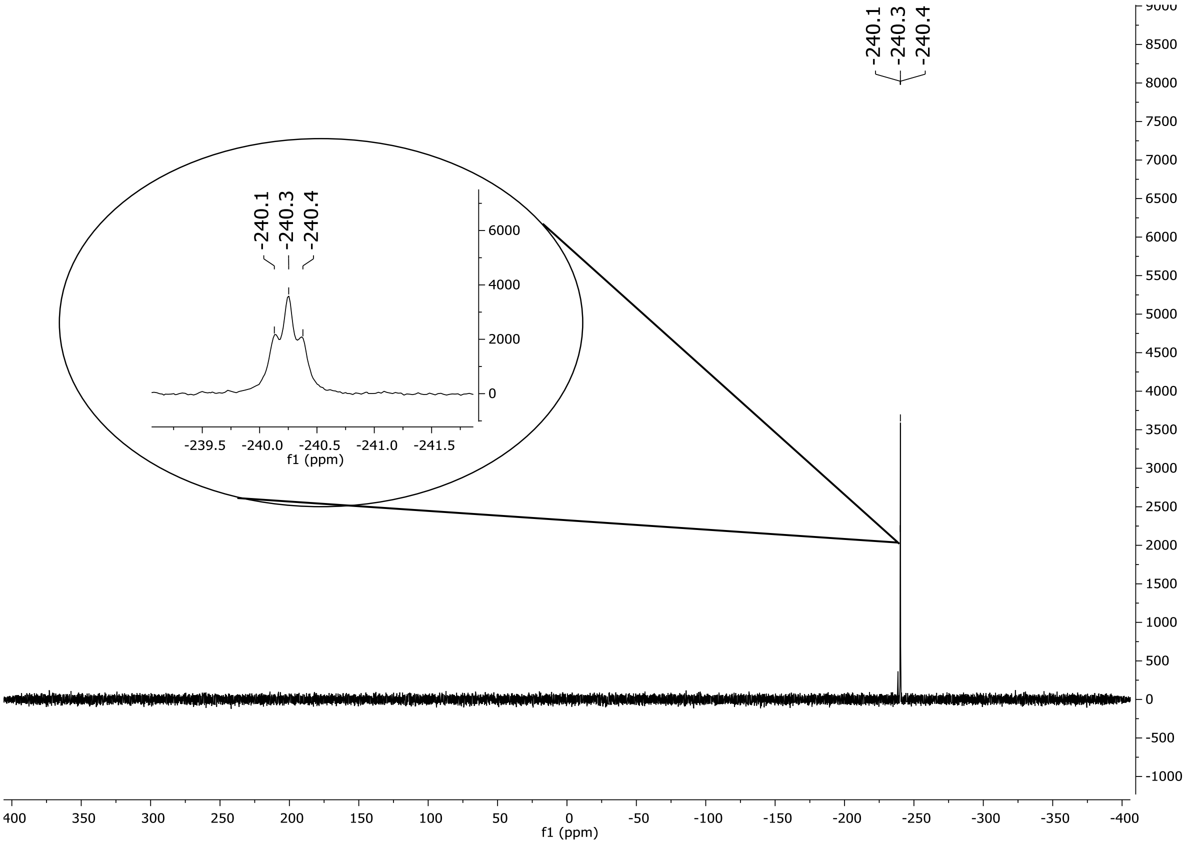

Fig. S 11: $^{31}P\{^1H\}$ NMR spectrum of $[K\{(NON)Y(thf)\}_2(\mu_3\text{-}\eta^3\text{:}\eta^3\text{:}\eta^2\text{-}P_3)]$ (**4-Y**) in thf-d_8 at 25 °C.

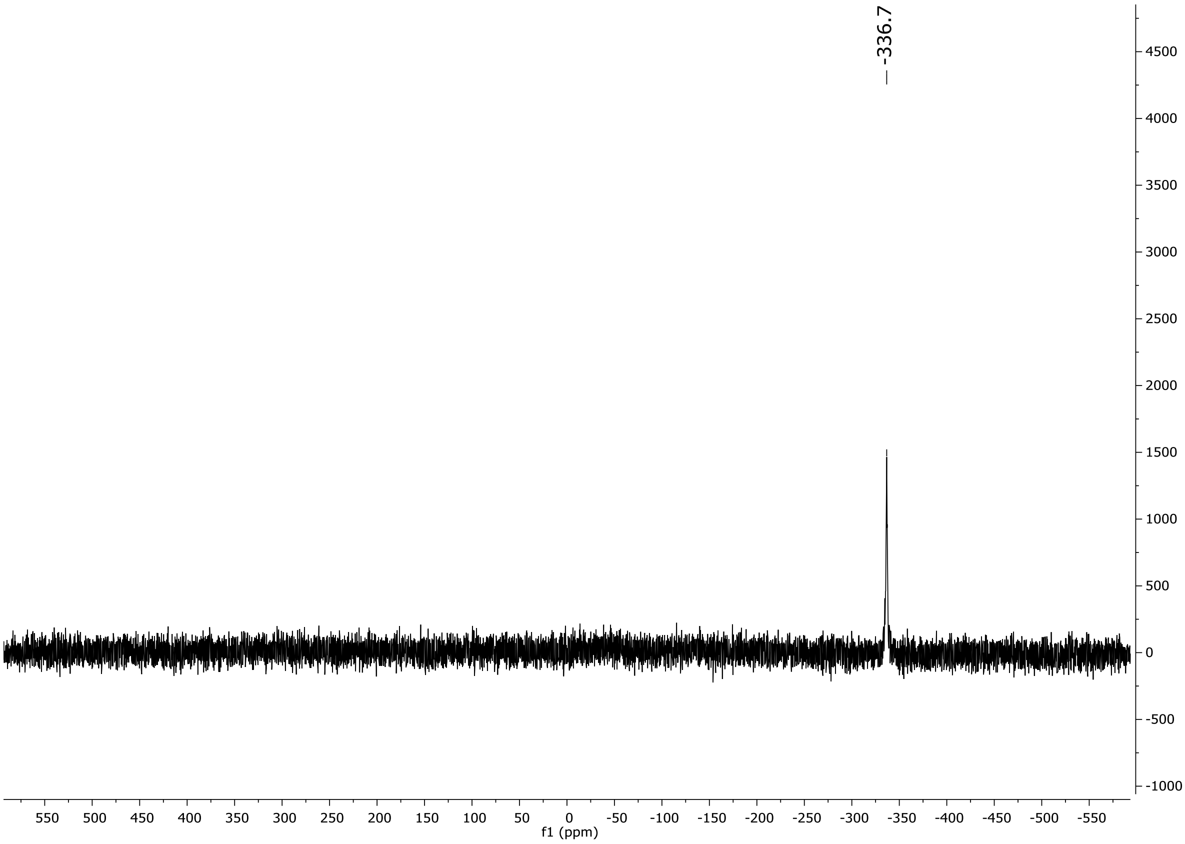

Fig. S 12: $^{31}P\{^1H\}$ NMR spectrum of $[K\{(NON)Sm(thf)\}_2(\mu_3\text{-}\eta^3\text{:}\eta^3\text{:}\eta^2\text{-}P_3)]$ (**4-Sm**) in thf-d_8 at 25 °C.

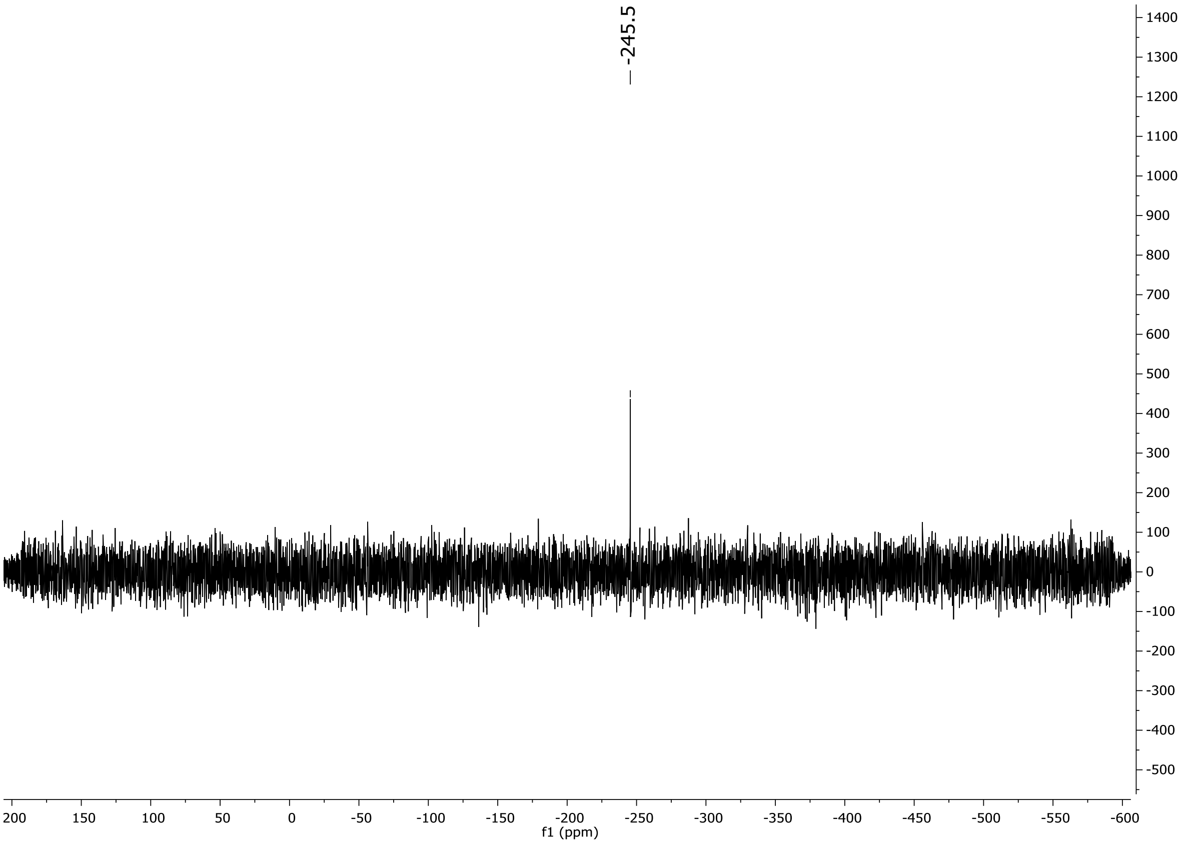

Fig. S 13: $^{31}P\{^{1}H\}$ NMR spectrum of [K{(NON)Dy(thf)}$_2$(μ_3-η^3:η^3:η^2-P_3)] (**4-Dy**) in thf-d_8 at 25 °C.

4. IR Spectra

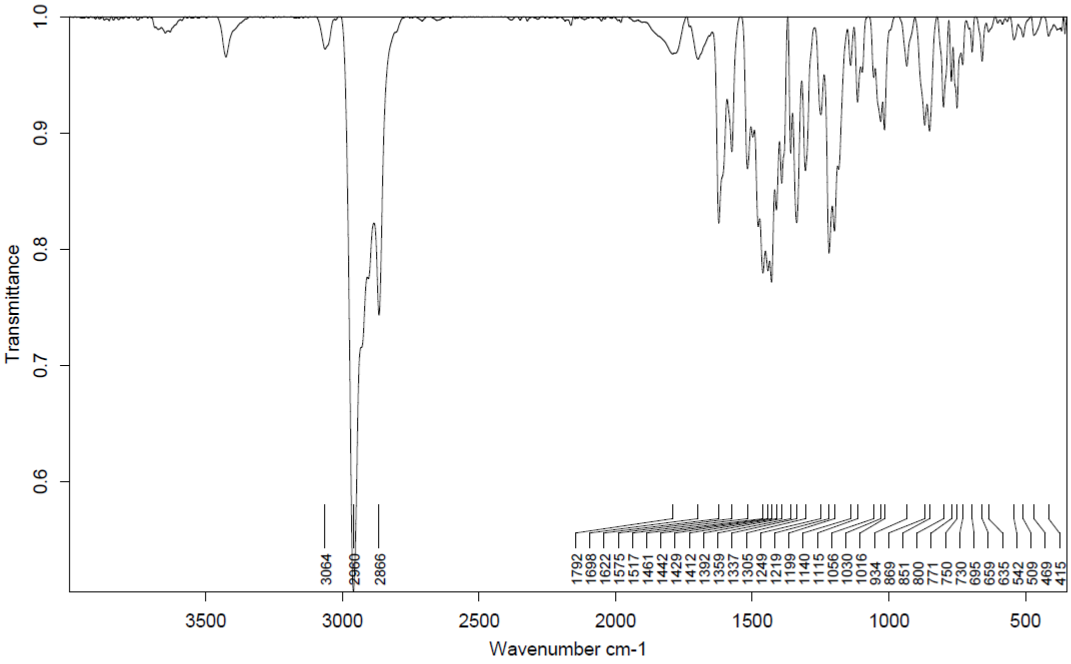

Fig. S 14: IR spectrum (ATR) of [(NON)Sm(thf)] (**1-Sm**).

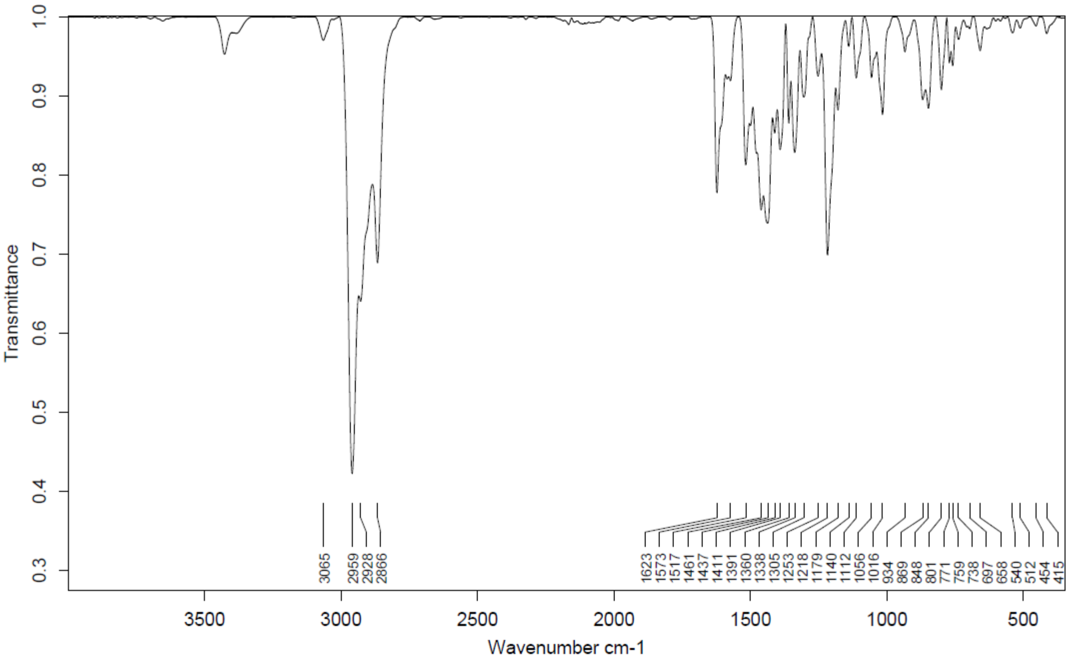

Fig. S 15: IR spectrum (ATR) of [(NON)Yb] (**1-Yb**).

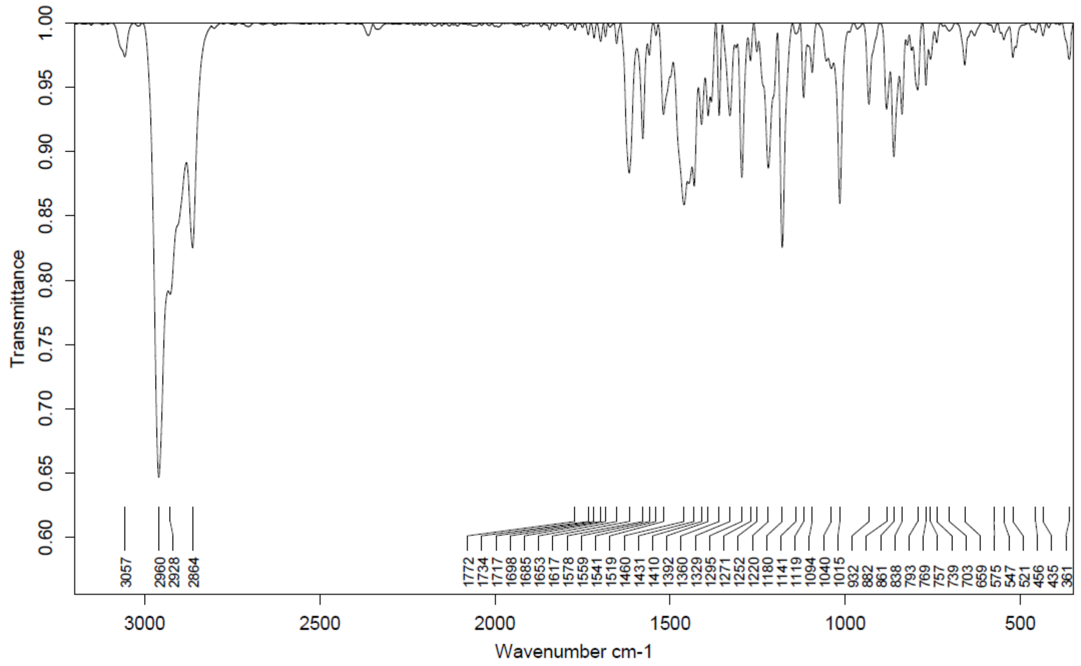

Fig. S 16: IR spectrum (ATR) of [{(NON)Sm(thf)}$_2$(μ-η^4:η^4-P_4)] (**2-Sm**).

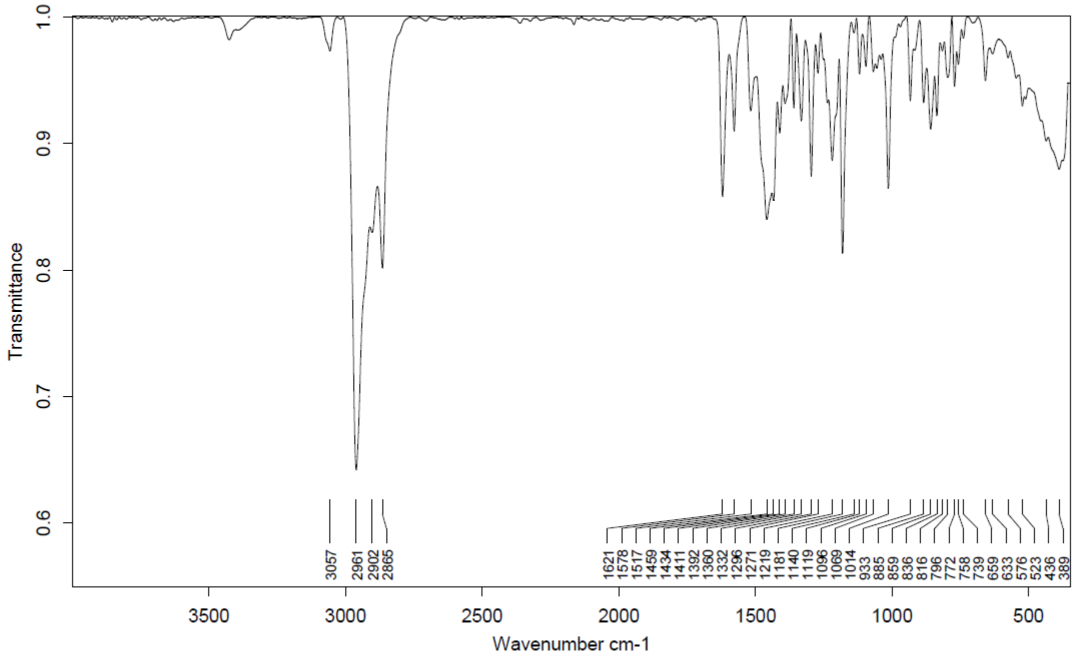

Fig. S 17: IR spectrum (ATR) of [{(NON)Yb(thf)}$_2$(μ-η^4:η^4-P_4)] (**2-Yb**).

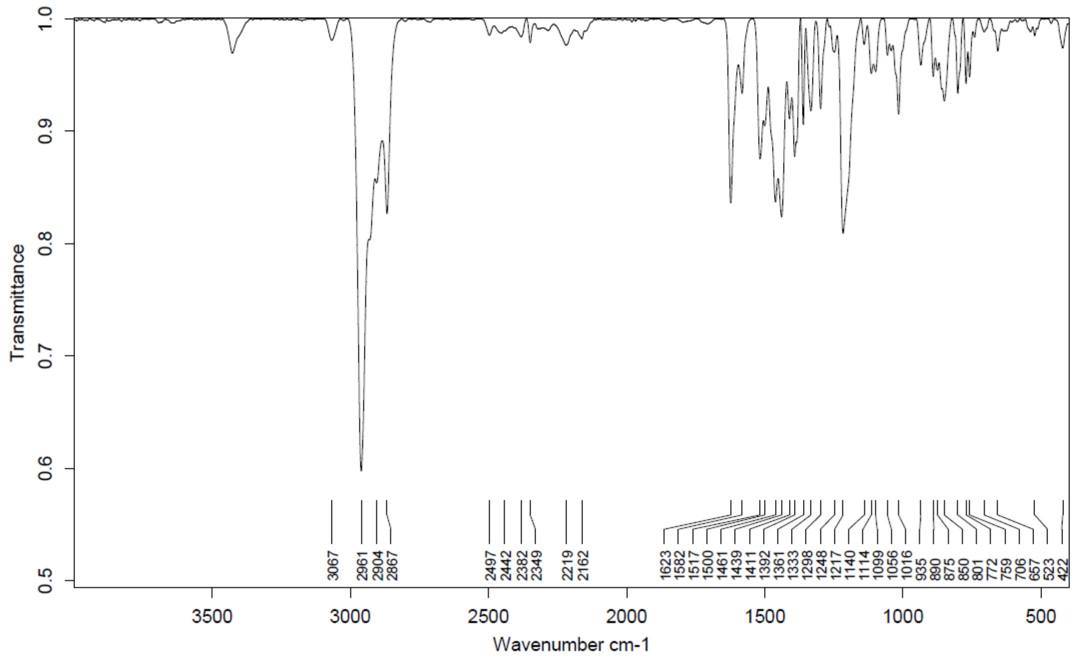

Fig. S 18: IR spectrum (ATR) of [(NON)Y(BH_4)(thf)] (**3-Y**).

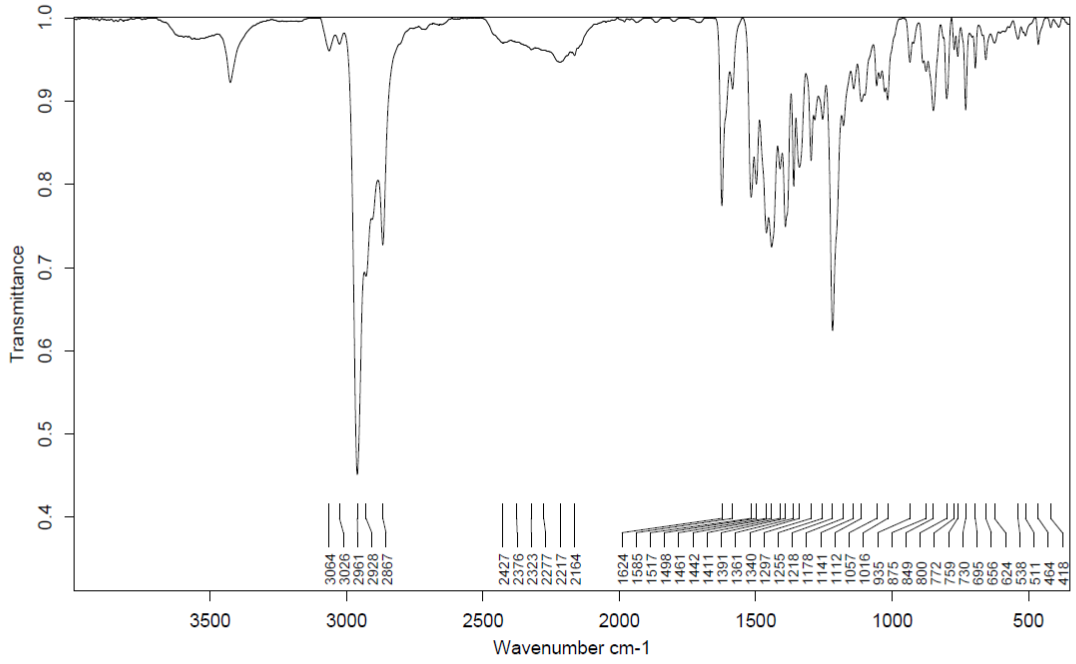

Fig. S 19: IR spectrum (ATR) of [(NON)Sm(BH_4)(thf)] (**3-Sm**).

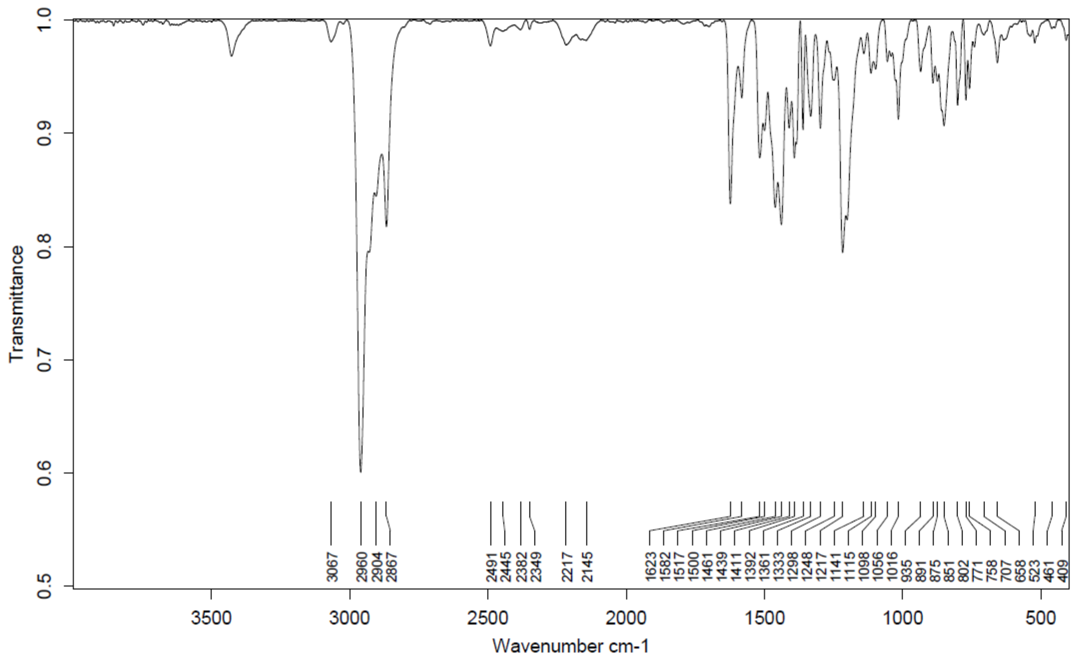

Fig. S 20: IR spectrum (ATR) of [(NON)Dy(BH_4)(thf)] (**3-Dy**).

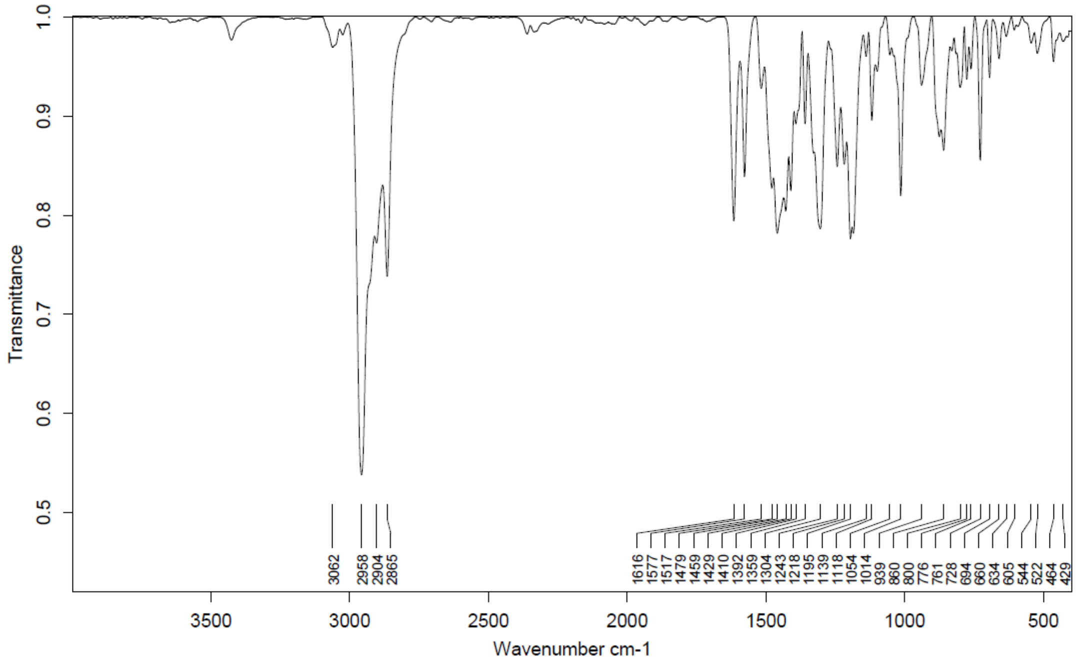

Fig. S 21: IR spectrum (ATR) of [K{(NON)Y(thf)}$_2$(μ_3-η^3:η^3:η^2-P_3)] (**4-Y**).

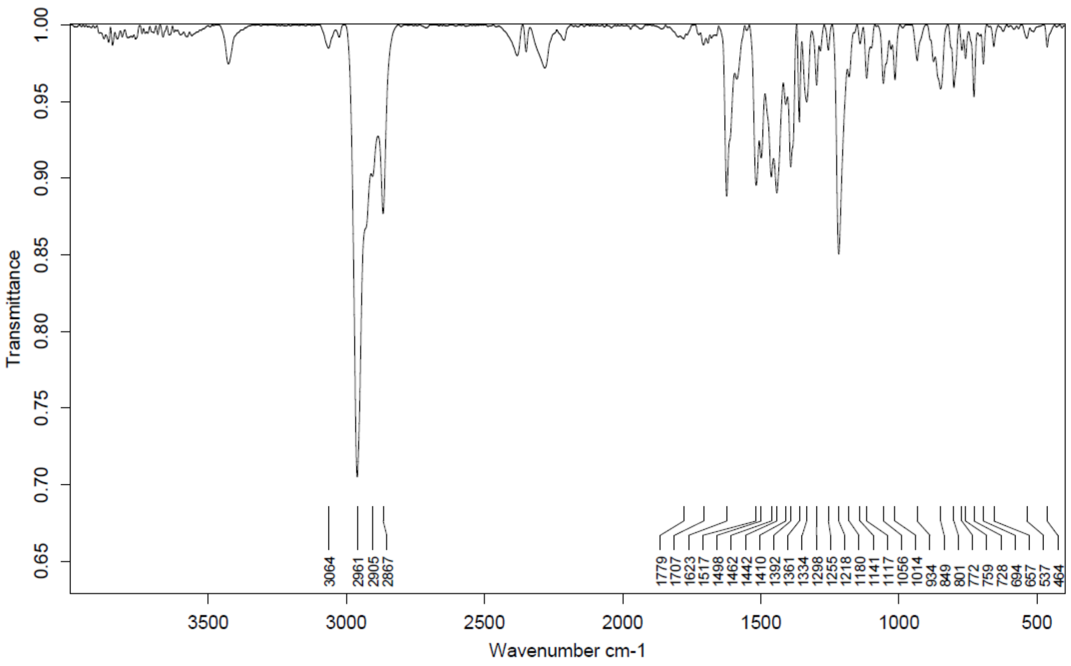

Fig. S 22: IR spectrum (ATR) of [K{(NON)Sm(thf)}$_2$(μ_3-η^3:η^3:η^2-P_3)] (**4-Sm**).

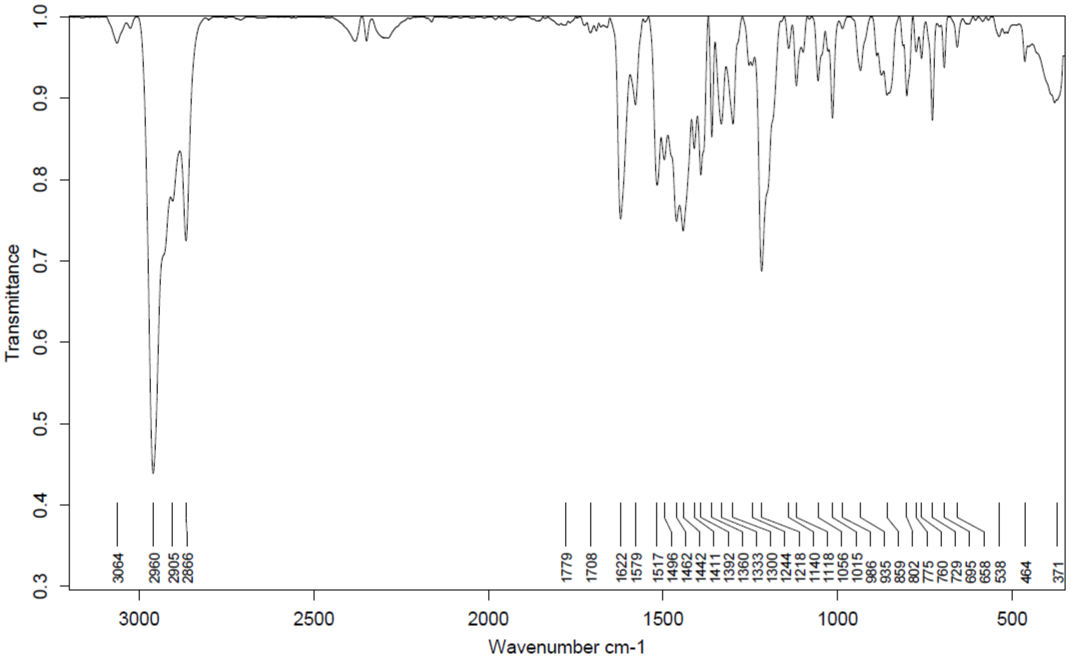

Fig. S 23: IR spectrum (ATR) of [K{(NON)Dy(thf)}$_2$(μ_3-η^3:η^3:η^2-P_3)] (**4-Dy**).

5. X-ray Crystallographic Studies

Suitable crystals were selected under an optic microscope equipped with polarizing filters, covered in mineral oil (Aldrich) and mounted on a MiTeGen holder. Subsequently, the crystals were directly transferred to the cold stream of a *STOE IPDS 2* (150 K) or *STOE StadiVari* (100 K) diffractometer, equipped with a *Mo-sealed tube*, a *MoGenix 3D HF* or a *Ga-MetalJet* X-ray source (Mo-Kα: ʎ = 0.71073 Å, Ga-Kα: ʎ = 1.34143 Å). All structures were solved using the programs SHELXS/T and Olex2 1.3.[5, 6, 7] The remaining non-hydrogen atoms were located from successive difference Fourier map calculations. The refinements were carried out by using full-matrix least-squares techniques on F^2 by using the program SHELXL. In each case, the locations of the largest peaks in the final difference Fourier map calculations, as well as the magnitude of the residual electron densities, were of no chemical significance. Specific comments on the structures discussed here are given below.

[(NON)Sm(thf)$_2$] (**1-Sm**): Crystals of compound **1-Sm** showed positional disorder of one *iso*-propyl moiety of one Dipp-group. To model the disordered moiety, SIMU and SADI constraints were applied.

[(NON)Yb(thf)$_2$] (**1-Yb**): Crystals of compound **1-Sm** showed positional disorder of one *iso*-propyl moiety of one Dipp-group. To model the disordered moiety, SIMU and SADI constraints were applied.

[{(NON)Sm(thf)$_2$}$_2$(μ-η^4:η^4-P$_4$)] (**2-Sm**): The asymmetric unit of compound **2-Sm** contains one molecule of toluene with a chemical occupancy of 25% on a special position. This toluene was modelled using negative PART instructions and SIMU restraints.

[{(NON)Yb(thf)$_2$}$_2$(μ-η^4:η^4-P$_4$)] (**2-Yb**): The asymmetric unit of compound **2-Yb** contains one molecule of THF with a chemical occupancy of 25% on a special position. This THF molecule was modelled with *FragmentDB*[8, 9] and moderate SIMU and RIGU restraints.

Alert level B:

Check Calcd Resid. Dens. 0.96Ang From Yb1 2.62 eA-3

Author Response: Fourier truncation error.

[K{(NON)Y(thf)}$_2$(μ_3-η^3:η^3:η^2-P$_3$)] (**4-Y**): The asymmetric unit of compound **4-Y** contains one molecule of toluene. One *tert*-butyl group of the xanthene backbone, one *iso*-propyl group of the Dipp moiety and the coordinated THF showed positional disorder. This was modelled using moderate SIMU and SADI restraints.

Alert level B:

Check Calcd Resid. Dens. 1.21Ang From P1 3.25 eA-3

Author Response: The residual electron density in the middle of the [P$_3$]$^{3-}$ moiety is probably not correctly assigned during the refinement of the molecular structure due to the use of rigid spherical form factors (ShelXL) for the phosphorus atoms.

[K{(NON)Sm(thf)}$_2$(μ_3-η^3:η^3:η^2-P$_3$)] (**4-Sm**): The asymmetric unit of compound **4-Sm** contains one molecule of toluene. The coordinated THF molecule, one *tert*-butyl group of the xanthene ligand and

one *iso*-propyl group show a positional disorder, which was modelled using moderate SIMU and SADI restraints.

[K{(NON)Dy(thf)}$_2$(μ_3-η^3:η^3:η^2-P_3)] (**4-Dy**): The asymmetric unit of compound **Dy** contains one molecule of toluene. The coordinated THF molecule shows a positional disorder, which was modelled using moderate SIMU restraints. The disorder of one *iso*-propyl group of the Dipp moiety and one *tert*-butyl-group of the xanthene backbone was modelled using SIMU and SADI restraints.

Crystallographic data for the structures reported in this paper have been deposited with the Cambridge Crystallographic Data Centre as a supplementary publication no. 2224069-2224077, and 2235392. Copies of the data can be obtained free of charge on application to CCDC, 12 Union Road, Cambridge CB21EZ, UK (fax: (+(44)1223-336-033; email: deposit@ccdc.cam.ac.uk).

Table S 1: Crystal data and structure refinement.

**Compound**	**1-Sm**	**1-Yb**	**2-Sm**	**2-Yb**
Formula	$C_{55}H_{78}N_2O_3Sm$	$C_{55}H_{78}N_2O_3Yb$	$C_{117}H_{164}N_4O_6P_4Sm_2$	$C_{114}H_{164}N_4O_7P_4Yb_2$
$D_{calc.}$/ g cm^{-3}	1.322	1.317	1.249	1.291
μ/mm^{-1}	1.255	1.920	1.126	1.773
Formula Weight	965.54	988.23	2147.09	2172.44
Colour	clear dark black	orange	red	clear brownish orange
Shape	block	rod	prism	plate-shaped
Size/mm^3	0.21×0.14×0.07	0.12×0.07×0.03	0.13×0.10×0.06	0.13×0.10×0.05
T/K	110	100	100	100
Crystal System	monoclinic	orthorhombic	orthorhombic	orthorhombic
Space Group	$P2_1$	*Pbca*	*Pban*	*Pban*
a/Å	13.5817(8)	18.5124(14)	32.7745(7)	32.1927(9)
b/Å	14.3505(6)	15.2519(11)	12.3366(3)	12.2887(4)
c/Å	13.9019(8)	35.298(3)	14.1237(5)	14.1317(6)
α/°	-	-	-	-
β/°	116.465(4)	-	-	-
γ/°	-	-	-	-
V/Å^3	2425.6(2)	9966.5(13)	5710.6(3)	5590.6(3)
Z	2	8	2	2
Z'	1	1	0.25	0.25
Wavelength/Å	0.71073	0.71073	0.71073	0.71073
Radiation type	Mo K_α	Mo K_α	Mo K_α	Mo K_α
Q_{min}/°	2.166	2.200	2.278	2.903
Q_{max}/°	31.822	29.520	29.583	27.760
Measured Refl.	65099	31881	109804	32524
Independent Refl.	14294	12120	7708	6352
Reflections with I≥2 σ(I)	11804	7910	4824	3731
R_{int}	0.0466	0.0392	0.0698	0.0393
Parameters	595	595	344	340
Restraints	29	35	42	66
Largest Peak	2.264	2.449	1.101	2.592
Deepest Hole	-2.964	-1.739	-1.543	-1.371
GooF	1.238	1.078	1.094	0.918
wR_2 (all data)	0.1317	0.1534	0.1729	0.1175
wR_2	0.0888	0.1330	0.1387	0.1106
R_1 (all data)	0.0790	0.1134	0.0962	0.0700
R_1	0.0438	0.0647	0.0545	0.0413
Flack Parameter	-0.040(6)	-	-	-

Compound	3-Y	3-Sm	3-Dy	4-Y
Formula	$C_{55}H_{82}BN_2O_3Y$	$C_{55}H_{82}BN_2O_3Sm$	$C_{55}H_{82}BN_2O_3Dy$	$C_{116}H_{156}KN_4O_4P_3Y_2$
$D_{calc.}$/ g cm^{-3}	1.183	1.245	1.263	1.202
μ/mm^{-1}	1.173	1.164	1.473	1.706
Formula Weight	918.94	980.38	992.53	1980.27
Colour	colourless	orange	green	colourless
Shape	block	block	fragment	needle
Size/mm^3	0.18×0.14×0.07	0.63×0.53×0.37	0.39×0.35×0.33	0.21×0.08×0.02
T/K	100	150	150	180
Crystal System	monoclinic	monoclinic	monoclinic	monoclinic
Space Group	$P2_1/c$	$P2_1/c$	$P2_1/c$	$C2/c$
a/Å	13.7490(9)	13.8154(4)	13.7970(3)	31.5819(6)
b/Å	14.0841(6)	14.2166(3)	14.1608(5)	24.1585(4)
c/Å	26.950(2)	26.9732(7)	27.0491(6)	16.5122(3)
α/°	-	-	-	-
β/°	98.698(5)	99.004(2)	98.959(2)	119.7260(10)
γ/°	-	-	-	-
V/Å^3	5158.7(5)	5232.5(2)	5220.3(2)	10940.5(4)
Z	4	4	4	4
Z'	1	1	1	0.5
Wavelength/Å	0.71073	0.71073	0.71073	1.34143
Radiation type	Mo K_α	Mo K_α	Mo K_α	Ga K_α
Q_{min}/°	1.972	1.492	1.628	2.821
Q_{max}/°	30.338	25.650	25.694	64.079
Measured Refl.	30771	31394	30289	34539
Independent Refl.	13149	9858	9793	12995
Reflections with I≥2 σ(I)	9559	8552	7754	9421
R_{int}	0.0443	0.0177	0.0279	0.0253
Parameters	591	591	591	673
Restraints	0	0	0	181
Largest Peak	0.604	0.389	0.438	2.835
Deepest Hole	-0.924	-0.504	-0.571	-1.438
GooF	1.014	1.038	1.017	1.056
wR_2 (all data)	0.1378	0.0617	0.0665	0.2127
wR_2	0.1193	0.0591	0.0622	0.1946
R_1 (all data)	0.0859	0.0312	0.0418	0.0867
R_1	0.0526	0.0242	0.0267	0.0646
Flack Parameter	-	-	-	-

Compound	4-Sm	4-Dy
Formula	$C_{116}H_{156}KN_4O_4P_3Sm_2$	$C_{116}H_{156}KN_4O_4P_3Dy_2$
$D_{calc.}$/ g cm^{-3}	1.305	1.331
μ/mm^{-1}	1.222	1.534
Formula Weight	2103.15	2127.45
Colour	red	yellow
Shape	block	plate
Size/mm^3	0.20×0.11×0.06	0.14×0.10×0.06
T/K	100	100
Crystal System	monoclinic	monoclinic
Space Group	*C*2/*c*	*C*2/*c*
a/Å	31.686(2)	31.3677(14)
b/Å	23.6758(8)	23.787(2)
c/Å	16.4497(9)	16.3094(7)
α/°	-	-
β/°	119.805(4)	119.275(3)
γ/°	-	-
V/Å^3	10708.1(10)	10615.3(10)
Z	4	4
Z'	0.5	0.5
Wavelength/Å	0.71073	0.71073
Radiation type	Mo K_α	Mo K_α
Q_{min}/°	2.092	2.092
Q_{max}/°	31.795	31.803
Measured Refl.	54911	33310
Independent Refl.	14738	13913
Reflections with I≥2 σ(I)	8998	10025
R_{int}	0.0539	0.0321
Parameters	689	680
Restraints	63	29
Largest Peak	2.029	1.381
Deepest Hole	-1.943	-1.838
GooF	1.032	1.027
wR_2 (all data)	0.1596	0.1124
wR_2	0.1232	0.0963
R_1 (all data)	0.1240	0.0719
R_1	0.0593	0.0414
Flack Parameter	-	-

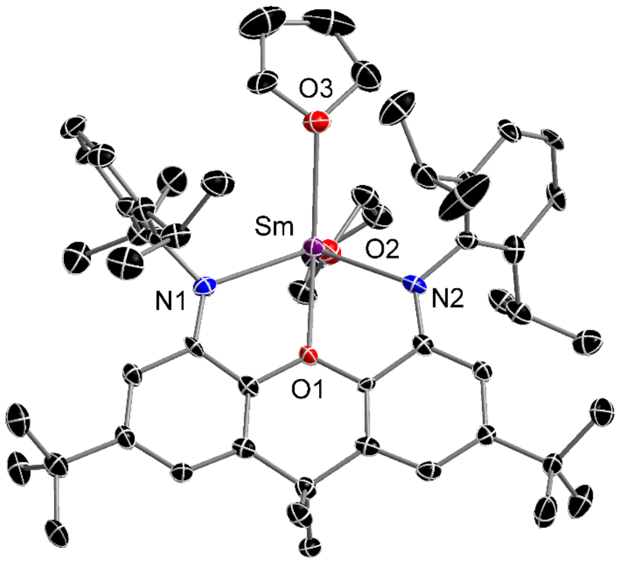

Fig. S 24: Molecular structure of **1-Sm** in the solid state. Thermal ellipsoids are represented at 50% probability. Hydrogen atoms are omitted for clarity. Only one part of the disordered *iso*-propyl group of the Dipp moiety is depicted. For selected bond lengths and angles see table S2. Colour code: Sm (purple), N (blue), O (red), C (black).

Table S 2: Selected bond lengths and angles for [(NON)Sm(thf)$_2$] **1-Sm**.

Length/ Å			Angle/°			
Sm	N1	2.418(7)	N1	Sm	N2	122.8(2)
Sm	N2	2.433(7)	N1	Sm	O1	62.7(2)
Sm	O1	2.554(5)	N1	Sm	O3	109.7(2)
Sm	O2	2.564(6)	N2	Sm	O3	112.1(2)
Sm	O3	2.526(6)	N2	Sm	O1	63.0(2)
			O3	Sm	O2	90.6(2)
			O3	Sm	O1	120.2(2)

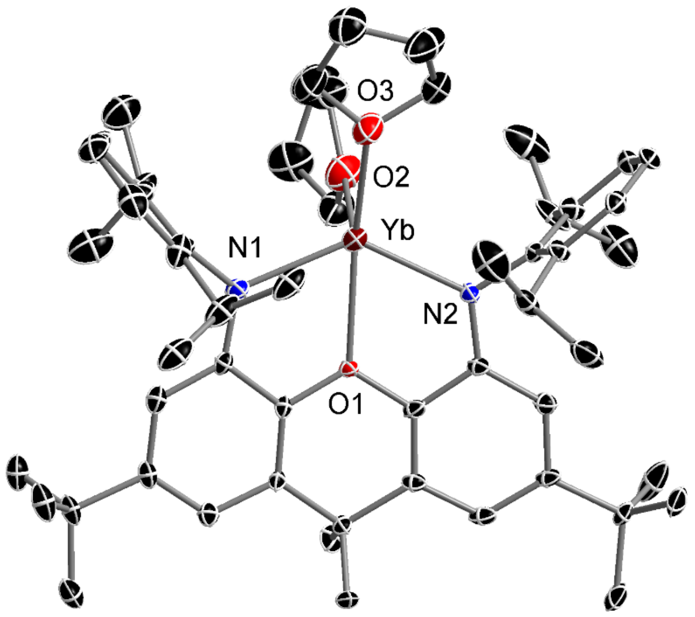

Fig. S 25: Molecular structure of **1-Yb** in the solid state. Thermal ellipsoids are represented at 50% probability. Hydrogen atoms are omitted for clarity. Only one part of the disordered *iso*-propyl group of the Dipp moiety is depicted. For selected bond lengths and angles see table S3. Colour code: Yb (dark red), N (blue), O (red), C (black).

Table S 3: Selected bond lengths and angles for [(NON)Yb(thf)$_2$] **1-Yb**.

Length/ Å			Angle/°			
Yb	N1	2.344(5)	N1	Yb	N2	132.0(2)
Yb	N2	2.315(5)	N1	Yb	O1	66.7(2)
Yb	O1	2.402(4)	N1	Yb	O3	111.1(2)
Yb	O2	2.336(6)	N2	Yb	O3	107.1(2)
Yb	O3	2.355(5)	N2	Yb	O1	67.4(2)
			O3	Yb	O2	90.6(2)
			O3	Yb	O1	160.3(2)

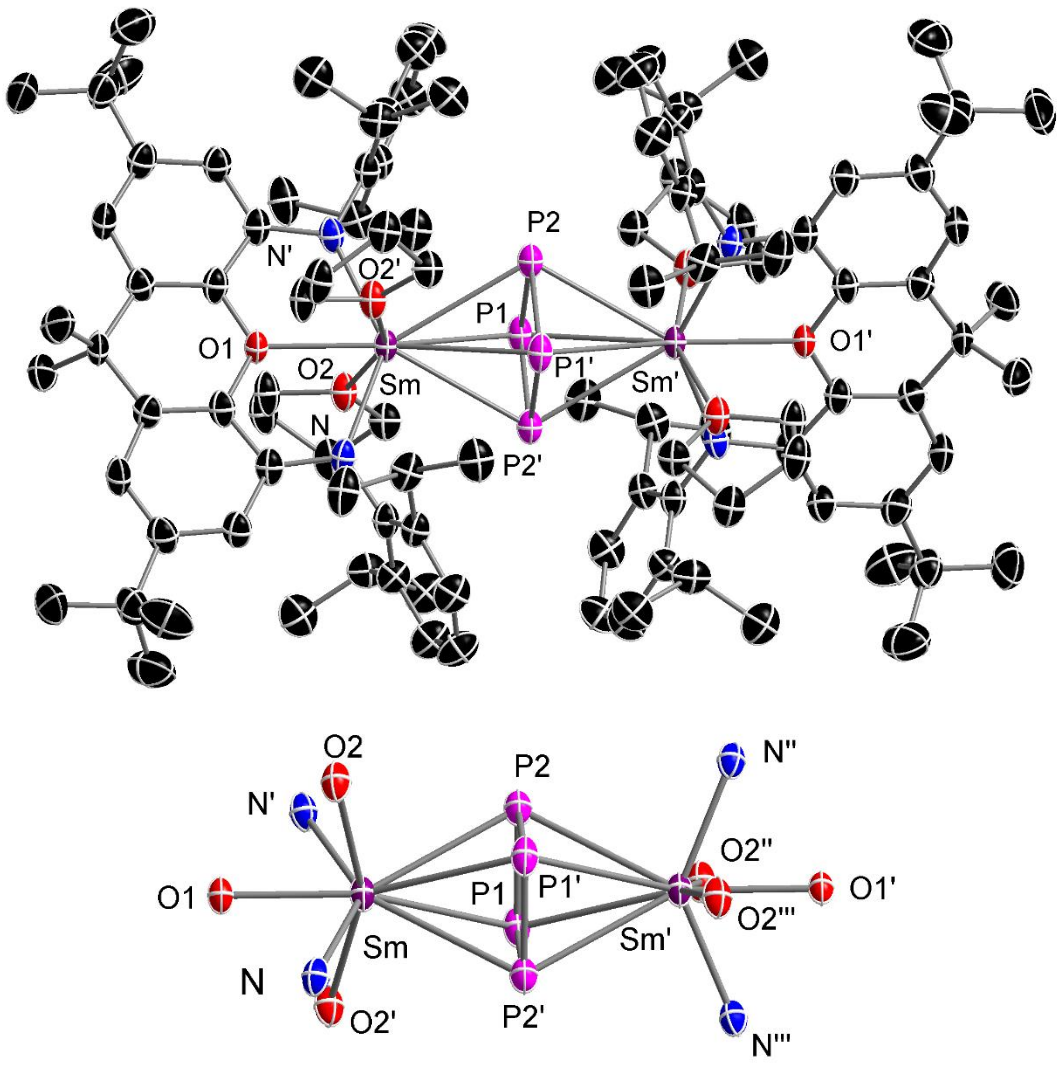

Fig. S 26: Top: Molecular structure of **2-Sm** in the solid state. Thermal ellipsoids are represented at 50% probability. Hydrogen atoms and one molecule of toluene are omitted for clarity. Bottom: Simplified depiction of the central structure motif. For selected bond lengths and angles see table S4. Colour code: Sm (purple), N (blue), O (red), P (pink), C (black).

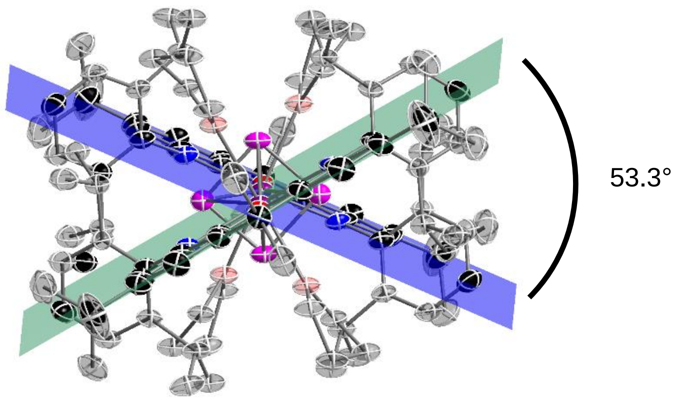

Fig. S 27 Side view of the molecular structure in the solid state of **2-Sm**. Illustration of twisting of the two xanthene backbones (green and blue plane).

Table S 4: Selected bond lengths and angles for [{(NON)Sm(thf)$_2$}$_2$(μ-η^4:η^4-P_4)] **2-Sm**. Ct = Centroid of the subscript ring moiety.

Length/ Å			Angle/°			
Sm	P1	3.0727(8)	P1	P2	P1’	88.62(9)
Sm	P2	3.0908(10)	P2	P1	P2’	91.38(9)
Sm	Ct_{P4}	2.6787(7)	P1	Sm	P1’	58.68(5)
Sm	N	2.360(4)	P1	Sm	P2	40.93(3)
Sm	O1	2.408(4)	P2	Sm	P2’	59.86(6)
Sm	O2	2.523(3)	N	Sm	N’	135.1(2)
P1	P2	2.155(2)	O1	Sm	Ct_{P4}	179.99

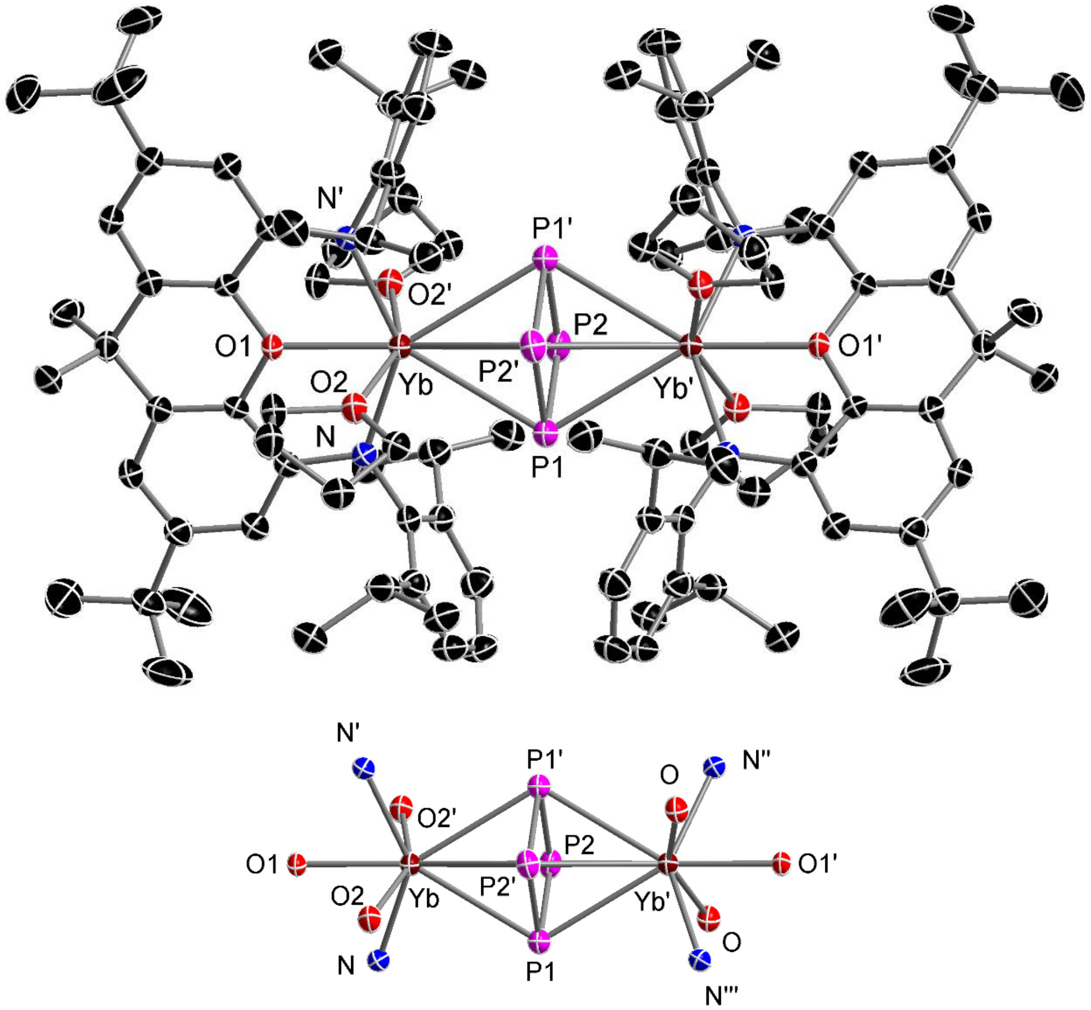

Fig. S 28: Top: Molecular structure of **2-Yb** in the solid state. Thermal ellipsoids are represented at 50% probability. Hydrogen atoms and one molecule of THF are omitted for clarity. Only one part of the symmetry generated, disordered molecule is shown. Bottom: Simplified depiction of the central structure motif. For selected bond lengths and angles see table S5. Colour code: Yb (dark red), N (blue), O (red), P (pink), C (black).

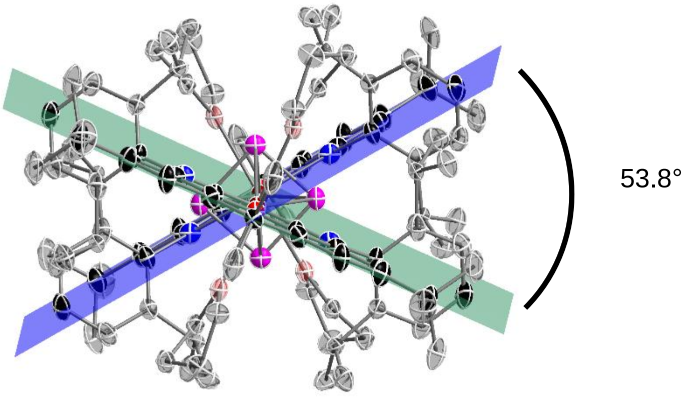

Fig. S 29: Side view of the molecular structure in the solid state of **2-Yb**. Illustration of twisting of the two xanthene backbones (green and blue plane).

Table S 5: Selected bond lengths and angles for [{(NON)Yb(thf)$_2$}$_2$(μ-η^4:η^4-P_4)] **2-Yb**. Ct = Centroid of the subscript ring moiety.

Length/ Å			Angle/°			
Yb	P1	3.0192(8)	P1	P2	P1’	91.27(8)
Yb	P2	3.0021(8))	P2	P1	P2’	88.73(8)
Yb	Ct_{P4}	2.5960(6)	P1	Yb	P1’	61.40(5)
Yb	N	2.290(3)	P1	Yb	P2	41.97(2)
Yb	O1	2.296(3)	P2	Yb	P2’	60.29(5)
Yb	O2	2.442(3)	N	Yb	N’	139.4(2)
P1	P2	2.156(2)	O1	Yb	Ct_{P4}	180.00

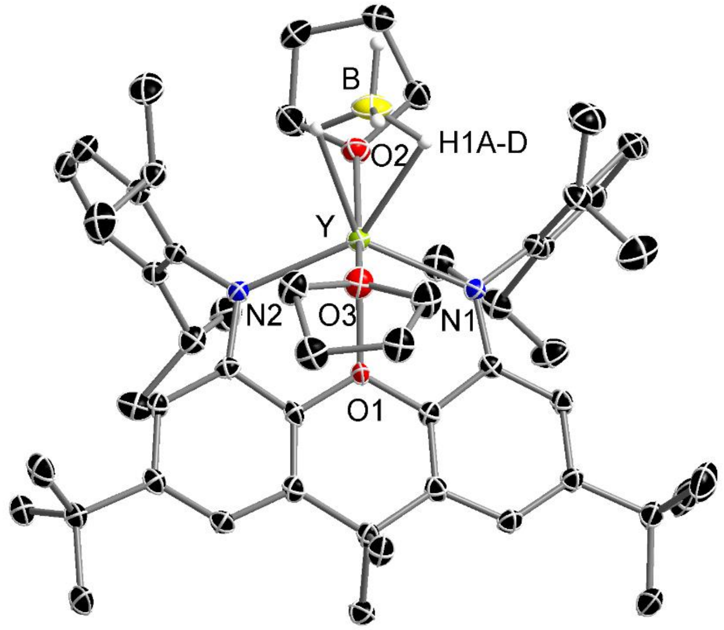

Fig. S 30: Molecular structure of **3-Y** in the solid state. Thermal ellipsoids are represented at 50% probability. Hydrogen atoms, except the freely refined hydridic ones, are omitted for clarity. For selected bond lengths and angles see table S6. Colour code: Y (green), N (blue), O (red), C (black), H (grey).

Table S 6: Selected bond lengths and angles for [(NON)Y(BH_4)(thf)$_2$] **3-Y**.

Length/ Å			Angle/°			
Y	N1	2.280(2)	N1	Y	N2	129.13(8)
Y	N2	2.299(2)	N1	Y	B	112.68(10)
Y	O1	2.337(2)	O1	Y	O2	130.79(7)
Y	O2	2.356(2)	O1	Y	O3	71.77(7)
Y	O3	2.360(2)	N1	Y	O1	66.96(7)
Y	B	2.717(3)	N2	Y	O1	67.29(7)
Y	H1A	2.26(3)				
Y	H1B	2.26(3)				

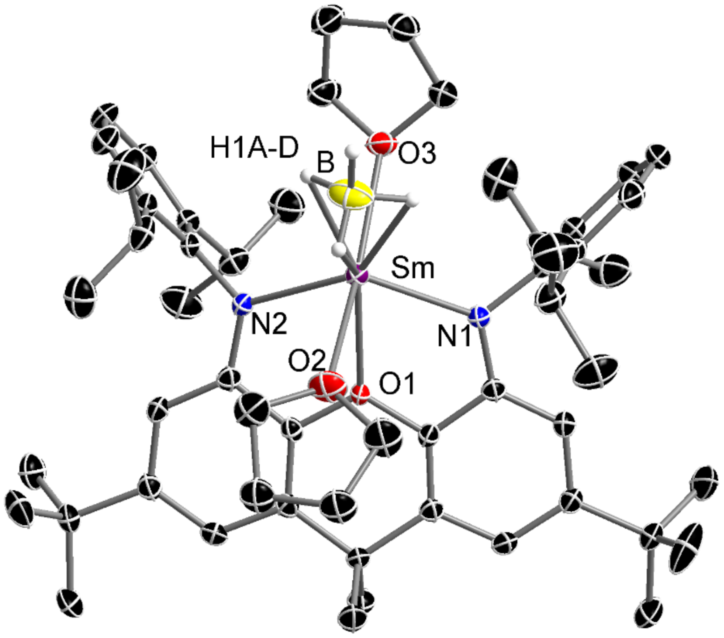

Fig. S 31: Molecular structure of **3-Sm** in the solid state. Thermal ellipsoids are represented at 50% probability. Hydrogen atoms, except the freely refined hydridic ones, are omitted for clarity. For selected bond lengths and angles see table S7. Colour code: Sm (purple), N (blue), O (red), C (black), H (grey).

Table S 7: Selected bond lengths and angles for [(NON)Sm(BH_4)($thf)_2$] **3-Sm**.

Length/ Å			Angle/°			
Sm	N1	2.325(2)	N1	Sm	N2	125.35(6)
Sm	N2	2.346(2)	N1	Sm	B	115.43(10)
Sm	O1	2.440(2)	O1	Sm	O2	69.15(6)
Sm	O2	2.477(2)	O1	Sm	O3	125.35(5)
Sm	O3	2.477(2)	N1	Sm	O1	64.72(5)
Sm	B	2.671(3)	N2	Sm	O1	65.06(5)
Sm	H1A	2.41(3)				
Sm	H1B	2.49(3)				
Sm	H1D	2.60(4)				

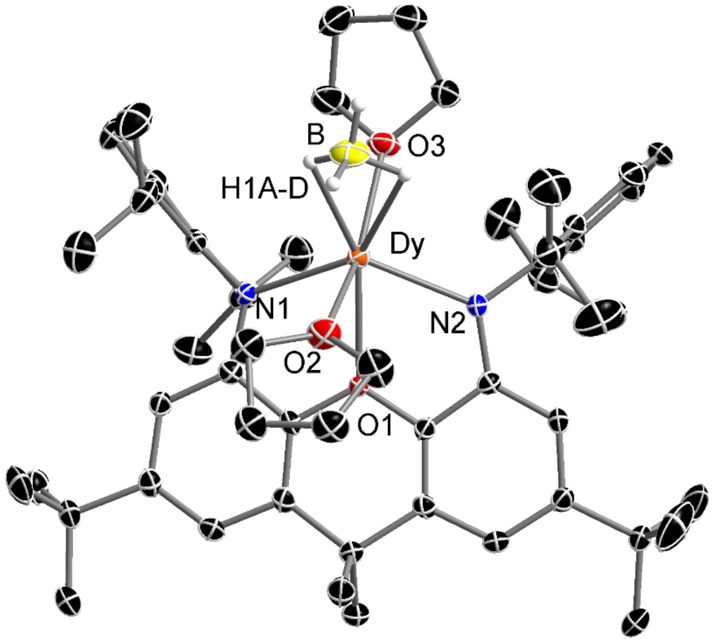

Fig. S 32: Molecular structure of **3-Dy** in the solid state. Thermal ellipsoids are represented at 50% probability. Hydrogen atoms, except the freely refined hydridic ones, are omitted for clarity. For selected bond lengths and angles see table S8. Colour code: Dy (orange), N (blue), O (red), C (black), H (grey).

Table S 8: Selected bond lengths and angles for [(NON)Dy(BH_4)($thf)_2$] **3-Dy**.

Length/ Å			Angle/°			
Dy	N1	2.305(2)	N1	Dy	N2	129.00(8)
Dy	N2	2.288(2)	N1	Dy	B	117.61(12)
Dy	O1	2.363(2)	O1	Dy	O2	71.39(7)
Dy	O2	2.393(2)	O1	Dy	O3	130.37(7)
Dy	O3	2.387(2)	N1	Dy	O1	67.18(7)
Dy	B	2.707(4)	N2	Dy	O1	66.53(7)
Dy	H1A	2.25(3)				
Dy	H1B	2.38(5)				

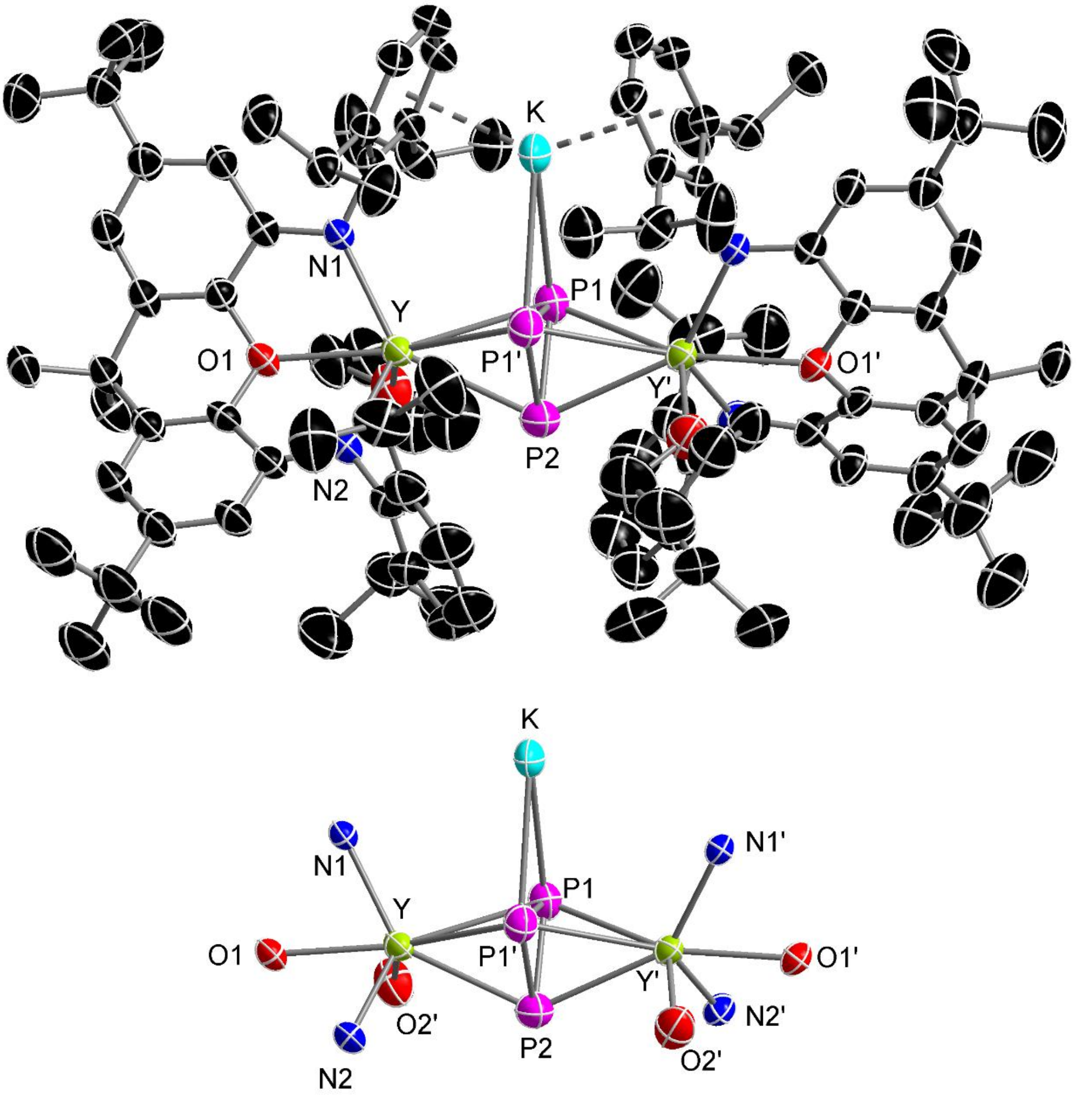

Fig. S 33: Top: Molecular structure of **4-Y** in the solid state. Thermal ellipsoids are represented at 50% probability. Hydrogen atoms and one molecule of toluene are omitted for clarity. Only one part of the disordered THF molecule, *tert*-butyl groups and *iso*-propyl groups is shown. Bottom: Simplified depiction of the central structure motif. For selected bond lengths and angles see table S9. Colour code: Y (green), N (blue), O (red), P (pink), K (turquoise), C (black).

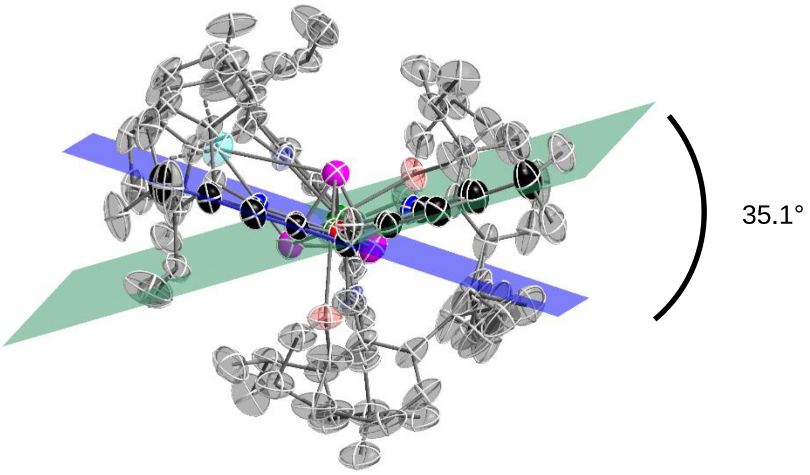

Fig. S 34: Side view of the molecular structure in the solid state of **4-Y**. Illustration of folding of the xanthene backbone along Y1-O1-C7 axis (green and blue plane).

Table S 9: Selected bond lengths and angles for [K{(NON)Y(thf)}$_2$(μ_3-η^3:η^3:η^2-P_3)] **4-Y**. Ct = Centroid of the subscript ring moiety.

Length/ Å			Angle/°			
Y	P1	2.8658(11)	P1	P2	P1'	61.49(5)
Y	P2	2.8485(8)	P2	P1'	P1	59.25(4)
Y	Ct$_{P_3}$	2.5876(7)	P1	K	P1'	41.36(3)
Y	O1	2.424(2)	P1	Y	P2	45.04(3)
Y	O2	2.316(3)	N1	Y	N2	122.76(10)
Y	N1	2.351(3)				
Y	N2	2.301(3)				
P1	P2	2.188(2)				
P1	P1'	2.238(2)				
K	P1	3.168(2)				
K	Ct$_{phenyl}$1	2.7813(5)				

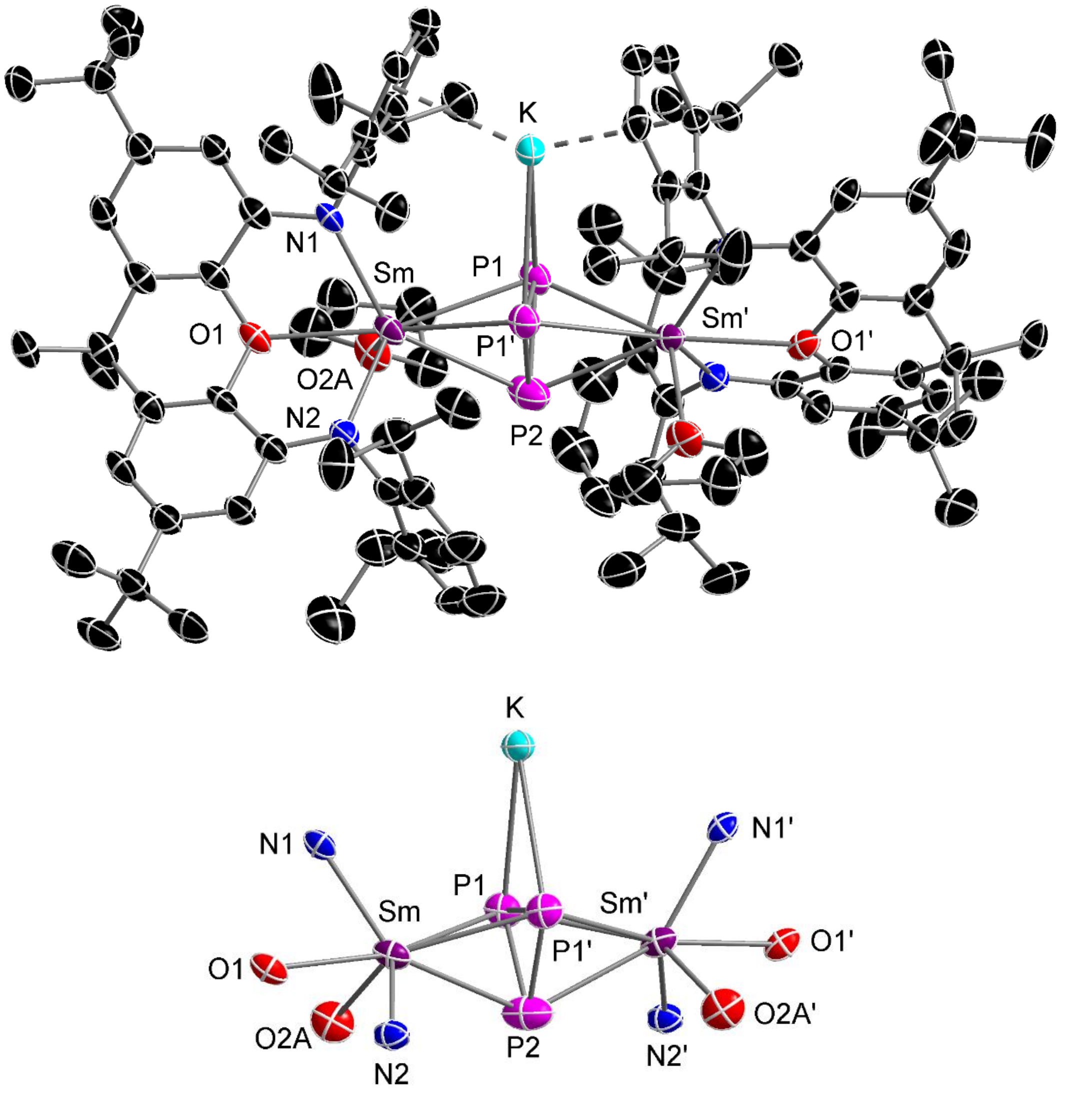

Fig. S 35: Top: Molecular structure of **4-Sm** in the solid state. Thermal ellipsoids are represented at 50% probability. Hydrogen atoms and one molecule of toluene are omitted for clarity. Only one part of the disordered THF molecule, *tert*-butyl groups and *iso*-propyl groups is shown. Bottom: Simplified depiction of the central structure motif. For selected bond lengths and angles see table S10. Colour code: Sm (purple), N (blue), O (red), P (pink), K (turquoise), C (black).

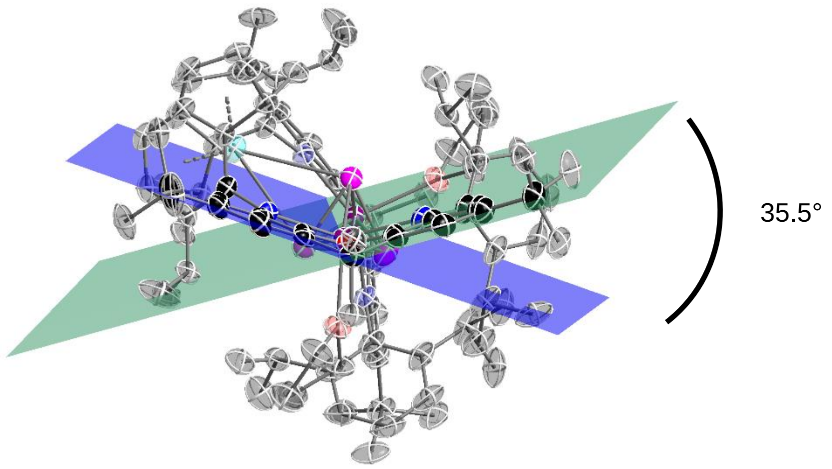

Fig. S 36: Side view of the molecular structure in the solid state of **4-Sm**. Illustration of folding of the xanthene backbone along Sm1-O1-C7 axis (green and blue plane).

Table S 10: Selected bond lengths and angles for [K{(NON)Sm(thf)}$_2$(μ_3-η^3:η^3:η^2-P_3)] **4-Sm**. Ct = Centroid of the subscript ring moiety.

Length/ Å			Angle/°			
Sm	P1	2.878(2)	P1	P2	P1'	61.66(5)
Sm	P2	2.8557(10)	P2	P1'	P1	59.16(5)
Sm	CtP_3	2.6030(7)	P1	K	P1'	40.84(6)
Sm	O1	2.500(4)	P1	Sm	P2	44.38(5)
Sm	O2A	2.367(12)	N1	Sm	N2	120.5(2)
Sm	N1	2.373(4)				
Sm	N2	2.337(4)				
P1	P2	2.166(3)				
P1	P1'	2.220(3)				
K	P1	3.182(2)				
K	$Ct_{phenyl}1$	2.8112(6)				

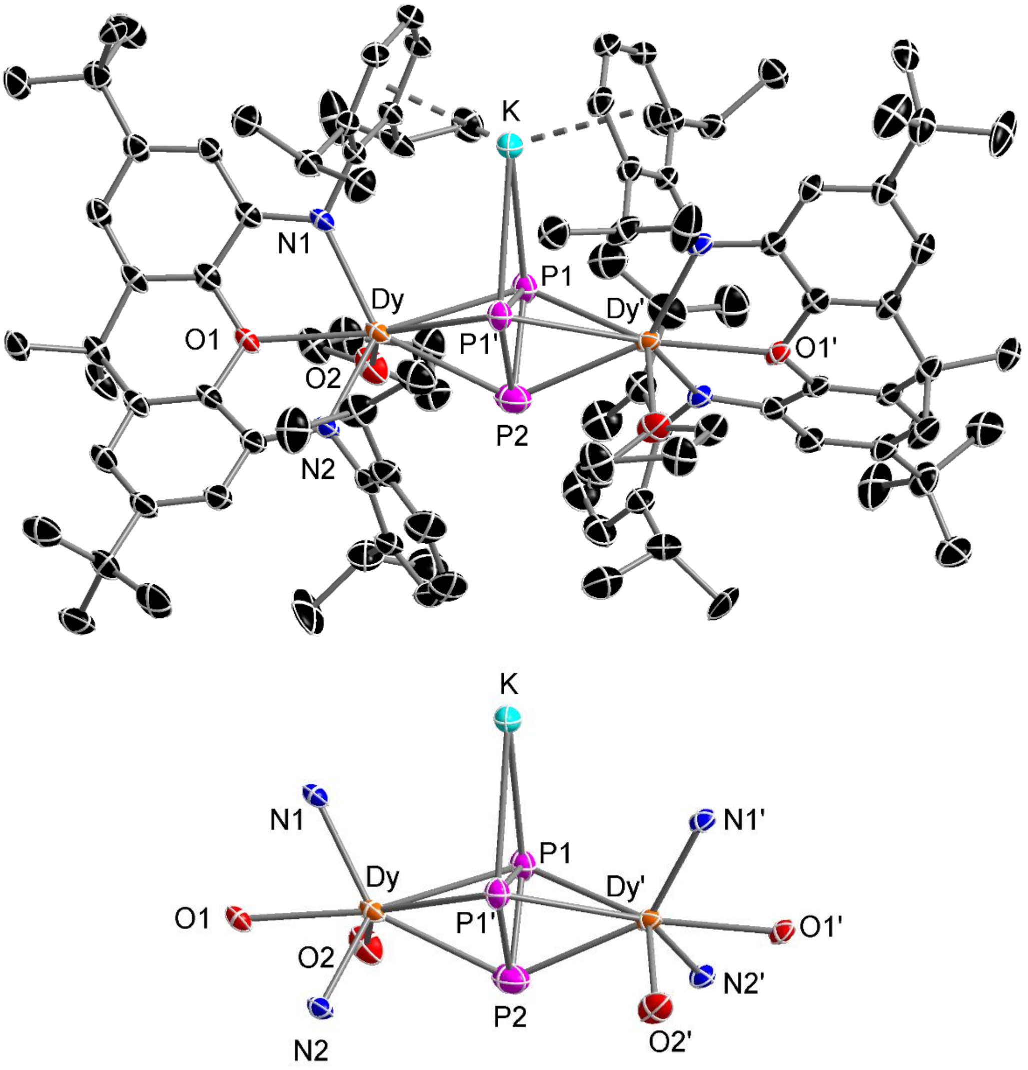

Fig. S 37: Top: Molecular structure of **4-Dy** in the solid state. Thermal ellipsoids are represented at 50% probability. Hydrogen atoms and one molecule of toluene are omitted for clarity. Only one part of the disordered THF molecule, *tert*-butyl groups and *iso*-propyl groups is shown. Bottom: Simplified depiction of the central structure motif. For selected bond lengths and angles see table S11. Colour code: Dy (orange), N (blue), O (red), P (pink), K (turquoise), C (black).

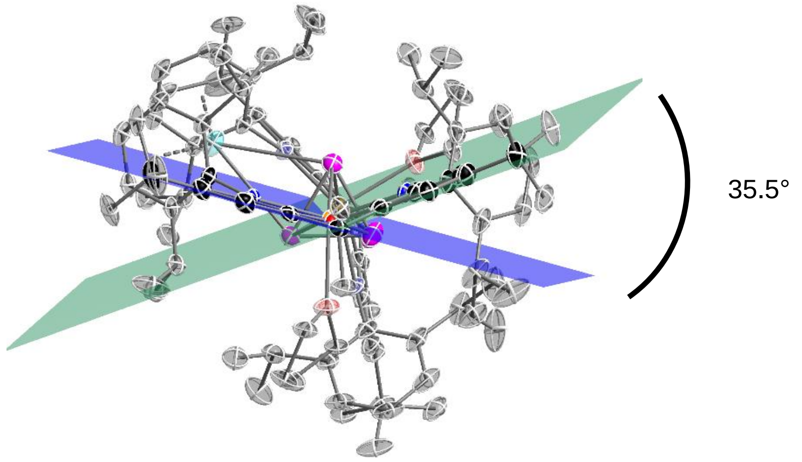

Fig. S 38: Side view of the molecular structure in the solid state of **4-Dy**. Illustration of folding of the xanthene backbone along Dy1-O1-C7 axis (green and blue plane).

Table S 11: Selected bond lengths and angles for [K{(NON)Dy(thf)}$_2$(μ_3-η^3:η^3:η^2-P_3)] **4-Dy**. Ct = Centroid of the subscript ring moiety.

Length/ Å			Angle/°			
Dy	P1	2.8251(11)	P1	P2	P1′	62.04(4)
Dy	P2	2.8025(8)	P2	P1′	P1	58.97(4)
Dy	CtP_3	2.5446(7)	P1	K	P1′	40.81(3)
Dy	O1	2.444(2)	P1	Dy	P2	44.58(3)
Dy	O2	2.358(3)	N1	Dy	N2	122.52(10)
Dy	N1	2.334(3)				
Dy	N2	2.289(3)				
P1	P2	2.160(2)				
P1	P1′	2.227(2)				
K	P1	3.193(2)				
K	Ct_{phenyl}1	2.7727(4)				

6. Magnetism

Ab initio [9,7]-CASSCF/RASSI-SO/SINGLE_ANISO type calculations were performed using the OpenMolcas Package.[10] The input structure was modified as described in the main article but otherwise used as obtained from crystal structure refinement. 21 sextets, 128 quartets and 130 doublets were taken into account for the RASSI routine of the calculation. The applied basis sets were the relativistic ANO-RCC sets taken from the Molcas library. For Dy and P valence triple zeta with polarisation, for Y, O, N, K and the C's coordinating towards K valence double zeta with polarisation and for the remaining C's and H's valence double zeta was chosen. The simulation of the molar susceptibility and magnetisation data was performed using the PHI program package.[11]

Magnetic measurements were carried out on a polycrystalline sample of **4-Dy** mixed with eicosane in a flame sealed NMR tube. All data were corrected for the diamagnetic contributions of the sample holder, the eico and the underlying diamagnetism of the compound. T-dependent DC susceptibility and AC data were collected on a QuantumDesign MPMS-XL SQUID magnetometer. Field-dependent magnetisation data up to 7 T and hysteresis loops were collected on a QuantumDesign MPMS3-VSM SQUID magnetometer.

Table S 12: SINGLE_ANISO energies, magnetic axes, g-tensors and wave function compositions of the KD's found for **4-Dy***.

Doublet state	ΔE/cm^{-1}	ΔE/K	Angle between magnetic z-axes/°	g_x	g_y	g_z	Wavefunction composition
1	0	0	-	0.01	0.02	19.64	∓ 96% |15/2› ∓ 4% |11/2›
2	151.00	217.25	1.79	0.36	0.67	15.76	± 78% |13/2› ± 19% |9/2› ± 3% |5/2›
3	238.19	342.70	3.88	1.20	1.57	12.37	∓ 62% |11/2› ∓ 28% |7/2› ∓ 5% |3/2› ∓3% |15/2› ± 1% |1/2› ∓ 1% |5/2›
4	338.70	487.31	4.54	2.08	4.11	9.71	± 41% |9/2› ± 30% |5/2› ± 18% |13/2› ± 6% |1/2› ∓ 3% |11/2› ∓2% |3/2› ± 1% |7/2›
5	435.04	625.92	88.56	8.50	6.04	3.31	∓ 31% |3/2› ∓ 27% |7/2› ∓ 23% |11/2› ± 8% |1/2› ± 7% |9/2› ∓ 3% |5/2› ± 1% |13/2›
6	513.47	738.76	76.91	1.26	2.62	13.80	± 27% |5/2› ∓ 21% |9/2› ∓ 20% |1/2› ± 17% |7/2› ± 8% |3/2› ± 5% |11/2› ∓ 2% |13/2›
7	541.96	779.75	86.93	0.49	1.14	17.21	± 33% |1/2› ∓ 24% |3/2› ± 17% |5/2› ∓ 15% |7/2› ± 7% |9/2› ∓ 3% |11/2› ± 1% |13/2›
8	591.47	850.99	79.78	0.24	0.59	18.72	∓ 31% |3/2› ± 31% |1/2› ∓ 21% |5/2› ∓ 11% |7/2› ∓ 4% |9/2› ∓ 1% |11/2›

Table S 13: *Ab initio* crystal field parameters for **4-Dy***.

k	q	B(k,q)
2	-2	-0.87231155461920E-01
2	-1	-0.14489757842781E+00
2	0	-0.28642508259298E+01
2	1	0.70080123039822E+00
2	2	0.24631566595432E+01
4	-4	0.82622282826377E-02
4	-3	-0.12840255076479E-01
4	-2	0.14831653706290E-01
4	-1	-0.12223682013701E-02
4	0	-0.17267698411221E-02
4	1	-0.39473433684683E-02
4	2	0.18973612338477E-01
4	3	0.85453985470034E-02
4	4	-0.12334723621870E-01
6	-6	-0.56207645717671E-04
6	-5	0.93992492627727E-04
6	-4	0.11085336117894E-03
6	-3	0.60163730488920E-06
6	-2	-0.14503597925889E-04
6	-1	0.24011446844149E-04
6	0	-0.12345103592235E-04
6	1	-0.17151966537439E-04
6	2	-0.15004697316545E-03
6	3	-0.64593431963878E-05
6	4	-0.21531866924044E-04
6	5	0.13490140921388E-03
6	6	0.18494119197718E-04

Table S 14: Best fit parameters using a generalised Debeye model for **4-Dy**.

T	ChiT	ChiS	tau	alpha
2	7.7572	1.7686	0.00108	0.25406
2.2	7.12536	1.62221	0.00107	0.25816
2.4	6.64733	1.51924	0.00106	0.26053
2.6	6.18405	1.41503	0.00105	0.26352
2.8	5.78371	1.33296	0.00105	0.26509
3	5.43696	1.26286	0.00104	0.26622
3.2	5.13258	1.20291	0.00104	0.26738
3.4	4.86309	1.14323	0.00103	0.2696
3.6	4.62369	1.09833	0.00103	0.26964
3.8	4.40624	1.05425	0.00102	0.27042
4	4.20947	1.01397	0.00101	0.27082
4.4	3.8673	0.95223	0.00101	0.26912
4.8	3.5913	0.89717	0.001	0.26925
5.2	3.3461	0.86769	9.93907E-4	0.2632
5.6	3.12933	0.83988	9.65808E-4	0.25253
6	2.94342	0.80236	9.50839E-4	0.24807
6.4	2.7769	0.77806	9.32406E-4	0.23938
6.8	2.62826	0.75337	9.0587E-4	0.22949
7.2	2.49428	0.72916	8.66274E-4	0.21876
7.6	2.37422	0.70628	8.35541E-4	0.20974
8	2.26435	0.68542	7.98193E-4	0.19806
8.4	2.00798	0.63083	6.87643E-4	0.17216
8.8	1.91952	0.61991	6.37504E-4	0.15052
9.2	1.84071	0.58992	5.92227E-4	0.15105

9.6	1.76758	0.57293	5.47302E-4	0.14065
10	1.70036	0.55041	5.07516E-4	0.13773
11	1.5524	0.51101	4.13391E-4	0.11572
12	1.47071	0.48898	3.61709E-4	0.10249
13	1.36347	0.45393	2.97672E-4	0.09307
14	1.27096	0.41516	2.41436E-4	0.0905
15	1.1593	0.38368	1.83195E-4	0.078
16	1.08896	0.34289	1.45308E-4	0.08481
17	1.02987	0.30544	1.15045E-4	0.09302
18	0.97369	0.29638	9.6407E-5	0.08152
19	0.92652	0.25605	7.51725E-5	0.09429
20	0.88224	0.2463	6.28722E-5	0.09003

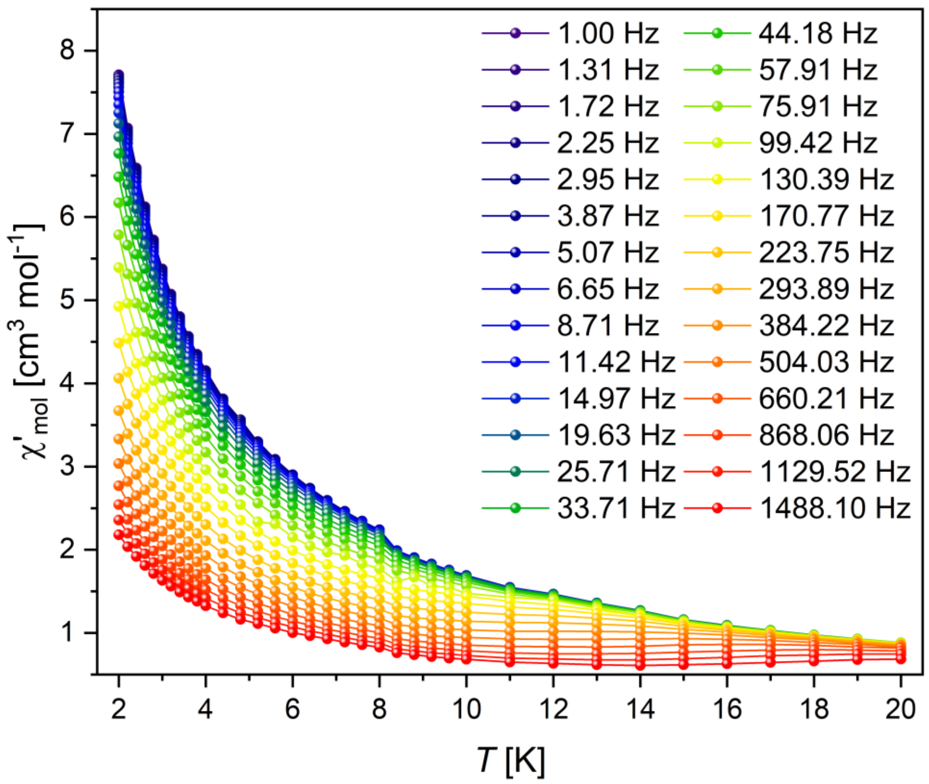

Fig. S 39: In-phase component of the molar magnetic susceptibility vs. temperature at different frequencies for **4-Dy**. Solid lines are a guide for the eye.

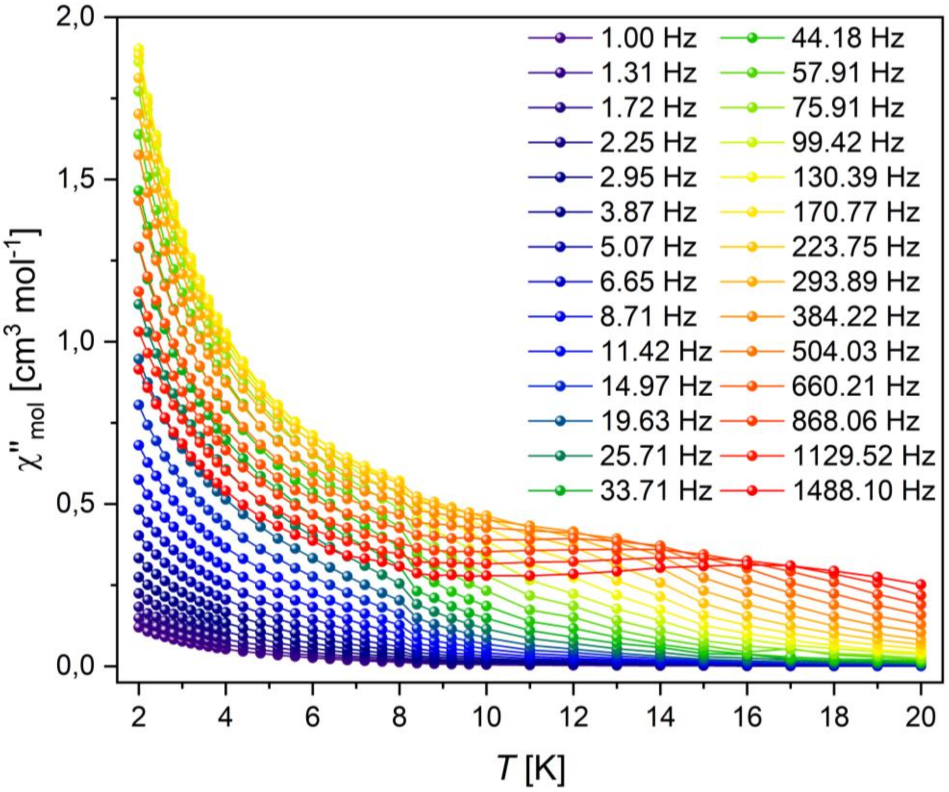

Fig. S 40: Out-of-phase component of the molar magnetic susceptibility vs. temperature at different frequencies for **4-Dy**. Solid lines are a guide for the eye.

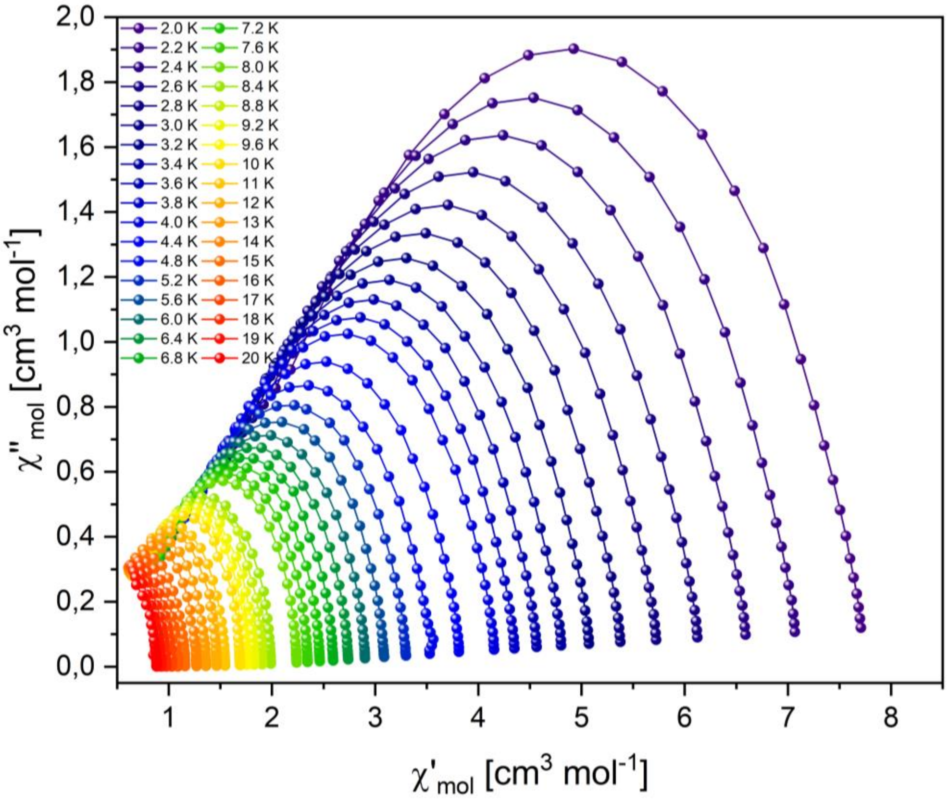

Fig. S 41: Cole-cole plot at different temperatures for **4-Dy**. Solid lines are a guide for the eye.

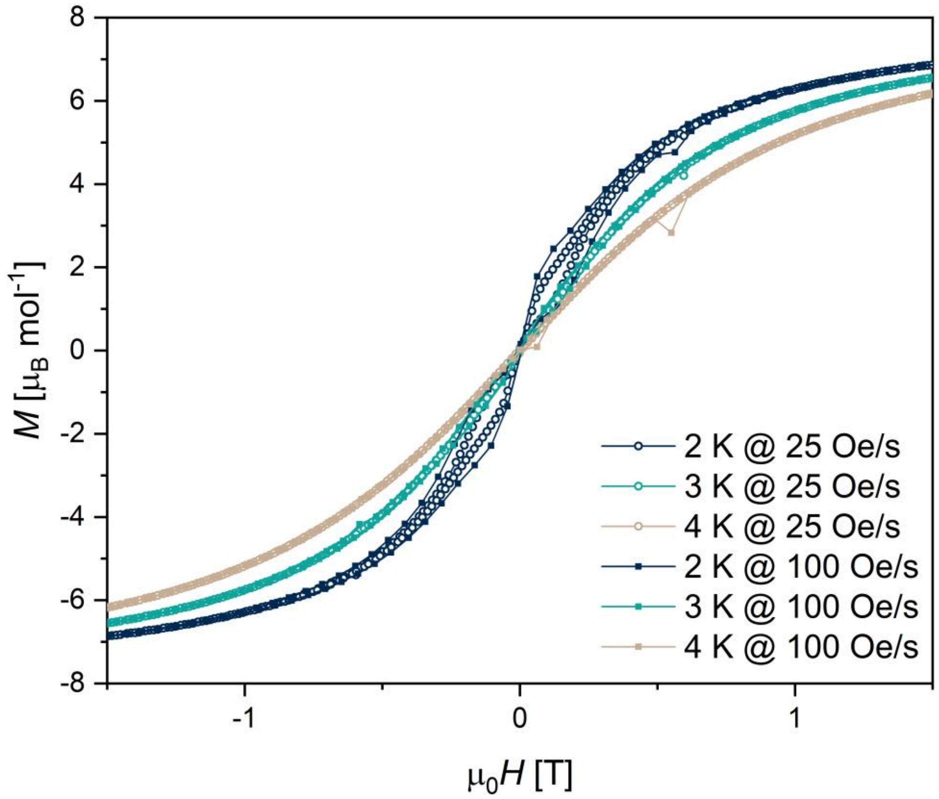

Fig. S 42: Hysteresis loops collected between -1.5 and 1.5 T at different temperatures and two different sweep rates of the magnetic field for **4-Dy**. At 2 K the loop is still open, while already closed at 3 K.

7. Quantum Chemical Calculations

The quantum chemical RI-DFT calculations were performed by means of the program system TURBOMOLE[12] using the RI-BP86 functional.[13-15] The basis sets for each atom were of def-SV(P) quality.[16, 17] For Sm[18] effective core potentials (ecp) containing 51 electrons including the f-electrons were taken. Population analyses based on occupation numbers were performed to calculate shared electron numbers (SEN) as reliable measures for covalent bonding. [19, 20]

Cartesian Coordinates of the molecules under discussion (given in a.u.) are summarized in a separated file.

8. References

1. S. M. Cendrowski-Guillaume, G. Le Gland, M. Nierlich and M. Ephritikhine, *Organometallics*, 2000, **19**, 5654-5660.
2. P. Girard, J. L. Namy and H. B. Kagan, *J. Am. Chem. Soc.*, 1980, **102**, 2693-2698.
3. J. Hicks, P. Vasko, J. M. Goicoechea and S. Aldridge, *Nature*, 2018, **557**, 92-95.
4. F. Krämer, M. S. Luff, U. Radius, F. Weigend and F. Breher, *Eur. J. Inorg. Chem.*, 2021, **2021**, 3591-3600.
5. G. Sheldrick, *Acta Cryst. A*, 2015, **71**, 3-8.
6. G. Sheldrick, *Acta Cryst. C*, 2015, **71**, 3-8.
7. O. V. Dolomanov, L. J. Bourhis, R. J. Gildea, J. A. K. Howard and H. Puschmann, *J. Appl. Crystallogr.*, 2009, **42**, 339-341.
8. D. Kratzert, J. J. Holstein and I. Krossing, *J. Appl. Crystallogr.*, 2015, **48**, 933-938.
9. D. Kratzert and I. Krossing, *J. Appl. Crystallogr.*, 2018, **51**, 928-934.
10. F. Aquilante, J. Autschbach, A. Baiardi, S. Battaglia, V. A. Borin, L. F. Chibotaru, I. Conti, L. De Vico, M. Delcey, I. Fdez. Galván, N. Ferré, L. Freitag, M. Garavelli, X. Gong, S. Knecht, E. D. Larsson, R. Lindh, M. Lundberg, P. Å. Malmqvist, A. Nenov, J. Norell, M. Odelius, M. Olivucci, T. B. Pedersen, L. Pedraza-González, Q. M. Phung, K. Pierloot, M. Reiher, I. Schapiro, J. Segarra-Martí, F. Segatta, L. Seijo, S. Sen, D.-C. Sergentu, C. J. Stein, L. Ungur, M. Vacher, A. Valentini and V. Veryazov, *J. Chem. Phys.*, 2020, **152**, 214117.
11. N. F. Chilton, R. P. Anderson, L. D. Turner, A. Soncini and K. S. Murray, *J. Comput. Chem.*, 2013, **34**, 1164-1175.
12. TURBOMOLE, *V7.6 2021, a development of University of Karlsruhe and Forschungszentrum Karlsruhe GmbH*, 1989-2007, TURBOMOLE GmbH, since 2007; available from https://www.turbomole.org.
13. J. P. Perdew, *Phys. Rev. B*, 1986, **34**, 7406-7406.
14. J. P. Perdew, *Phys. Rev. B*, 1986, **33**, 8822-8824.
15. A. D. Becke, *Phys. Rev. A*, 1988, **38**, 3098-3100.
16. F. Weigend and R. Ahlrichs, *Phys. Chem. Chem. Phys.*, 2005, **7**, 3297-3305.
17. F. Weigend, *Phys. Chem. Chem. Phys.*, 2006, **8**, 1057-1065.
18. M. Dolg, H. Stoll, A. Savin and H. Preuss, *Theor. Chim. Acta*, 1989, **75**, 173-194.
19. R. Heinzmann and R. Ahlrichs, *Theor. Chim. Acta*, 1976, **42**, 33-45.
20. R. Ahlrichs and C. Ehrhardt, *Chem. Unserer Zeit*, 1985, **19**, 120-124.
